# Clues from an ionic cocrystal structure: from catalysis to mechanochemistry†

**DOI:** 10.1039/d4ra05652c

**Published:** 2024-11-04

**Authors:** Bandana Sar, Mollah Rohan Ahsan, Arijit Mukherjee

**Affiliations:** a Department of Chemistry Birla Institute of Science and Technology (BITS) Pilani Hyderabad campus Hyderabad 500078 Telangana India arijit.mukherjee@hyderabad.bits-pilani.ac.in

## Abstract

A crystal structure is no longer conceived as a static entity; rather, it often mirrors crystallization pathways, linking crystal structures with a solution scenario. In this study, taking a clue from a previously reported ionic cocrystal structure, an *in situ* acetic acid-based catalytic protocol is developed for the *N*-acylation of amines using ester sources. In addition to better catalytic efficiency, this *in situ* approach was extended further to a mechanochemistry protocol in the context of a multicomponent reaction. This points towards a broader applicability of crystal structures that goes beyond the domain of structural chemistry and delves into catalysis and mechanosynthesis.

## Abstract 2

A crystal structure is no longer conceived as a static entity; rather, it often mirrors crystallization pathways, linking crystal structures with a solution scenario.

## Graphical abstract

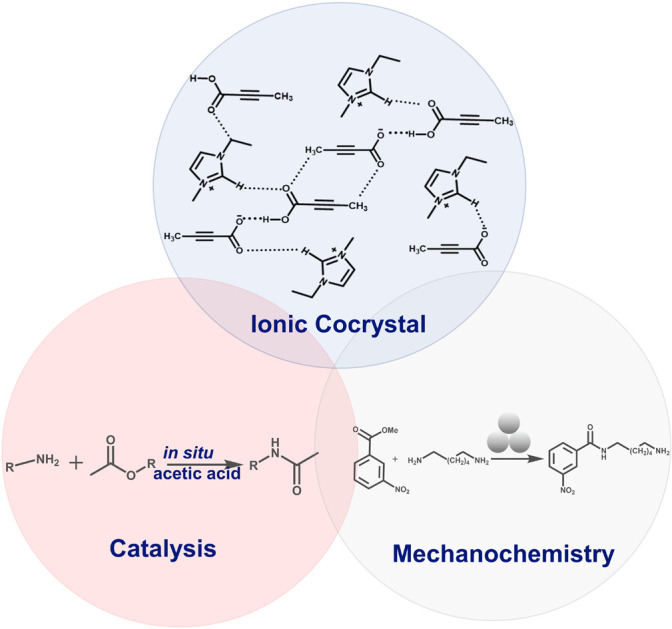

Crystal structures are historically viewed as static entities, mainly used for determining atomic coordinates and identifying intermolecular interactions.^1^ The modern notion of crystals evolved with the advancement of supramolecular chemistry^2^ and the description of crystals as “supermolecule par excellence”,^3,4^ has contributed immensely to the purpose.^5–10^ Despite some significant early milestones correlating crystal structures with chemical reactivity,^3,11^ the perception of some crystal structures being “crystallization snapshots” took time to emerge.^12,13^ It is only recently, with the concept of crystal structure landscape,^5,7,12^ that crystal structures are conceived as data points in a given structural landscape and can, therefore, be linked with crystallization pathways.^13^

In 2006, Davey and co-workers showed, with the example of tetrolic acid (TA), that polymorphism in a given system can be linked with solution structures.^14–16^ Later, while investigating TA crystallization from ionic liquids, Myerson and co-workers identified that the metastable (*α*) form could be obtained while crystallized from hydrophobic ionic liquids.^17^ Using a polar ionic liquid 1-ethyl-3-methylimidazolium acetate ([Emim]^+^[OAc]^−^) as a crystallization medium, however, led to an ionic cocrystal which was crystallized in *P*1̄, with cell parameters of *a* = 6.746(3) Å, *b* = 7.246(3) Å, *c* = 7.434(3) Å; *α* = 77.801(7)°, *β* = 83.650(8)°, *γ* = 88.409(7)°.^17^ The TA molecules form a catemeric chain, and the respective C–O bond distances were found to be 1.279(2) Å and 1.221(2) Å with ΔC–O ∼0.058 Å. This lowering of ΔC–O could be attributed to the partial deprotonation in the ionic cocrystal.^18^ Henceforth, this ionic cocrystal is denoted as [TA]·[Emim]^+^ [TA]^−^ with a neutral TA, a deprotonated TA, and a disordered [Emim]^+^ cation in the asymmetric unit (Fig. 1).^17^

**Fig. 1:**
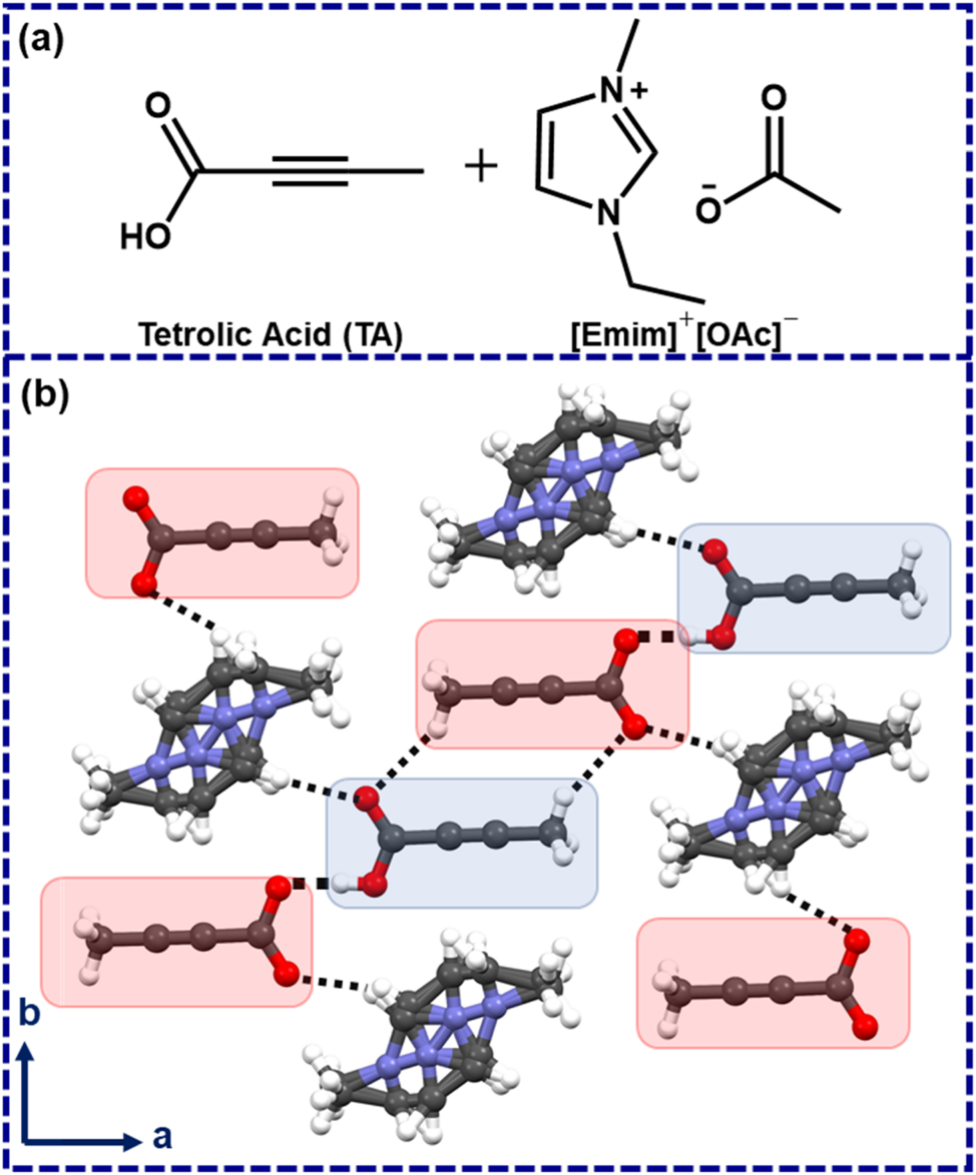
(a) Chemical diagrams of tetrolic acid and 1-ethyl-3-methylimidazolium acetate, (b) the crystal structure of TA×[Emim]^+^ [TA]^−^ (protonated and deprotonated TA are marked with blue and red patches). Blue: nitrogen, red: oxygen, gray: carbon, white: hydrogen.

While the disorder [Emim]^+^ may be understood from its mobile nature, the partially deprotonated TA, however, implies that the proton could be abstracted by the [OAc]^−^anion of the used ionic liquid ([Emim]^+^[OAc]^−^), which may lead to the formation of acetic acid in the crystallization medium. In hindsight, we felt that this structure might represent a solution scenario, and *in situ* generated acetic acid in this way might be employed as a catalyst in a suitable organic reaction.

*N*-acylation of amines has remained an active research topic for years for the various applications of amide derivatives in several industries.^19^ The traditional methods, however, rely heavily upon toxic and hygroscopic activated-acylating agents.^20^ Other procedures employed metal catalysts,^21^ some of which are often expensive and may not be viable industrially.^22,23^ In a study in 2017, Williams and co-workers demonstrated the role of acetic acid as a catalyst in the *N*-acylation of amines using esters as the acyl source.^24^ In hindsight, we felt such reactions would be suitable for investigating the role of *in situ*-generated acetic acid as a catalyst, as was expected from the given ionic cocrystal structure.

The first question that one encounters here is whether the crystal structure of [TA]·[Emim]^+^ [TA]^−^ represents a similar solution scenario. To investigate this, FTIR and ^1^H NMR analysis was performed for TA, acetic acid, [Emim]^+^[OAc]^−^, and a 6 M solution of TA in [Emim]^+^[OAc]^−^ (a mixture of TA (3 mg, one equivalent) and [Emim]^+^[OAc]^−^ (6 μl, one equivalent)) (Fig. 2). From the reaction perspective, this amounts to ∼1 mol% of the limiting reagent (ESI-S1(b and c)†).

**Fig. 2:**
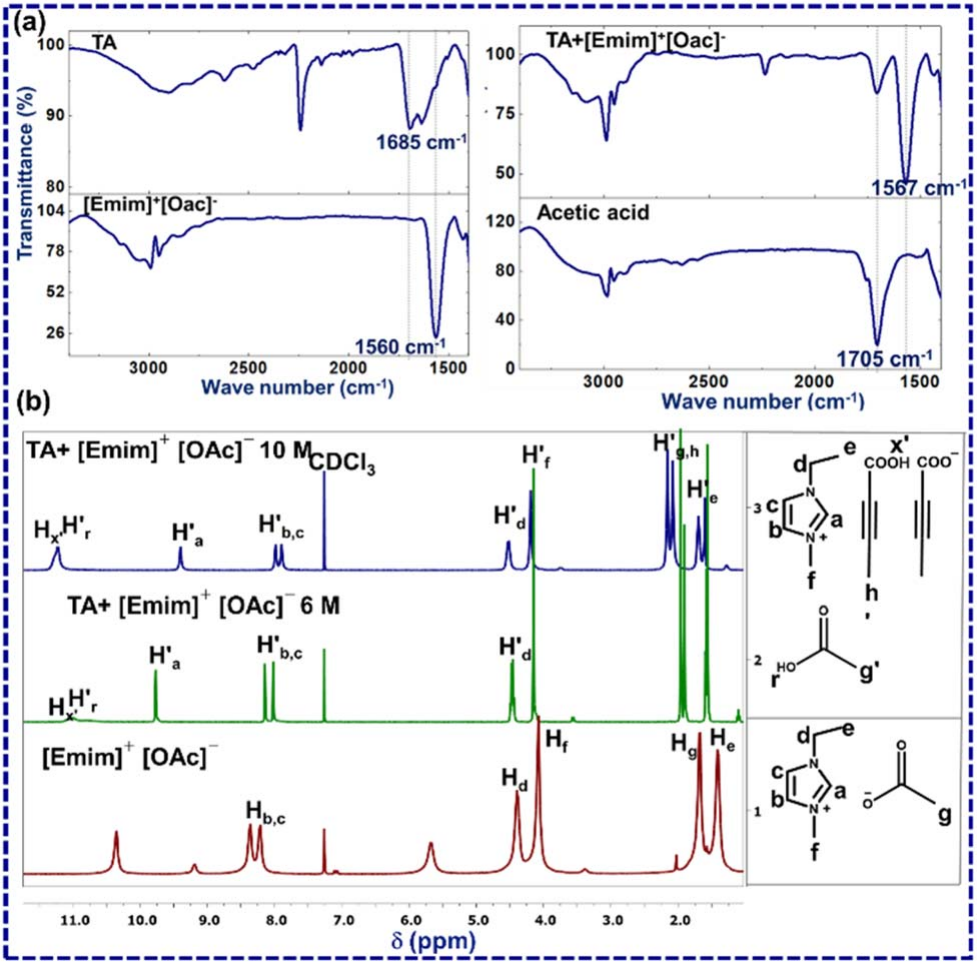
(a) ATR-FTIR-spectra of [Emim]^+^[OAc]^−^ (red), TA (blue), acetic acid (black), TA–[Emim]^+^[OAc]^−^ mixture (cyan); (b) ^1^H NMR of [Emim]^+^[OAc]^−^ (red) and TA–[Emim]^+^[OAc]^−^mixture(cyan).

A new peak at 1705 cm^−1^ in ATR-FTIR for TA and [Emim]^+^[OAc]^−^ mixture may be assigned to acetic acid (ESI-S2(d)†). This was further confirmed by collecting the FTIR spectrum through the KBr method (ESI-S2(c)†). The splitting of a sharp carbonyl peak may be attributed to the relative positions of TA and acetic acid. ^1^H NMR experiments for TA, [Emim]^+^[OAc]^−^ and TA–[Emim]^+^[OAc]^−^ mixture were carried out through a capillary method using CDCl_3_ as a lock-solvent to further support the insights derived from the FTIR analysis (ESI-S2(b)†). The dilution experiments performed through ^1^H NMR indicated the presence of acetic acid in 6 M and 10 M concentrations, as may be expected from the (1 : 1) molar ratio of TA and [Emim]^+^[OAc]^−^. The variation of these peak positions with dilution indicates the presence of hydrogen bonding in the liquid mixture (ESI-S2(b)†). In a 6 M solution, A new peak was observed at *δ* = 10.9 ppm (
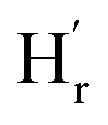
) that may correspond to the carboxylic acid proton of acetic acid. These insights, as obtained from the ^1^H NMR and FTIR spectra, demonstrate the *in situ* generation of acetic acid when TA is taken together with [Emim]^+^[OAc]^−^ in (1 : 1) molar ratio as suggested by the ionic cocrystal structure. This observation also corroborates well with a previous study by Rogers and co-workers, where the generation of acetic acid was observed by heating a mixture of [Emim]^+^ [OAc]^−^ and azole compounds.^25^

The next question in this regard was related to whether this *in situ* generated acetic acid could be used as a catalyst in the *N*-acylation of amines. If successful, it may offer advantages over the existing methods due to being a non-metal catalytic method, using the lower reactive acetate esters as compared to acid chlorides and acid anhydrides and generating acetic acid only through a dynamic equilibrium.

To investigate if the reaction was catalyzed by the *in situ* generated acetic acid, a blank reaction was first performed with 1 mmol benzylamine and 1 ml of EtOAc at 80 °C for 20 hours, only a 3.8 ± 0.7% of 1a was produced (ESI-S3(a)†). While the reaction was carried out for 20 hours with [Emim]^+^[OAc]^−^ (10 mol% with respect to benzylamine in the absence of TA, a 17.6 ± 0.3% yield was observed (ESI-S3(b)†). On the other hand, 25.6 ± 1.1% of 1a was obtained when TA (10 mol% with respect to benzylamine) was added to the reaction medium in the absence of [Emim]^+^[OAc]^−^(ESI-S3(c)†). This may be attributed to the competition between formations of salts *vs.* amides, as often observed with organic acids.^26^ These results indicate that a facile *N*-acetylation could not be carried out in the presence of either TA or [Emim]^+^[OAc]^−^, with sufficient yield. The reaction yield, however, was strikingly increased to 100% when [Emim]^+^[OAc]^−^ (10 mol%) and TA (10 mol%) were used together in the reaction mixture. When benzylamine was refluxed with EtOAc at 80 °C for 20 hours in the presence of TA and [Emim]^+^[OAc]^−^), 1a was produced with 100% yield (ESI-S4(a)†). To investigate the role of reaction time, the reaction was carried out for different intervals ranging between 4 and 16 hours. The yield of 1a varied between 18% and 86% during those intervals (Table 1) (ESI-S4(a)†). More significantly, 1a was obtained in 97.5 ± 1.8% yield even when the catalytic load was reduced from 10% to 1% (ESI-S4(b)†). (Table 1). A comparison between this method and the previous method (ESI-S11†) shows a greater efficiency of the *in situ* technique over the previous methods.

**Table 1:** Catalytic role of TA and [Emim]^+^[OAc]^−^ mixture in *N*-acetylation reaction of benzylamine and ethyl acetate and optimization of reaction conditions

Entry^a^	Time (hours)	TA (mol%)	[Emim]^+^[OAc]^−^(mol%)	Conversion^b^ (%)
1	20	0	0	3.8 ± 0.9
2	20	0	10	17.6 ± 0.5
3	20	0	1	6.6 ± 1.4
4	20	10	0	25.6 ± 2
5	20	1	0	19.6 ± 0.5
6	4	10	10	16.9 ± 1.5
7	8	10	10	44.9 ± 2.6
8	12	10	10	66 ± 4.6
9	16	10	10	89.3 ± 3.1
10	20	10	10	100 ± 0
11	20	1	1	98.3 ± 2.9

a Reactions performed with 10 mol% catalysts were carried out on a 1 mmol scale, and entries 3, 5, and 11 were performed on a 4 mmol scale (ESI-S1).

b Conversions were analyzed by the ^1^H NMR spectra (ESI-S3 and S4).

Subsequently, the scope of the above approach was investigated toward variegated amines to check the generality as well as variation of yield% with electronic factors. Some of the systems chosen here are similar to the previous studies^24^ to strike a direct comparison between the two approaches. In the presence of an electron-withdrawing group (such as –Br in 1b and –CF_3_ in 1c), the yield of 1b and 1c was found to be ∼91 ± 0.8% and ∼43.7 ± 1.8% (ESI-S5(a and b)†), respectively, when the reaction was performed with 1 mol% catalytic load for 20 hours at 80 °C, indicating the effectiveness of the approach even in slightly deactivated systems.

The chemoselectivity of the reaction was also investigated, and the desired product 1d was obtained in quantitative yield. The peak at 1633 cm^−1^ in FTIR-ATR confirmed the formation of an amide bond and proved the chemoselectivity of the reaction (ESI-S5(c)†). Since more vigorous conditions may be required for secondary amines, butyl acetate (b.p. = 126 °C) was used during the synthesis of 1e (with 1 mol% catalyst), and the reaction was performed at 115 °C. Under these conditions, 1e was formed with a 99.3 ± 0.5% yield after 24 hours (ESI-S5(d)†).

Further, when more deactivated aromatic amines were employed as one of the reactants, the reactions were performed at 115 °C for 24 hours using butyl acetate and with 1 mol% TA and 1 mol% [Emim]^+^[OAc]^−^. Respective products were formed in quantitative yield in most of the cases (2a, 2b, 2c, 2d, 2e; ESI-S6(a–e)†). In the synthesis of 2g, however, the yield was reduced to 41 ± 1.6% due to additional deactivation by a pyridine ring (ESI-S6(g)†). This method was also successful in synthesizing phenacetine (2f), an anti-analgesic API, with a 98.7 ± 1.0% yield (ESI-S6(f)†). In the next step, to check the variability of the approach, respective acetylating agents were changed from aliphatic to aromatic esters (methyl benzoate and methyl 3-nitrobenzoate). Under the same reaction conditions (with 1 mol% catalyst at 80 °C for 20 hours) using toluene as a solvent, 90.7 ± 1.4% of 3a and 89 ± 0.8% of 3b were obtained, respectively (ESI-S7(a and b)†) (Fig. 3).

**Fig. 3:**
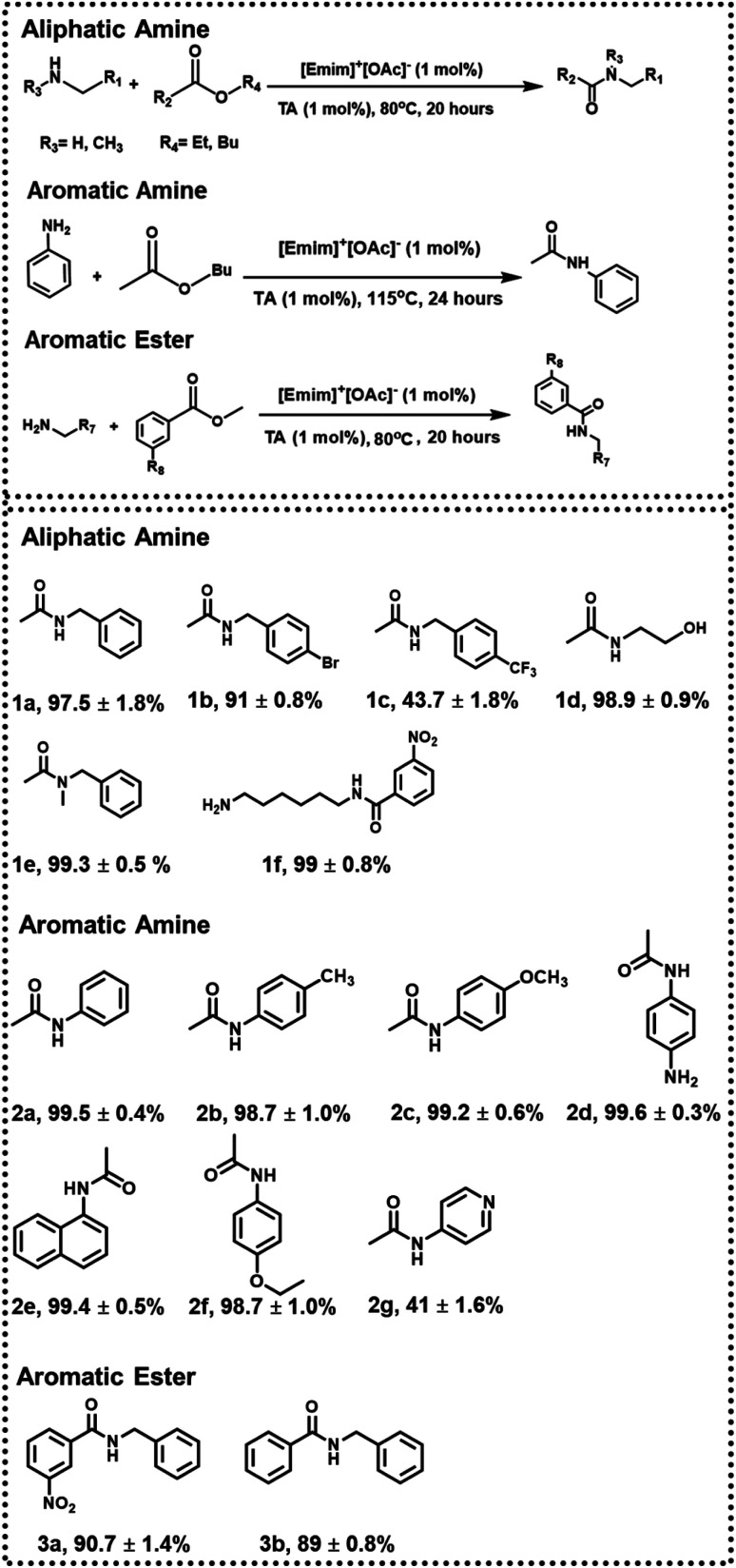
*N*-Acetylation of amines with 1 mol% catalytic load.

From a mechanistic viewpoint, a weak acid like acetic acid is preferred as a catalyst in this reaction compared to strong acids (such as sulfuric acid) due to its preference for amide formation over salt formation, as shown before.^24,27^ Apart from its role in acid catalysis, acetic acid also plays a pivotal role in forming the respective transition state.^26^ Hence, it may be possible that replacing direct acetic acid catalysis with an *in situ* approach lowers the requirement of minimum catalytic load (from 10 to 1 mol%) as an *in situ* process dictates an equilibrium between the cyclic intermediate^26^ and acetic acid formation and enables a controlled release of acetic acid. Other organic acids were also used as catalysts to test this hypothesis, *e.g.*, oxalic acid (OA) and malonic acid (MA). In the presence of these strong acids (OA (p*K*_a_ = 1.2) and MA (p*K*_a_ = 2.83)) as catalysts, the salt formation pathway was favored, and only 17% and 27% 1a were formed, respectively (ESI-S8(a and b)†). 1a, however, was still formed with 100% yield when these two strong acids were mixed with [Emim]^+^[OAc]^−^ (ESI-S8(c and d)†). This indicates solid organic acids with a p*K*_a_ lower than acetic acid may be used with [Emim]^+^[OAc]^−^ to produce *in situ* acetic acid. This observation opens up an opportunity to apply it to mechanochemistry.^28,29^

In the next step, we tried to extend the above approach to a mechanochemistry protocol. When hexamethylenediamine and methyl 3-nitrobenzoate were ground together in a mixer ball mill for 60 minutes at a frequency of 30 Hz in a 10 ml jar with a single 10 mm steel ball, without a catalyst, no product was formed (ESI-S9(a and b)†). In the presence of TA and [Emim]^+^[OAc]^−^ mixture with the above conditions, 1f was formed with 100% yield (ESI-S9(c)†). The experimental parameters were varied further for grinding conditions and catalytic load to check the robustness of the protocol. 1f was formed exclusively (ESI-S9(d–f)† and (Table 2)). Since 1f formation dictates one –NH_2_ group of hexamethylenediamine being converted to an amide with the other –NH_2_ remaining free, we thought to further utilize this mechanosynthesis protocol in a multicomponent reaction.

**Table 2:** Mechanochemical *N*-acetylation reaction

Reactant	Molar ratio	Catalyst (mol%)	Liquid-assisted grinding (LAG)					Product	Yield (%)
			Time (min)	Frequency (Hz)	Jar (mL)	No. of ball (10 mm)	*η* (μl mg^−1^)		
A + B	(1 : 1)	0	60	30	10	1	0	NIL	0
A + B	(2 : 1)	0	60	30	10	1	1.81	NIL	0
A + B	(1 : 1)	30	60	30	10	1	1.81	1f	100
A + B	(1 : 1)	15	30	30	10	1	1.81	1f	100
A + B	(1 : 1)	5	30	30	10	1	1.81	1f	100
A + B	(2 : 1)	30	90	30	10	2	1.81	1f	100
A + B + C	(1 : 1 : 1)	5	30	30	10	1	1.81	4a	56
A + B + D	(2 : 2 : 1)	5	30	30	10	1	1.81	4b	87

When methyl 3-nitrobenzoate, hexamethylenediamine, and 3,5-dichlorobenzaldehyde were ground at 30 Hz in a 10 ml jar with one 10 mm ball and 5 mol% catalyst, 4a was formed with a significant yield (ESI-S9(g)†). Next, 4b was synthesized when terephthalaldehyde was used instead of 3,5-dichlorobenzaldehyde in the above protocol (ESI-S9(h)†) (Fig. 4).

**Fig. 4:**
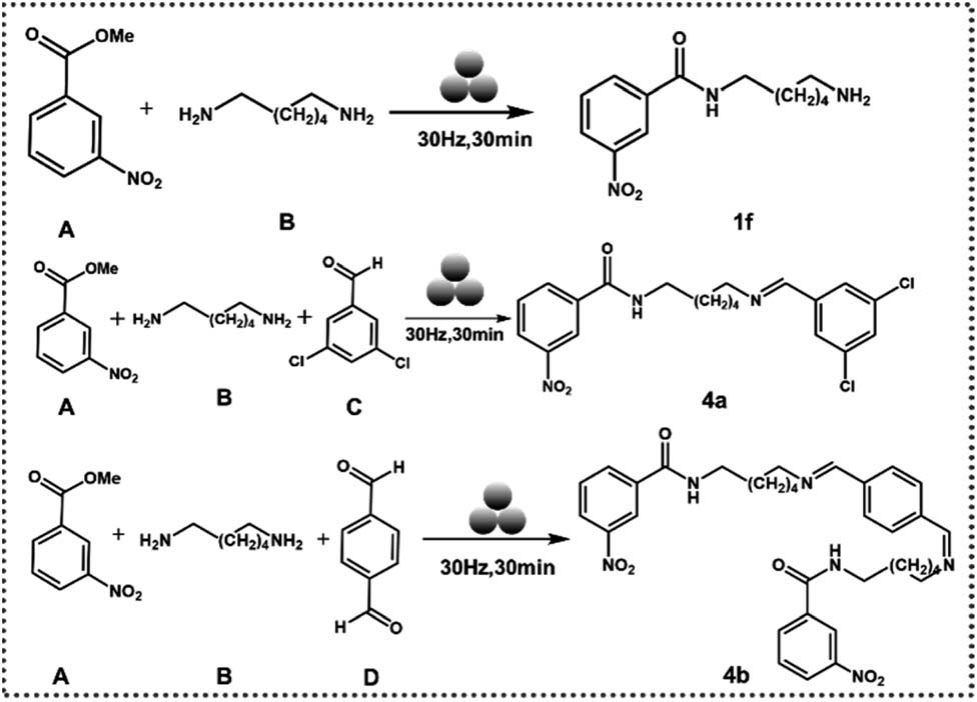
Reaction adopted for mechanosynthesis of amides.

In conclusion, a clue from a previously reported ionic cocrystal structure leads to the development of a robust protocol for *in situ* acetic acid catalysis for amide synthesis. The protocol was generalized with many examples of varying electronic and structural factors, including modifying the acylating agent from aliphatic to aromatic esters. Apart from showing catalytic efficiency, this protocol could be extended to the mechanosynthesis of amides and a mechanochemical multicomponent reaction. Given the importance of amide derivatives in organic synthesis and medicinal chemistry, this robust protocol is expected to diversify the synthesized substrates through a greener route. In the end, this study shows that crystal structures, which were once conceived as “chemical cemeteries,”^30,31^ can provide meaningful insights to develop intriguing and useful protocols relevant to the other areas of the subject, such as greener synthesis,^32^ or organocatalysis.^33^

## Data availability

The data supporting this article have been included in the ESI.†

## Author contributions

BS: data curation, formal analysis, investigation, methodology; MRA: validation, visualization, software, writing – original draft preparation; AM: conceptualization, project administration, funding acquisition, writing – review & editing, supervision.

## Conflicts of interest

There are no conflicts to declare.

## Supplementary Material

RA-014-D4RA05652C-s001
