# Supplementary material for: Clues from an ionic cocrystal structure: from catalysis to mechanochemistry
Source: RSC Adv. 2024 Nov 4;14(47):34843–7. doi: 10.1039/d4ra05652c (PMC11534070; doi:10.1039/d4ra05652c)
Supplement: RA-014-D4RA05652C-s001 [file RA-014-D4RA05652C-s001.pdf]

Supplementary Information (SI) for RSC Advances.
This journal is © The Royal Society of Chemistry 2024

Clues from an ionic cocrystal structure: From catalysis to mechanochemistry

Bandana Sar,[a] Mollah Rohan Ahsan,[a] and Arijit Mukherjee *[a]

Table of contents:

Sl. No.	Content	Page number
**S1**	Materials, methods, and calculations	**2-6**
**S2**	NMR spectra and FTIR spectra of generation of in-situ acetic acid	**5-11**
**S3**	Blank reactions	**11-15**
**S4**	Reactions with catalyst	**15-17**
**S5**	Reactions of aliphatic amines	**17-22**
**S6**	Reactions of aromatic amines	**22-29**
**S7**	Reactions of amines with aromatic esters	**29-31**
**S8**	Reaction catalyzed by other strong acids in combination with $[Emim]^+[OAc]^-$	**31-35**
**S9**	N-acetylation reactions in mechanochemistry	**35-46**
**S10**	Average Reaction yield and standard deviation	**46-48**
**S11**	Comparison of this in-situ acetic acid catalyzed N-acylation of amines with other methods	**48**
**S12**	Catalyst separation and recyclability	**49-50**

Materials and methods

Compounds used in this study were ethyl acetate, butyl acetate, methyl benzoate, methyl-3-nitrobenzoate, benzylamine, 4-bromobenzylamine, 4-(trifluoromethyl) benzylamine, ethanolamine, N-methyl-1-phenylmethanamine, aniline, p-toluidine, p-anisidine, phenylenediamine, 1-napthylamine, 4-ethoxyaniline, 4-aminopyridine, hexamethylenediamine, terephthalaldehyde, 3,5-dichlorobenzaldehyde, tetrolic acid, malonic acid, oxalic acid, 1-ethyl-3-methylimidazolium acetate ($[Emim]^+[OAc]^-$). Tetrolic acid, oxalic acid, N-methyl-1-phenylmethanamine, aniline, and 1-ethyl-3-methylimidazolium acetate were purchased from BLD Pharm. Ethyl acetate and benzyl amines were purchased from Avra, and butyl acetate was purchased from TCI. The remaining other chemicals are purchased from Sigma-Aldrich.

S1. Calculations

(a) Limiting reagent determination:

(i) Solution chemistry:

Reaction of benzylamine with ethyl acetate

The molecular weight of benzylamine is 107.16 g/mol

The volume of benzylamine taken is 109 µl, which is equivalent to 107 mg of benzylamine

The number of moles of benzylamine taken ≈ 0.001 mol

The molecular weight of ethyl acetate is 88.11 g/mol

The volume of ethyl acetate taken was1000 µl, which is equivalent to 902 mg of ethyl acetate

The number of moles of ethyl acetate taken was ≈ 0.01 mol

Ethyl acetate is used in excess, and benzylamine is the limiting reagent for the reaction.

Similarly, in other reactions, aliphatic and aromatic amines are the limiting reagents, while the ester used as the acyl source is in excess.

(ii) Mechanochemistry

Reaction of hexamethylenediamine with methyl-3-nitrobenzoate in (1:2) ratio:

The molecular weight of hexamethylenediamine is 116.21 g/mol

The weight of hexamethylenediamine taken was 29 mg

The number of moles of hexamethylenediamine taken was ≈ 0 .00025 mol

The molecular weight of methyl-3-nitrobenzoate is 181.14 g/mol

The weight of methyl-3-nitrobenzoate taken was 90.5 mg

The number of moles of methyl-3-nitrobenzoate taken was ≈ 0.0005 mol

methyl-3-nitrobenzoate is used in excess, and hexamethylenediamine is the limiting reagent for the reaction.

Reaction of hexamethylenediamine with methyl-3-nitrobenzoate in (1:1) ratio:

The molecular weight of hexamethylenediamine is 116.21 g/mol

The weight of hexamethylenediamine taken was 29 mg

The number of moles of hexamethylenediamine taken was ≈ 0 .00025 mol

The molecular weight of methyl-3-nitrobenzoate is 181.14 g/mol

The weight of methyl-3-nitrobenzoate taken was 45.2 mg

The number of moles of methyl-3-nitrobenzoate taken was ≈ 0.00025 mol

Both the reactants were used in the same mole ratio. Therefore, one of them can be considered as the limiting reagents.

(b) Mol% Calculation of tetrolic acid (TA)

(i) 30 mol% calculation:

$$mol\% \ of \ catalyst = \frac{number \ of \ moles \ of \ catalyst}{number \ of \ moles \ of \ catalyst \ + \ number \ of \ moles \ of \ limiting \ reagent} \times 100$$

The molecular weight of TA is 84.07 g/mol

The number of moles of TA taken was $\frac{8.9 \ mg}{84 \ g/mol}$ = 0.0001mol

The no. of moles of limiting reagent (*methyl-3-nitrobenzoate*) taken for this reaction was 0.00025 mol

Mol% of catalyst used = $\frac{0.0001}{0.0001 \ + \ 0.00025} \times$ 100= 28.5 ≈ 30%

(ii) 15 mol% calculation:

$$mol\% \ of \ catalyst = \frac{number \ of \ moles \ of \ catalyst}{number \ of \ moles \ of \ catalyst \ + \ number \ of \ moles \ of \ limiting \ reagent} \times 100$$

The molecular weight of TA is 84.07 g/mol

The number of moles of TA taken was $\frac{4 \ mg}{84 \ g/mol}$ = 0.000047 mol

The no. of moles of limiting reagent (*methyl-3-nitrobenzoate*) taken for this reaction was 0.00025 mol

Mol% of catalyst used = $\frac{0.000047}{0.000047\ +\ 0.00025} \times 100$= 15.8% ≈ 15%

(iii) 10 mol% calculation

$$mol\%\ of\ catalyst = \frac{number\ of\ moles\ of\ catalyst}{number\ of\ moles\ of\ catalyst\ +\ number\ of\ moles\ of\ limiting\ reagent} \times 100$$

The molecular weight of TA is 84.07 g/mol.

The number of moles of TA taken was $\frac{8.4\ mg}{84\ g/mol}$ = 0.0001 mol

The no. of moles of limiting reagent (benzylamine) taken for this reaction was 0.001 mol

Mol% of catalyst used = $\frac{0.0001}{0.0001\ +\ 0.001} \times 100$= 9.09 ≈ 10%

(iv) 5 mol% calculation:

$$mol\%\ of\ catalyst = \frac{number\ of\ moles\ of\ catalyst}{number\ of\ moles\ of\ catalyst\ +\ number\ of\ moles\ of\ limiting\ reagent} \times 100$$

The molecular weight of TA is 84.07 g/mol

The number of moles of TA taken was $\frac{1.2\ mg}{84\ g/mol}$ = 0.0000142 mol

The no. of moles of limiting reagent (*methyl-3-nitrobenzoate*) taken for this reaction was 0.00025 mol

Mol% of catalyst used = $\frac{0.0000142}{0.0000142\ +\ 0.00025} \times 100$ = 5.3% ≈ 5%

(v) 1 mol% calculation: (for 1 mmol of limiting reagent)

$$mol\%\ of\ catalyst = \frac{number\ of\ moles\ of\ catalyst}{number\ of\ moles\ of\ catalyst\ +\ number\ of\ moles\ of\ limiting\ reagent} \times 100$$

The molecular weight of TA is 84.07 g/mol

The number of moles of TA taken was $\frac{0.8\ mg}{84\ g/mol}$ = 0.0000095mol

The no. of moles of limiting reagent (benzylamine) taken for this reaction was 0.001 mol

Mol% of catalyst used = $\frac{0.0000095}{0.0000095\ +\ 0.001} \times 100$ = 0.85 ≈ 1%

(for 4 mmol of limiting reagent)

$$mol\% \; of \; catalyst = \frac{number \; of \; moles \; of \; catalyst}{number \; of \; moles \; of \; catalyst \; + \; number \; of \; moles \; of \; limiting \; reagent} \times 100$$

The molecular weight of TA is 84.07 g/mol

The number of moles of TA taken was $\frac{3.3 \; mg}{84 \; g/mol}$ = 0.000039mol

The no. of moles of limiting reagent (benzylamine) taken for this reaction was 0.004 mol.

Mol% of catalyst used = $\frac{0.000039}{0.000039 \; + \; 0.004} \times 100$ = 0.97 ≈ 1%

(c) Mol% Calculation of ($[Emim]^+[OAc]^-$)

(i) 30 mol% calculation:

mol% of catalyst = (Number of moles of catalyst × 100) / (Number of moles of catalyst + Number of moles of limiting reagent)

The molecular weight of $[Emim]^+[OAc]^-$ is 170.21 g/mol

The volume of $[Emim]^+[OAc]^-$ taken was 15 µl=16.5 mg

Number of moles of $[Emim]^+[OAc]$ taken was 9.7×10^{-5} mol

The no. of moles of limiting reagent (*methyl-3-nitrobenzoate)* taken for this reaction was 0.00025 mol

Mol% of catalyst used = $\frac{9.7 \times 10 - 5}{9.7 \times 10 - 5 + \; 0.00025 \; mol} \times 100$ = 27.9 ≈ 30%

(ii) 15 mol% calculation:

mol% of catalyst = (Number of moles of catalyst × 100) / (Number of moles of catalyst + Number of moles of limiting reagent)

The molecular weight of $[Emim]^+[OAc]^-$ is 170.21 g/mol

The volume of $[Emim]^+[OAc]^-$ taken was 9 µl = 9.9 mg

Number of moles of $[Emim]^+[OAc]$ taken was 5.3×10^{-5} mol

The no. of moles of limiting reagent (*methyl-3-nitrobenzoate)* taken for this reaction was 0.00025 mol

Mol% of catalyst used = $\frac{5.3 \times 10 - 5}{5.3 \times 10 - 5 + \; 0.00025 \; mol} \times 100$ = 16.6 ≈ 15%

(ii) 10 mol% calculation:

mol% of catalyst = (Number of moles of catalyst × 100) / (Number of moles of catalyst + Number of moles of limiting reagent)

The molecular weight of $[Emim]^+[OAc]^-$ is 170.21 g/mol

The volume of $[Emim]^+[OAc]^-$ taken was 15 µl = 16.5 mg

Number of moles of $[Emim]^+[OAc]^-$ taken was 9.69×10^{-5} mol

The no. of moles of limiting reagent (benzylamine) taken for this reaction was 0.001 mol

Mol% of catalyst used = $\frac{9.69 \times 10-5}{9.69 \times 10-5 + 0.001\,mol} \times 100 = 8.83 \approx 9\%$

(iii) 5 mol% calculation:

mol% of catalyst = (Number of moles of catalyst × 100) / (Number of moles of catalyst + Number of moles of limiting reagent)

The molecular weight of $[Emim]^+[OAc]^-$ is 170.21 g/mol

The volume of $[Emim]^+[OAc]^-$ taken was 1.8 µl = 1.98 mg ≈ 2 mg

Number of moles of $[Emim]^+[OAc]^-$ taken was 1.16×10^{-5} mol

The no. of moles of limiting reagent *(methyl-3-nitrobenzoate)* taken for this reaction was 0.00025 mol

mol% of catalyst used = $\frac{1.16 \times 10-5}{1.16 \times 10-5 + 0.00025\,mol} \times 100 = 4.43 \approx 5\%$

(iv) 1 mol% calculation (for 1 mmol of limiting reagent):

mol% of catalyst = (Number of moles of catalyst × 100) / (Number of moles of catalyst + Number of moles of limiting reagent)

The molecular weight of $[Emim]^+[OAc]^-$ is 170.21 g/mol

The volume of $[Emim]^+[OAc]^-$ taken was 1.5 µl = 1.6 mg

Number of moles of $[Emim]^+[OAc]^-$ taken was 9.7×10^{-6} mol

The no. of moles of limiting reagent (benzylamine) taken for this reaction was 0.001 mol

mol% of catalyst used = $\frac{9.7 \times 10-6}{9.7 \times 10-6 + 0.001\,mol} \times 100 = 0.96 \approx 1\%$

for 4 mmol of limiting reagent

mol% of catalyst = (Number of moles of catalyst × 100) / (Number of moles of catalyst + Number of moles of limiting reagent)

The molecular weight of $[Emim]^+[OAc]^-$ is 170.21 g/mol

The volume of $[Emim]^+[OAc]^-$ taken was 6 µl = 6.6 mg

Number of moles of $[Emim]^+[OAc]^-$ taken was 38.8×10^{-6} mol

The no. of moles of limiting reagent (benzylamine) taken for this reaction was 0.004 mol

$$\text{mol\% of catalyst used} = \frac{38.8 \times 10-6}{38.8 \times 10-6 + 0.004\,mol} \times 100 = 0.96 \approx 1\%$$

S2. (a) NMR spectra of TA: The ^{1}H NMR spectra of TA were collected using Bruker (400 MHz) instrument by dissolving 8 mg of the sample in d^6-DMSO. All the corresponding proton peaks of TA are assigned below.

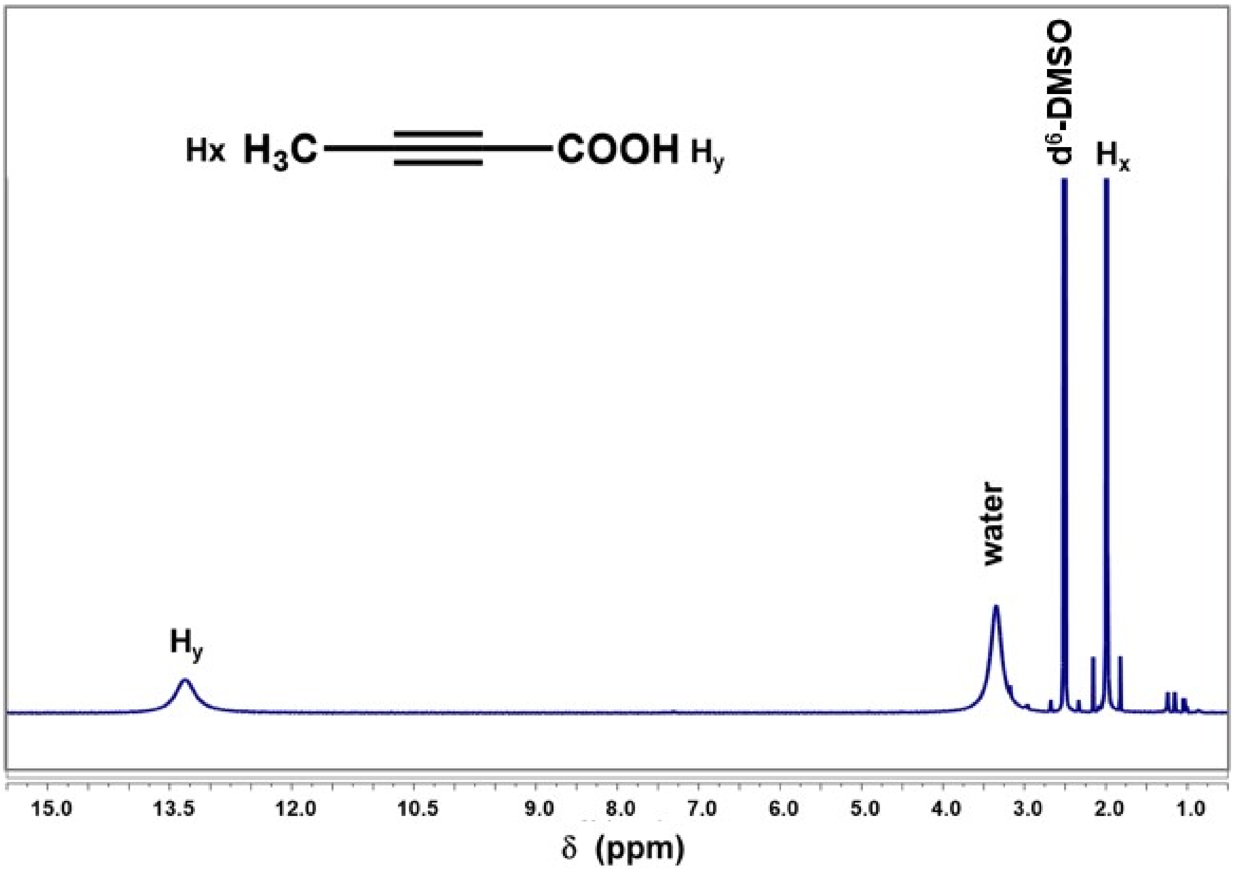

Figure 1: ^{1}H NMR spectra of TA.

(b) Dilution experiments with 1H NMR:

When TA and acetic acid were taken in 6 M concentration in $[Emim]^+[OAc]^-$, Figure 2(a), two peaks appeared at around 10.9 (A) and 11.1 ppm (B). To identify the respective acidic protons, we performed two control experiments, one with excess external acetic acid and one with excess TA, in the mixture of TA and $[Emim]^+$ $[OAc]^-$. When an excess acetic acid (6 equivalent compared to TA) was added to the 6 M TA $[Emim]^+$ $[OAc]^-$ mixture, the intensity of peak A was increased, suggesting it corresponds to acetic acid (Figure 2(b)). Similarly, peak B must correspond to TA. To confirm this, another NMR experiment was performed with excess TA; a solution was prepared with 1 equivalent $[Emim]^+$ $[OAc]^-$, 6 equivalent TA, and 6 equivalent

acetic acid. In contrast to Figure 2(c), where peak A intensity was much higher compared with peak B, in this case, peaks A and B were observed with almost similar intensity, indicating peak B corresponded to TA.

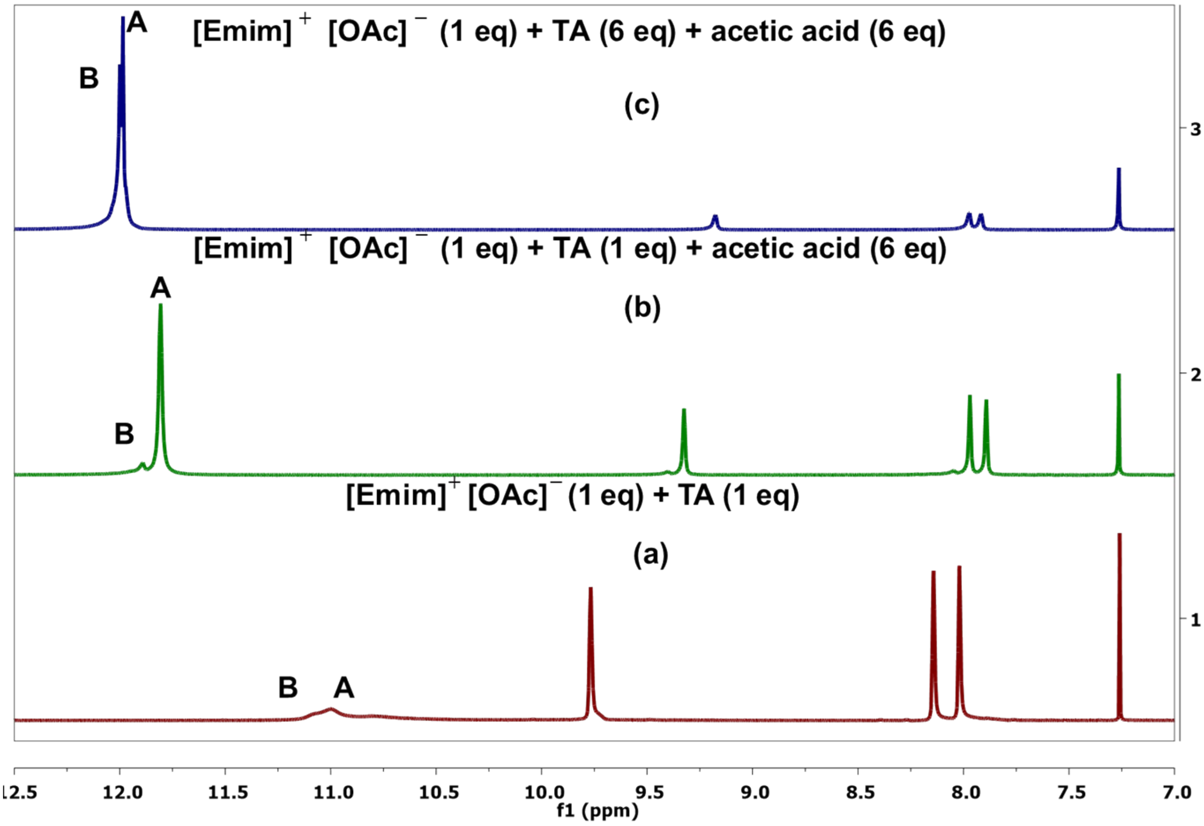

Figure 2: Controlled NMR spectra of TA and [Emim]$^+$ [OAc]$^-$ with excess external acetic acid and excess TA.

In the next step, an NMR dilution experiment was performed through the capillary method to track the extent of acetic acid formation in the TA and [Emim]$^+$ [OAc]$^-$, with varying concentrations. A total of 5 solutions of TA with varying concentrations were prepared in [Emim]$^+$ [OAc]$^-$. Two of the solutions contained excess TA (50 M, 20 M), two of them contained TA comparable with (1:1) TA and [Emim]$^+$ [OAc]$^-$ (6 M, 10M), while one of them contained little amount of TA with excess [Emim]$^+$ [OAc]$^-$ (3 M). In the region of carboxylic acid proton (above 10.5 ppm), a continuous downfield shift was observed with increasing concentration. This indicates the presence of hydrogen bonding formation in this domain, starting at concentrations of 3 M. Contrary to that, a continuous upfield shift was observed in the case of [Emim]$^+$ protons, up to 10 M concentration. The upfield shift in [Emim]$^+$ (9.83, 8.17, 8.05 ppm values at 3 M) up to 10 M was higher compared to the higher

concentrations (from 10 M to 20 M and 30 M). In the equimolar mixture of TA and $[Emim]^+$ $[OAc]^-$, two splitted peaks were observed at 11.0 and 11.13 ppm. This indicates the presence of acetic acid in the equimolar mixture of TA and $[Emim]^+$ $[OAc]^-$. A similar trend was observed in the 10 M solution as well, where the TA and $[Emim]^+[OAc]^-$ stoichiometry is nearly (1:1). However, in the case of 20 M and 50 M solutions, the intensity of peak B constantly increased compared to the peak-A, indicating excess TA mostly dominates these concentrations.

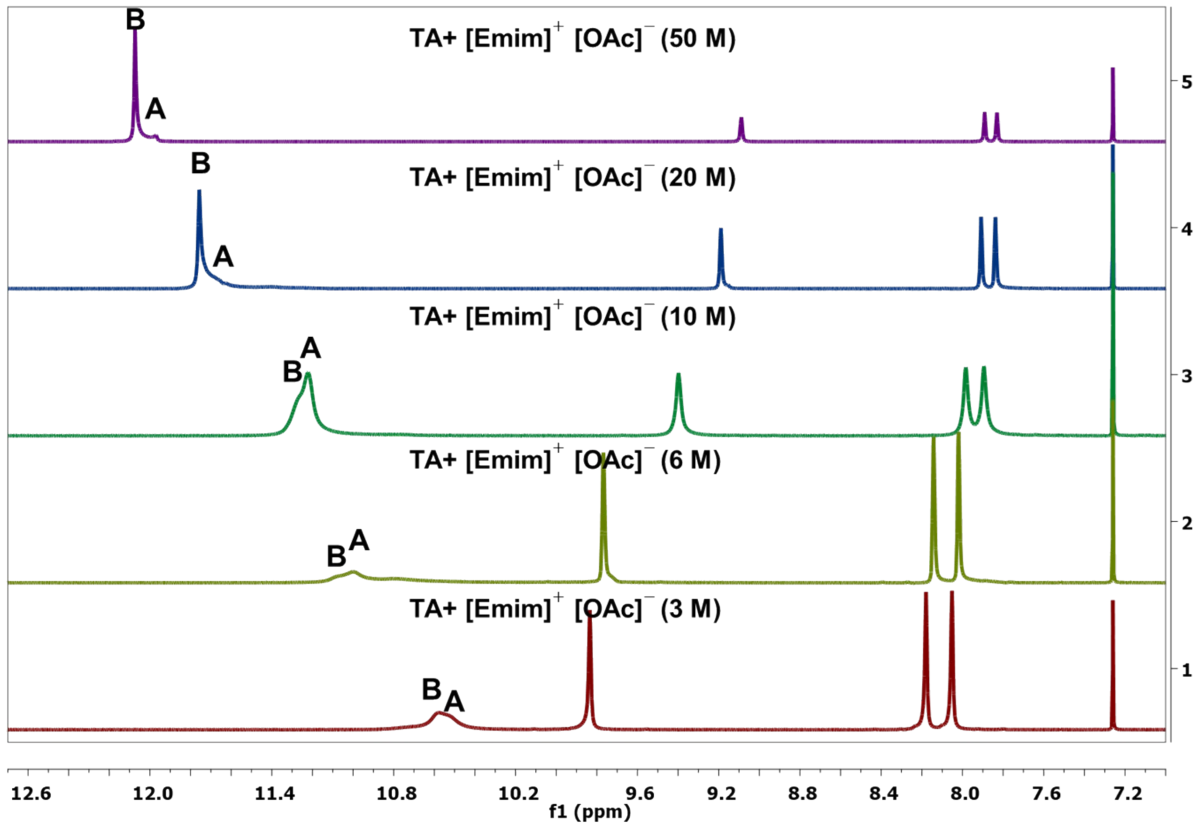

Figure 3: NMR spectra of the dilution experiments in the range between 7-12.5 ppm.

Now, if the aliphatic region is considered (0-5ppm), the peak in between 4-5ppm and 1.5-2 (triplet) corresponds to $[Emim]^+$. The peaks between 1.7 and 2.4 ppm possibly correspond to the methyl proton of the acetic acid and TA. With increasing concentration, a downfield shift was observed in both the methyl proton peaks. With increasing TA concentration, the intensity of peak C increased compared to peak-D, indicating peak-C corresponds to TA and peak-D corresponds to acetic acid. The downfield shifts in $-CH_3$ protons come from both the neutral and ionized species, and the relative intensity between peak C and D in 6 M and 10 M concentrations indicates the equilibrium between TA-$[Emim]^+[OAc]^-$-acetic acid.

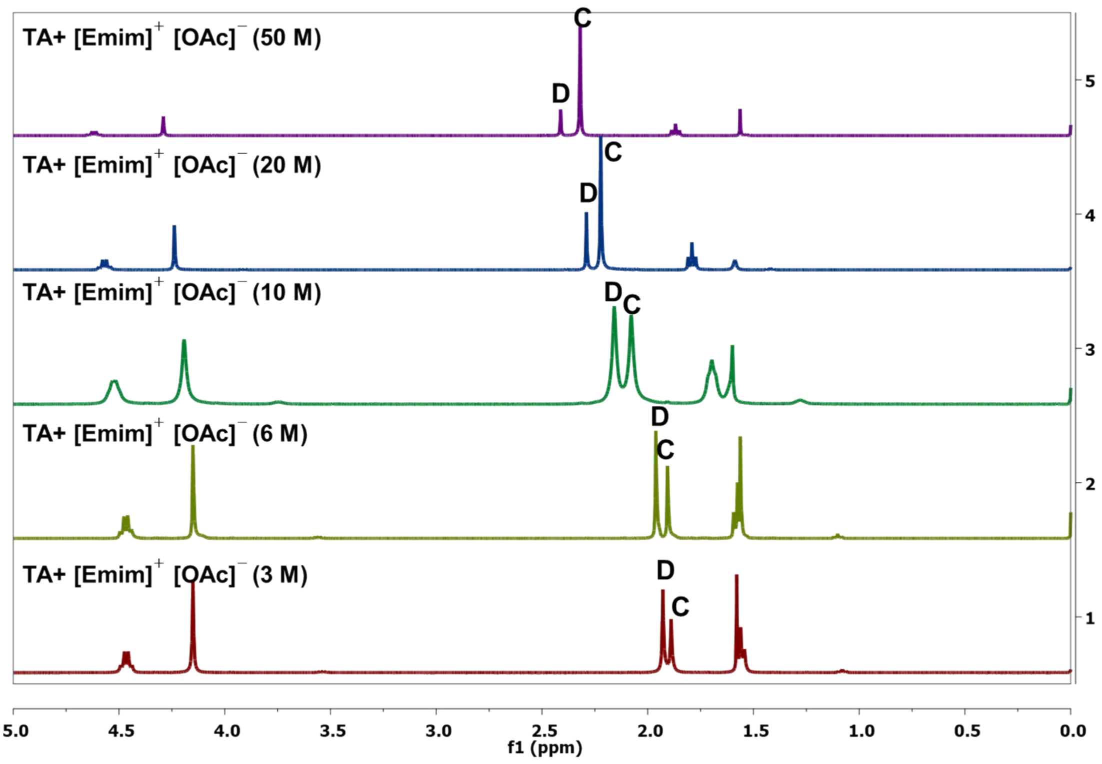

Figure 4: NMR spectra of the dilution experiments in the range between 0.0 -5.0 ppm.

(c) FT-IR spectra:

(a) ATR-FTIR data: ATR-FTIR data was collected for TA, acetic acid, $[Emim]^+[OAc]^-$ and for TA + $[Emim]^+[OAc]^-$ (6 M) solution. The C-O stretching frequency in TA, acetic acid, and [Emim]+[OAc]- appeared at 1685 cm-1, 1705 cm-1, and 1560 cm^{-1}, respectively. A peak at 1705 cm^{-1} appeared when TA was mixed with $[Emim]^+[OAc]^-$, possibly corresponding to the in-situ generated acetic acid.

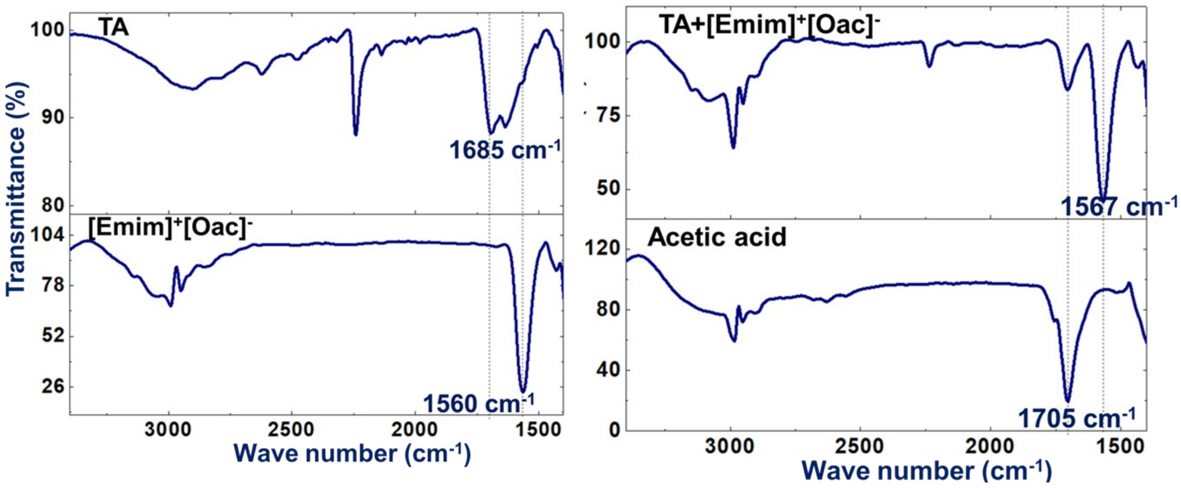

Figure 5: ATR-FTIR-Spectra of TA, $[Emim]^+$ $[OAc]^-$, TA+ $[Emim]^{+,}$ and acetic acid. (IR spectra are plotted in a range of 3800 to 1400 cm^{-1}).

(b) FTIR data (using KBr method): To further confirm the results obtained from ATR-FTIR, FTIR data was collected using the KBr method, for *Emim] $^{+}$[OAc]$^{-}$, TA+ [Emim]$^{+}$ [NTF$_2$]$^{-}$ and TA +[Emim]$^{+}$ [OAc]$^{-}$*. In the TA and [Emim]$^{+}$ [OAc]$^{-}$ mixture, a peak was observed at 1707 cm^{-1}, which is assigned as the acetic acid peak. To further confirm the peak identity, a 6 M solution of TA in [Emim]$^{+}$ [NTF$_2$]$^{-}$ was prepared. At 6 M TA [Emim]$^{+}$ [NTF$_2$]$^{-}$ a carbonyl stretching frequency appeared at 1697 cm^{-1}.

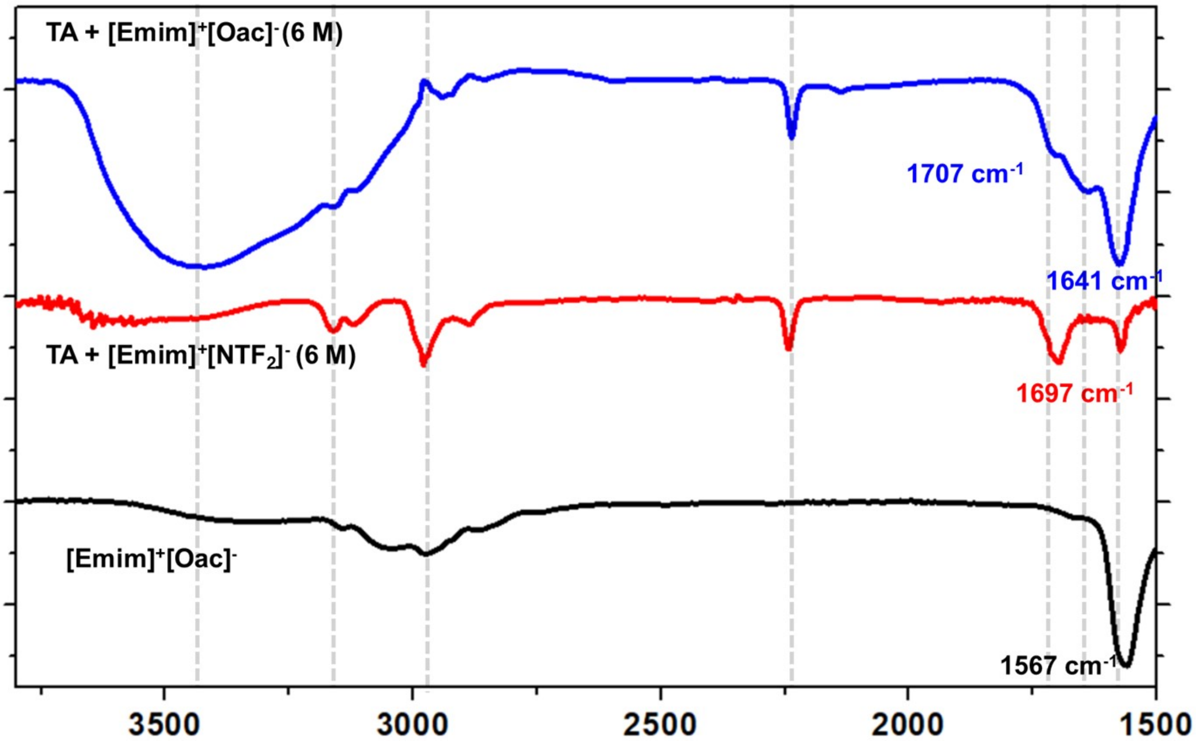

Figure 6: IR-Spectra of [Emim]$^{+}$ [OAc]$^{-}$(black), TA+ [Emim]$^{+}$ [NTF$_2$]$^{-}$(red) and TA +[Emim]$^{+}$ [OAc]$^{-}$(blue). (IR spectra are plotted in a range of 3800 to 1500 cm^{-1}).

S3. Reaction without catalyst (Blank reaction):

(a) Reaction of benzylamine and ethyl acetate without catalyst:

1 mL of ethyl acetate and 109 µl of benzylamine were taken in a 25 mL round-bottom flask (RB). The reaction mixture was stirred at 80°C for 20 hours. After that, it was cooled at room temperature, and the organic solvent was removed using a rotary evaporator. The resulting crude mixture was analyzed through ^{1}H NMR. The reactant's proton peak H$_a$ (3.82 ppm) was compared with H$_b$ (4.37 ppm), and it concluded only a 4.7% product formation.

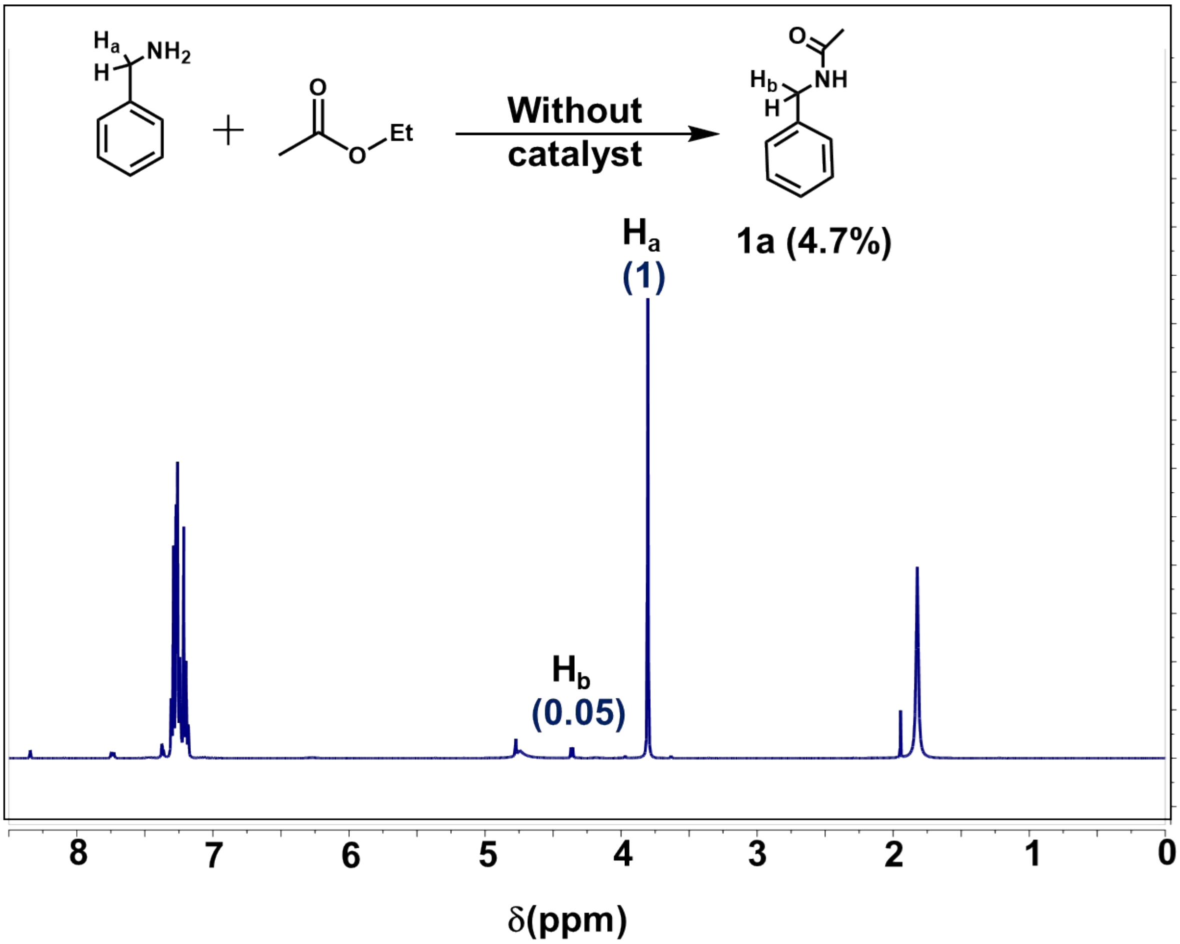

Figure 7: ^{1}H NMR of the reaction of Benzyl amine and ethyl acetate without catalyst

(b) Reaction of benzylamine and ethyl acetate in the presence of 10 mol% and 1 mol% [Emim]$^+$ [OAc]$^-$:

In a 25 mL RB 4 mL of ethyl acetate, 436 µl of benzylamine was stirred with 6 µl [Emim]$^+$ [OAc]$^-$ (1 mol%). The reaction mixture was heated at 80ºC for 20 hours. The resulting mixture was cooled and dried using a rota evaporator, and the crude mixture was analyzed using ^{1}H NMR. Comparing the reactant 3.82 ppm (corresponds to the 2Hs of benzylamine) and product peak 4.37 ppm (corresponding to 2Hs of benzyl amide), it was observed that 18% and 6% product was formed for 10 mol% and 1 mol% catalyst, respectively.

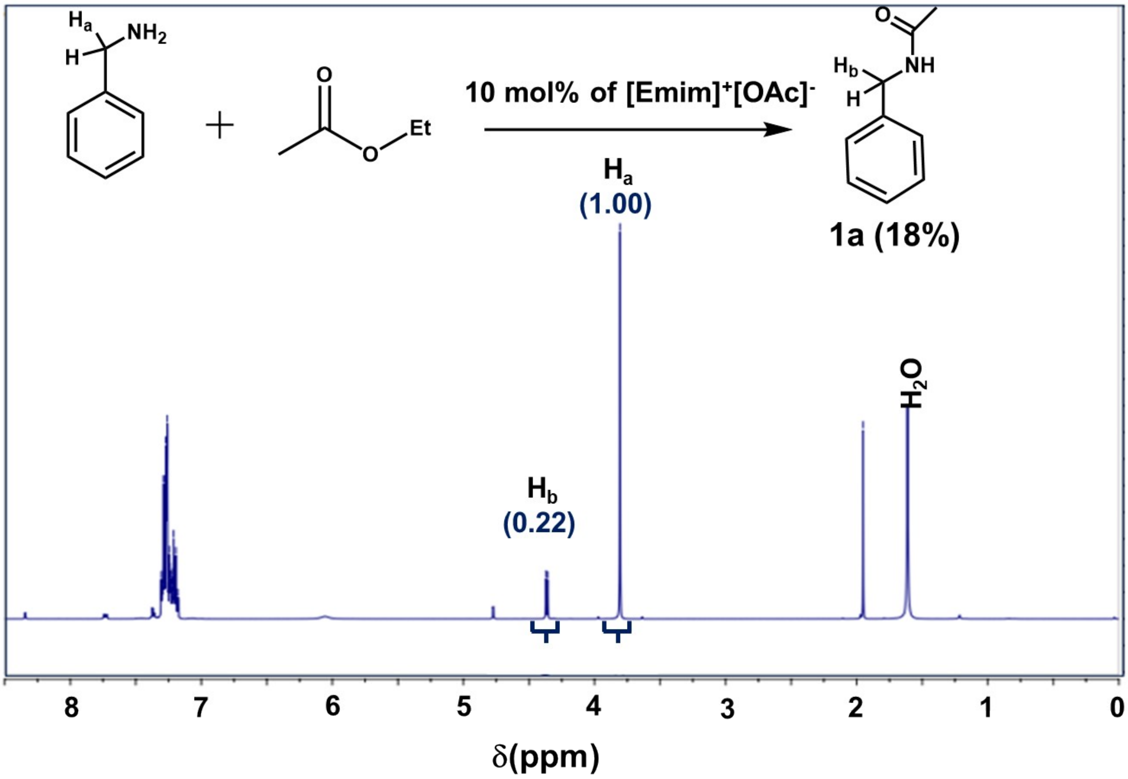

Figure 8: ^{1}H NMR of the reaction of benzylamine and ethyl acetate in the presence of 10 mol% [Emim]+ [OAc]-

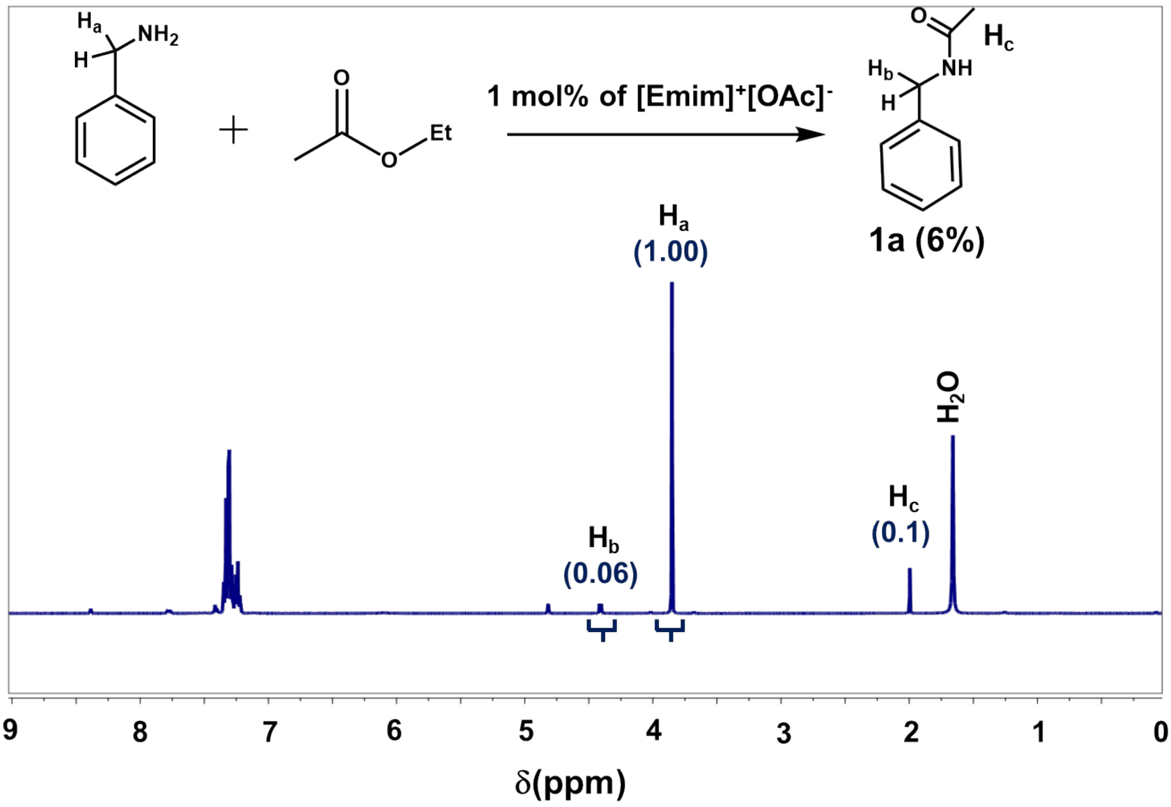

Figure 9: ^{1}H NMR of the reaction of benzylamine and ethyl acetate in the presence of 1 mol% [Emim]$^+$ [OAc]$^-$:

(c) The reaction of benzylamine and ethyl acetate using 10 mol% (TA):

1 mL of ethyl acetate and 109 µl benzylamine were mixed with 8.4 mg TA (for 10 mol%) and stirred at 80ºC for 20 hours. The product obtained was analyzed through ^{1}H NMR. Equating the peak integrals at 3.82 ppm (representing 2Hs of benzylamine) and 4.37 ppm (corresponding to 2Hs of benzyl amide), it was observed that 27% and 20% product conversion occurred for 10 mol% and 1 mol% of TA.

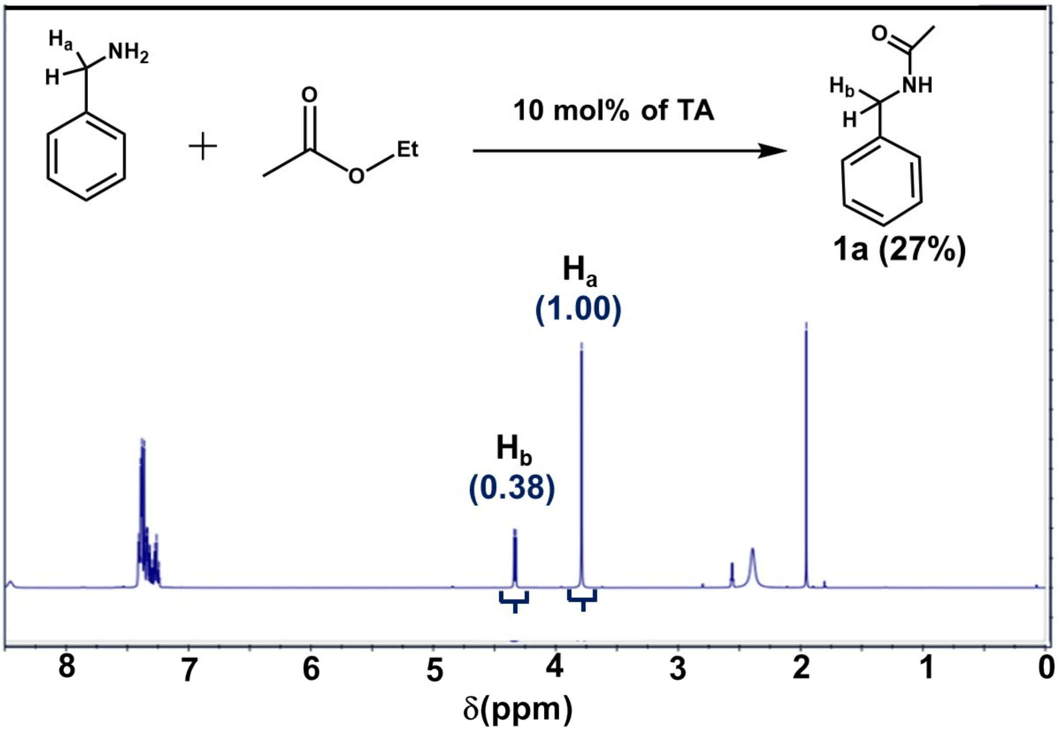

Figure 10: ^{1}H NMR of the reaction of benzylamine and ethyl acetate using 10 mol% (TA):

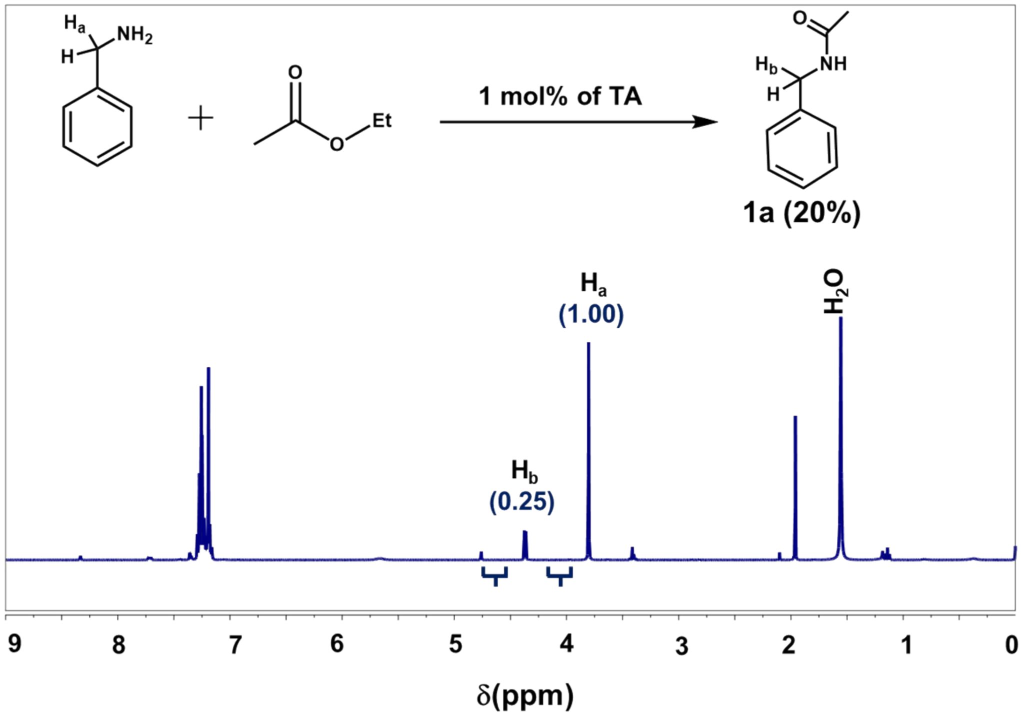

Figure 11: ^{1}H NMR of the reaction of benzylamine and ethyl acetate using 1 mol% (TA):

S4. Reaction with Catalyst:

(a) Reaction time optimization with 10 mol% catalysts: reaction yield of benzylamine and ethyl acetate using TA (10 mol%) and [Emim]⁺ [OAc]⁻ (10 mol%) after 4, 8, 12, 16, and 20 hours:

The reactants 1 mL of ethyl acetate and 109 µl of benzylamine were taken in a 50 mL RB along with 10 mol% of TA and $[Emim]^+$ $[OAc]^-$. This mixture was heated at 80ºC, and aliquots of the reaction were collected every four hours intervals and dried under a vacuum. The product obtained was dissolved in $CDCl_3$, and NMR data was collected. A stacked NMR Plot with different time intervals is shown below. With time, a decrease in reactant peak Ha (3.82 ppm corresponds to 2Hs of benzylamine) and an increase in product peak Hb (4.37 ppm corresponding to 2Hs of benzyl amide) was observed. A 100% conversion was observed after 20 hours of stirring under 80ºC.

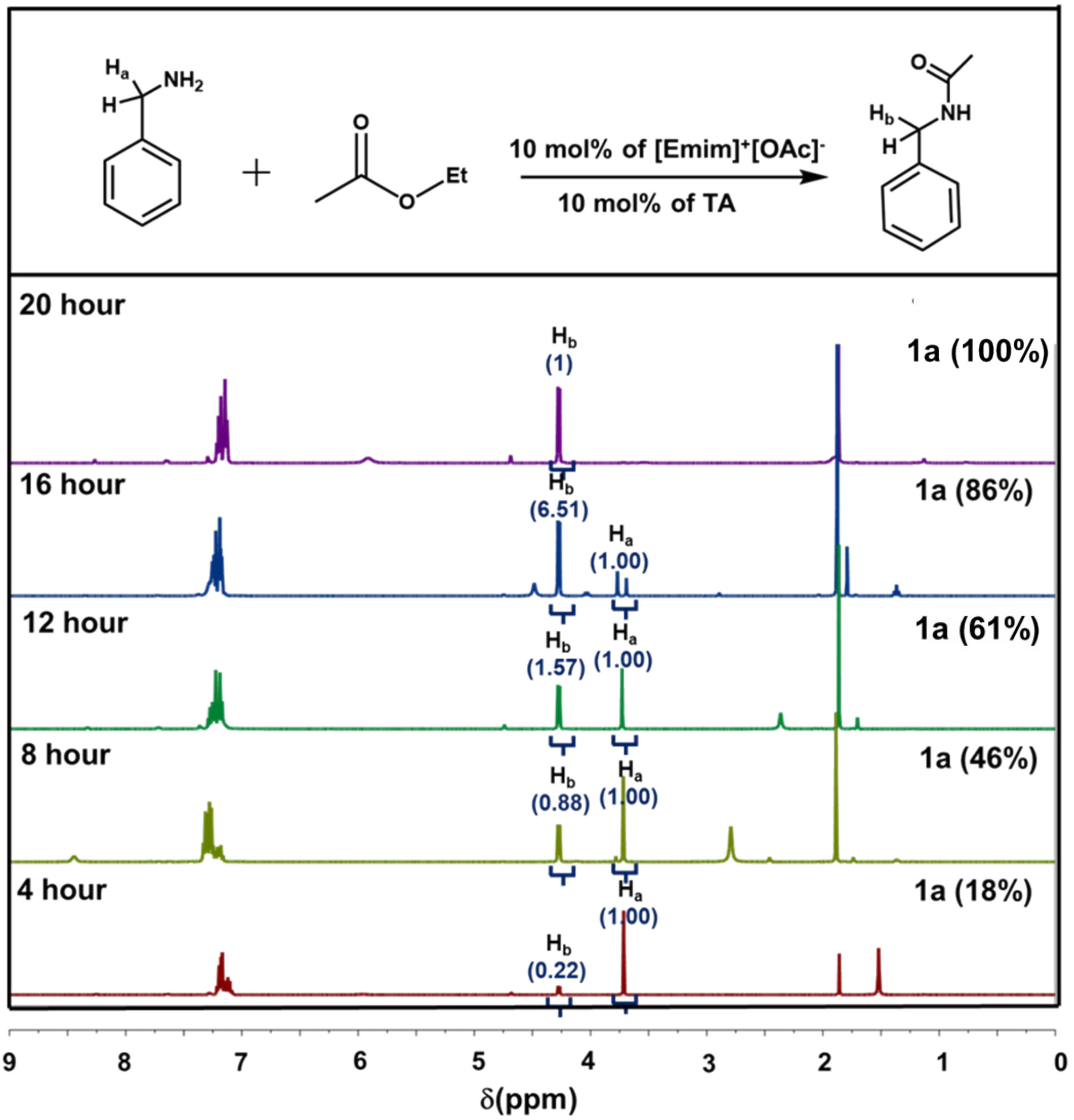

Figure 12: ^{1}H NMR of the reaction of benzylamine and ethyl acetate using 10 mol% catalysts and optimization of reaction with time.

(b) *Catalytic load optimization at constant time:*

We obtained a 100% product (1a) in a previous reaction using a 10 mol% catalyst in 20 hours. Another reaction was performed by taking 4 mL ethyl acetate,436 µl benzylamine, 3.3 mg TA, and 6 µl $[Emim]^+$ $[OAc]^-$ (1 mol% catalyst). The resultant mixture was then heated at 80ºC for 20 hours. After the reaction mixture was cooled at room temperature, the organic solvent was removed using a rotary evaporator. No reactant peak (H_a) remained at 3.82 ppm, which confirmed a 100% conversion occurred.

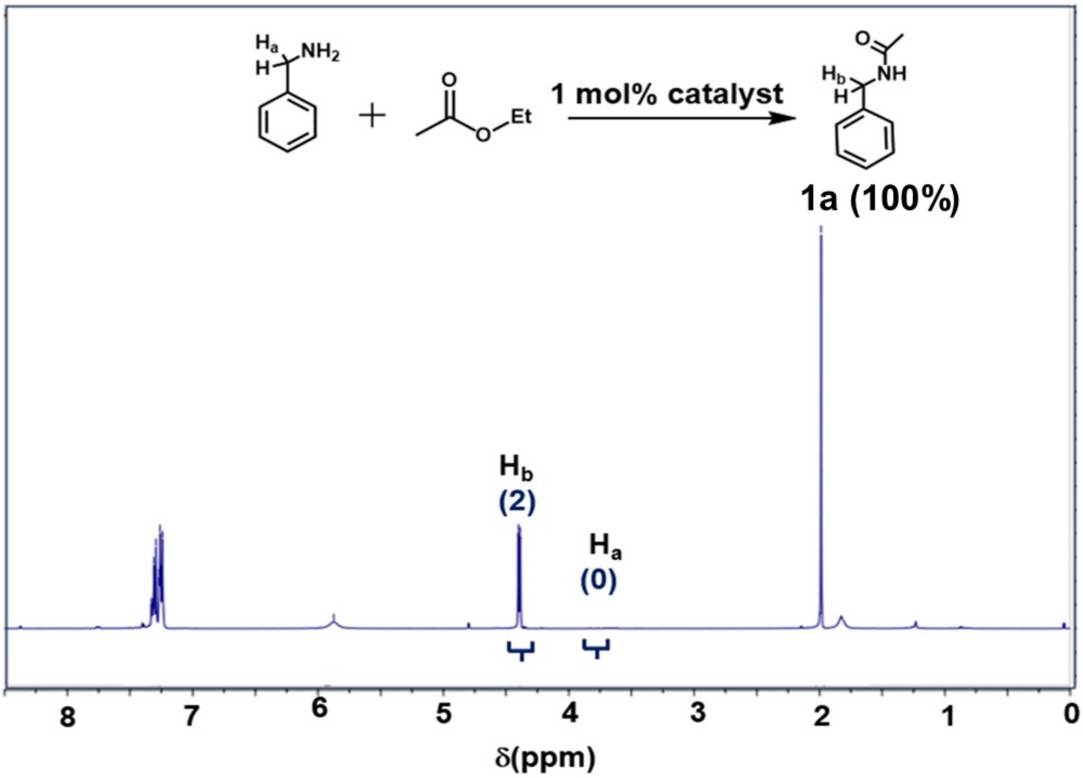

Figure 13: ^{1}H NMR of reaction of benzylamine and ethyl acetate using TA (1mol%) and $[Emim]^+$ $[OAc]^-$ (1 mol%)

S5. Reaction of Aliphatic Amines:

(a) Reaction of 4-bromobenzylamine and ethyl acetate using TA (1 mol%) and $[Emim]^+$ $[OAc]^-$ (1 mol%):

4-bromobenzyl amine (740 mg) was weighed and mixed with 4 mL of ethyl acetate in a 50 mL round-bottom flask, followed by the addition of the mixture of 3.3 mg TA and 6 µl $[Emim]^+$

[OAc]⁻, a 1 mol% catalyst mixture. The resultant mixture was stirred at 80ºC for 20 hours. After that, the product obtained was dissolved in $CDCl_3$, and NMR data was collected. When the reactant's proton peak (H_a) was integrated with the product's proton peak at 4.4 ppm (H_b), a 90% conversion was found.

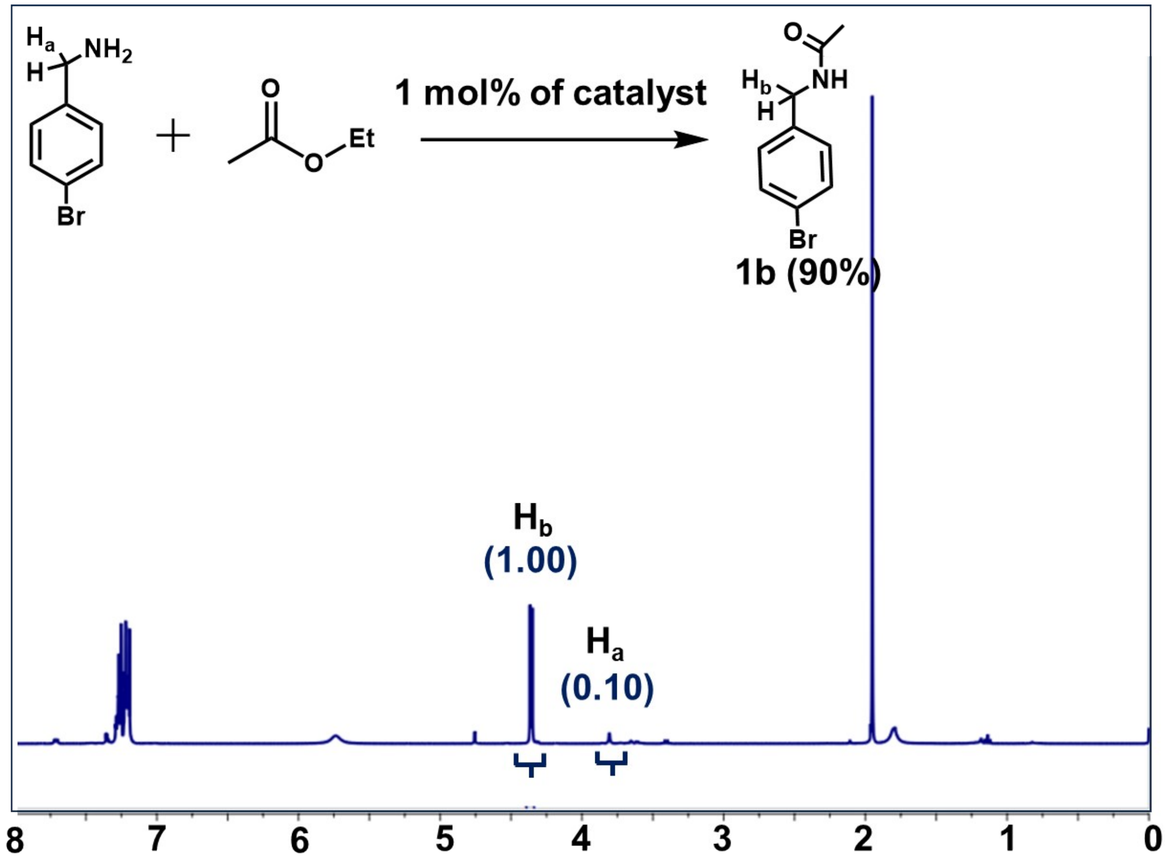

Figure 14: ¹H NMR of the reaction of 4-bromobenzylamine and ethyl acetate using TA (1 mol%) and [Emim]⁺ [OAc]⁻ (1 mol%):

(b) *The reaction of 4-(trifluoromethyl) benzylamine and ethyl acetate using (TA) (1 mol%) and [Emim]⁺ [OAc]⁻ (1 mol%):*

Highly deactivating 4-(trifluoromethyl) benzylamine (700 µl) was taken in an RB with 4 mL of ethyl acetate, 1 mol% catalytic mixture of TA (3.3 mg), and [Emim]⁺ [OAc]⁻ (6 µl). The mixture was stirred at 80ºC for 20 hours. The obtained product was analyzed through NMR. Integrating the product (H_b)(int=0.71) and reactant peak (H_a) (int=1), The product conversion calculated was $\frac{0.71}{1.71} \times 100 =$ 41.5%.

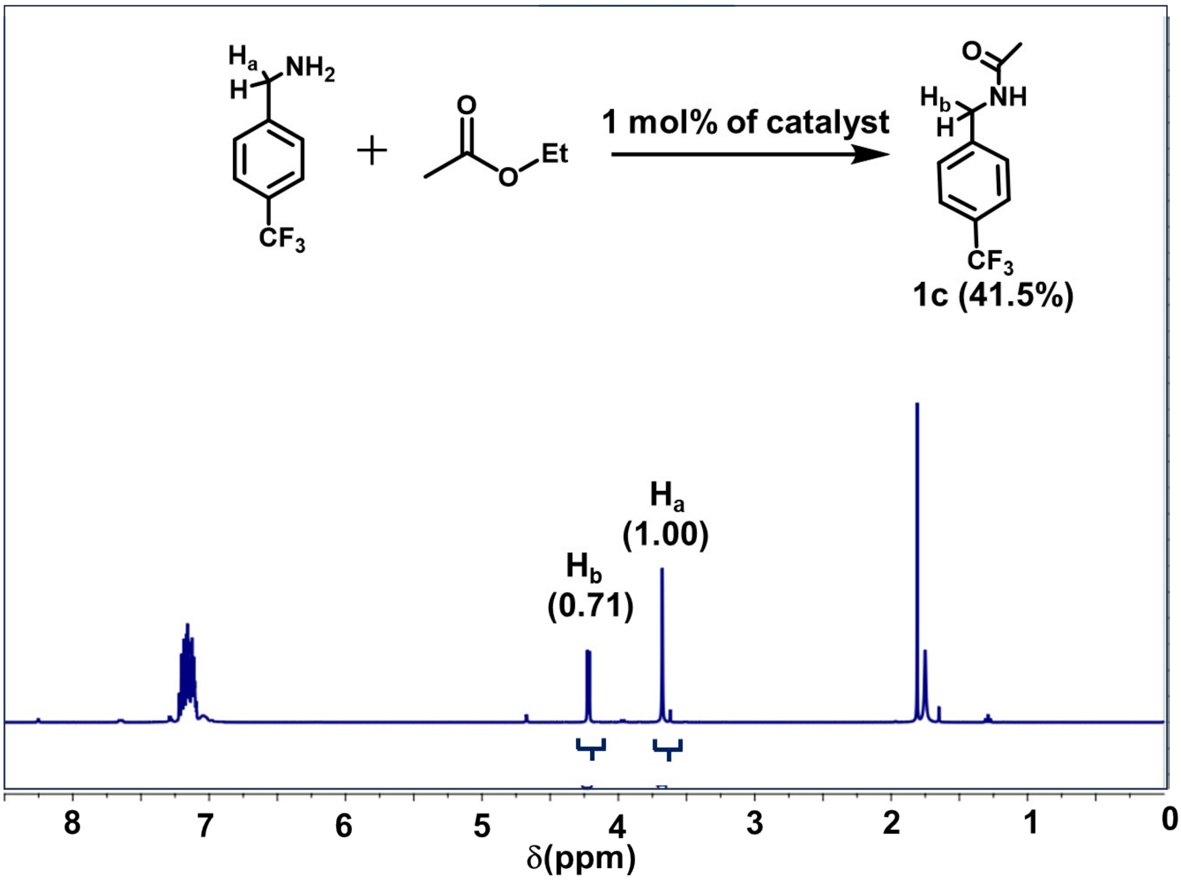

Figure 15: ^{1}H NMR of the reaction of 4-(trifluoromethyl) benzylamine and ethyl acetate using (TA) (1 mol%) and [Emim]$^+$ [OAc]$^-$ (1 mol%):

(c) (i) Reaction of ethanolamine and ethyl acetate using TA (1 mol%) and [Emim]$^+$ [OAc]$^-$ (1 mol%):

To check the chemoselectivity of the reaction, a bifunctional molecule (containing both hydroxy and amine functionality), ethanolamine (244 µl), was chosen, and the reaction was performed with 4 mL of ethyl acetate. 1 mol% of catalyst (3.3 mg TA and 6 µl [Emim]$^+$ [OAc]$^-$) was added to the reaction mixture. The reaction was continued for 20 hours at 80 ºC. The appearance of the H_c peak at 1.3 ppm confirmed the product formation. Other integration of other protons (H_a and H_b) also corresponds with the methyl proton's (H_c) integration. This result hinted at a 100% product formation. To further confirm the reaction's chemo selectivity, IR data was collected with the product.

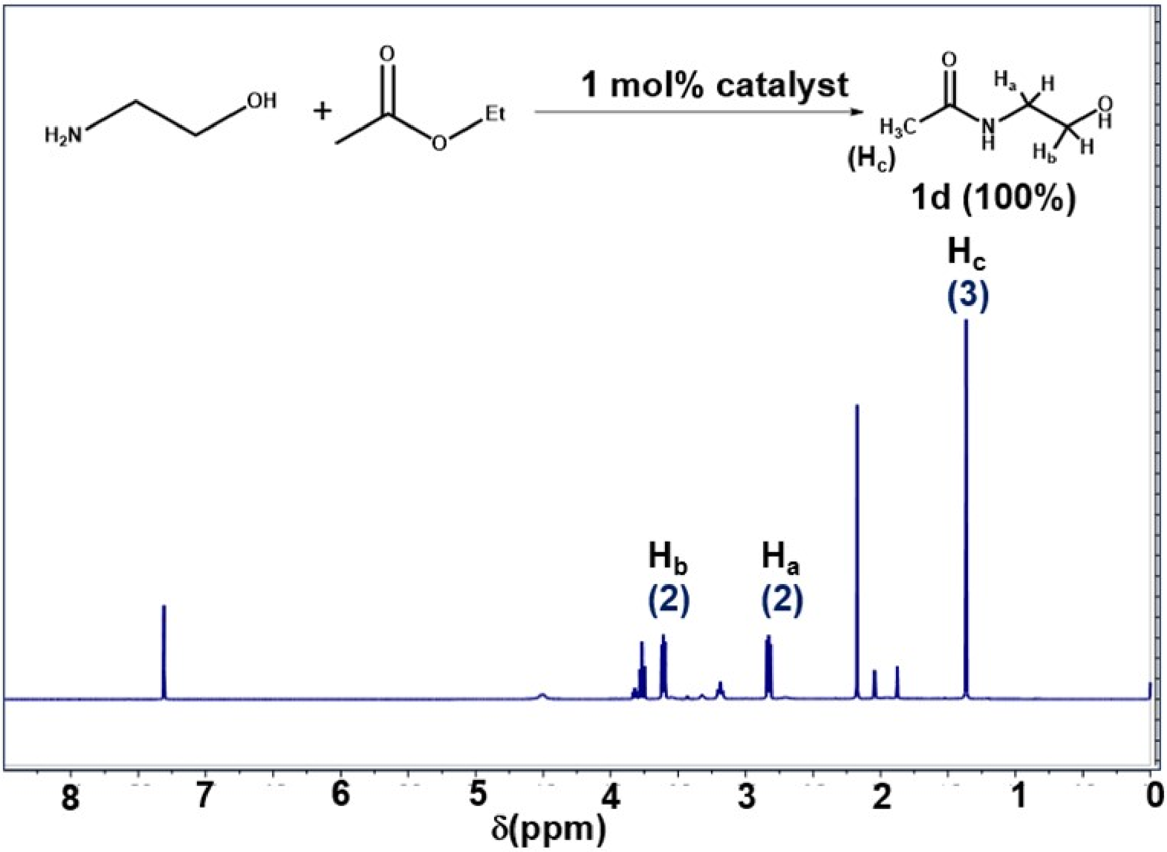

Figure 16: ^{1}H NMR of the reaction of ethanolamine and ethyl acetate using TA (1 mol%) and [Emim]$^{+}$ [OAc]$^{-}$ (1 mol%):

(ii) Confirmation of amide bond formation in N-(2-hydroxyethyl) acetamide through IR: ATR-FTIR data was collected to confirm the chemoselectivity of the approach. Around 10 mg of the product obtained through the reaction was ground, and IR-spectra was collected using Bruker alpha ATR-FTIR instrument. Generally, the carbonyl stretching peak in ester appeared around 1735-1750 cm^{-1}, and the carbonyl stretching frequency for amide appeared in a range between 1630-1680 cm^{-1}. In the subsequent FTIR spectra, no peak appeared above 1650 cm^{-1} (up to 2500 cm^{-1}); this confirmed no ester formed with the hydroxyl group. The peak at 1633 cm^{-1} confirmed the amide formation. This proves the chemoselectivity of the reaction.

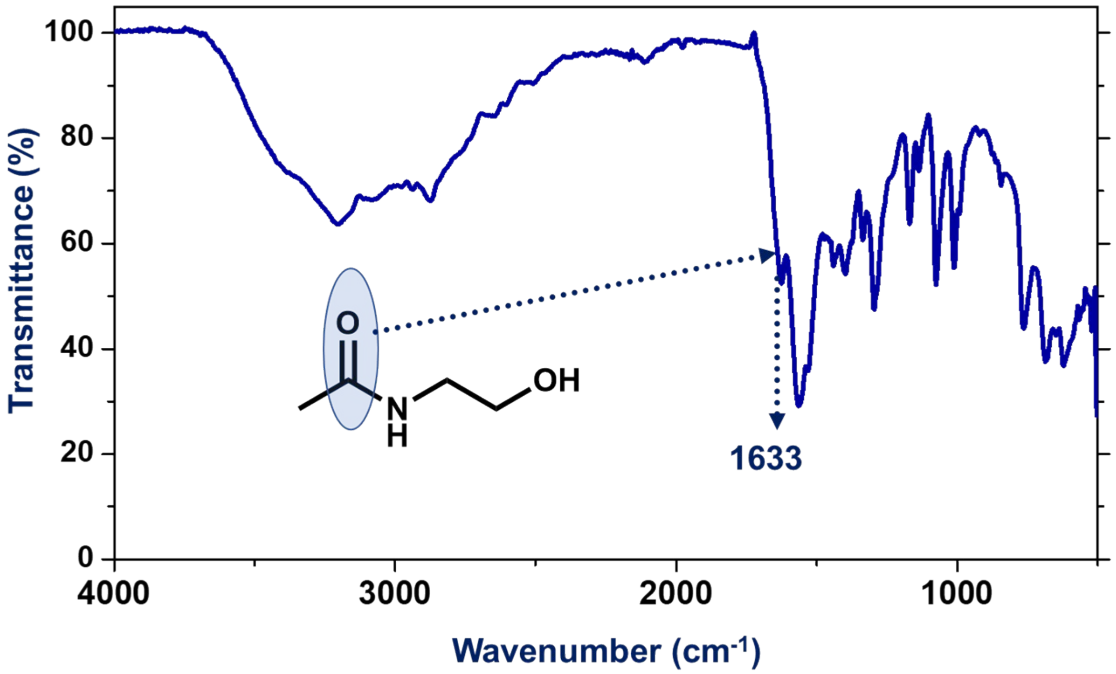

Figure 17: FT-IR spectra of *the reaction of ethanolamine and ethyl acetate using TA (1 mol%) and [Emim]$^+$ [OAc]$^-$ (1 mol%):*

(d) Reaction of N-methyl-1-phenylmethanamine and butyl acetate using (TA) (1 mol%) and [Emim]$^+$ [OAc]$^-$ (1 mol%):

For secondary amines, harsher conditions are needed; that's why ethyl acetate was replaced with higher boiling butyl acetate (b.p=126 ºC). In a 50 mL RB, 4 mL of butyl acetate and 484 µl of N-methyl-1-phenylmethanamine were added through weighing, followed by the addition of 1 mol% catalyst (the mixture of 3.3 mg TA and 6 µl [Emim]$^+$ [OAc]$^-$). The mixture was stirred vigorously at 115ºC for 24 hours. The crude product obtained was dried under vacuum and analysed through NMR. The ^{1}H NMR peaks at δ=7.38 – 7.15 (m, 5H, Ph) is for the aromatic protons, δ=4.57 4.51 (d, 2H) appeared due to H_a proton, δ= 2.93-2.91 (d, 3H) for H_b, δ=2.14-2.13 (d, 3H) for H_c proton. The three peaks should appear as a singlet; however, due to major and minor rotamer formation, the peaks split and appeared as a doublet. Correlating all the proton peaks and the absence of reactant peaks confirmed a 100% product formation.

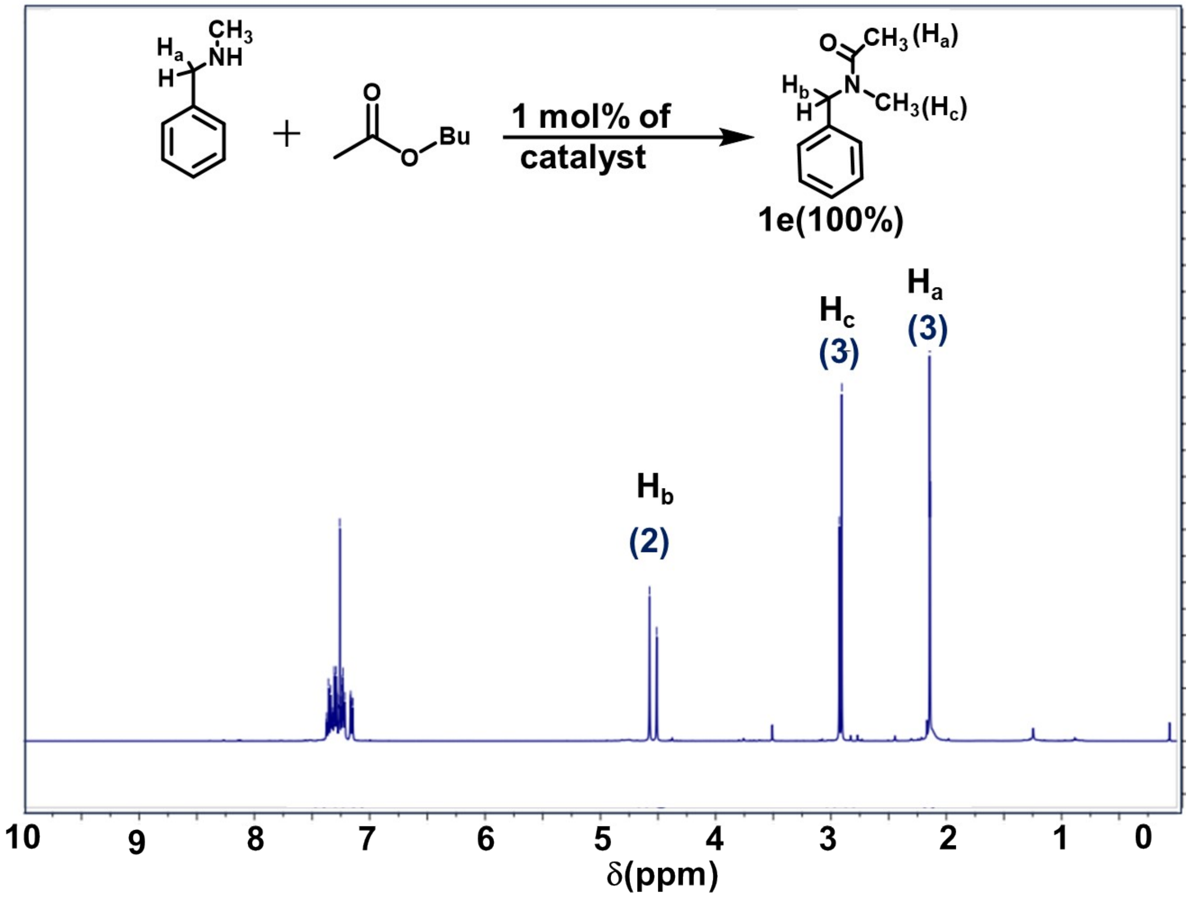

Figure 18: ^{1}H NMR of the reaction of N-methyl-1-phenylmethanamine and butyl acetate using (TA) (1 mol%) and [Emim]$^+$ [OAc]$^-$ (1 mol%):

S6. Reaction of Aromatic Amines:

(a) Reaction of aniline and butyl acetate using (TA) (1 mol%) and [Emim]$^+$ [OAc]$^-$ (1 mol%):

Aromatic amines were also deactivated compared to primary amines, so butyl acetate was used for all aromatic amines, replacing ethyl acetate for harsher conditions. The reaction was performed with 4 mL of butyl acetate and 372 µl of aniline in a 50 mL round-bottom flask, followed by adding the mixture of 3.3 mg TA and 6 µl [Emim]$^+$ [OAc]$^-$. The reaction was performed at 115ºC for 24 hours. After completion, the mixture was cooled at room temperature; the organic solvent was removed using a rotary evaporator. Then the resulting crude mixture was analysed via ^{1}H NMR. The appearance of the Peak at 2.1 ppm (H_d) confirmed the amide formation; when it was integrated as 3H, the integration for protons in the aromatic region came as 5. These suggested a 100% product formation.

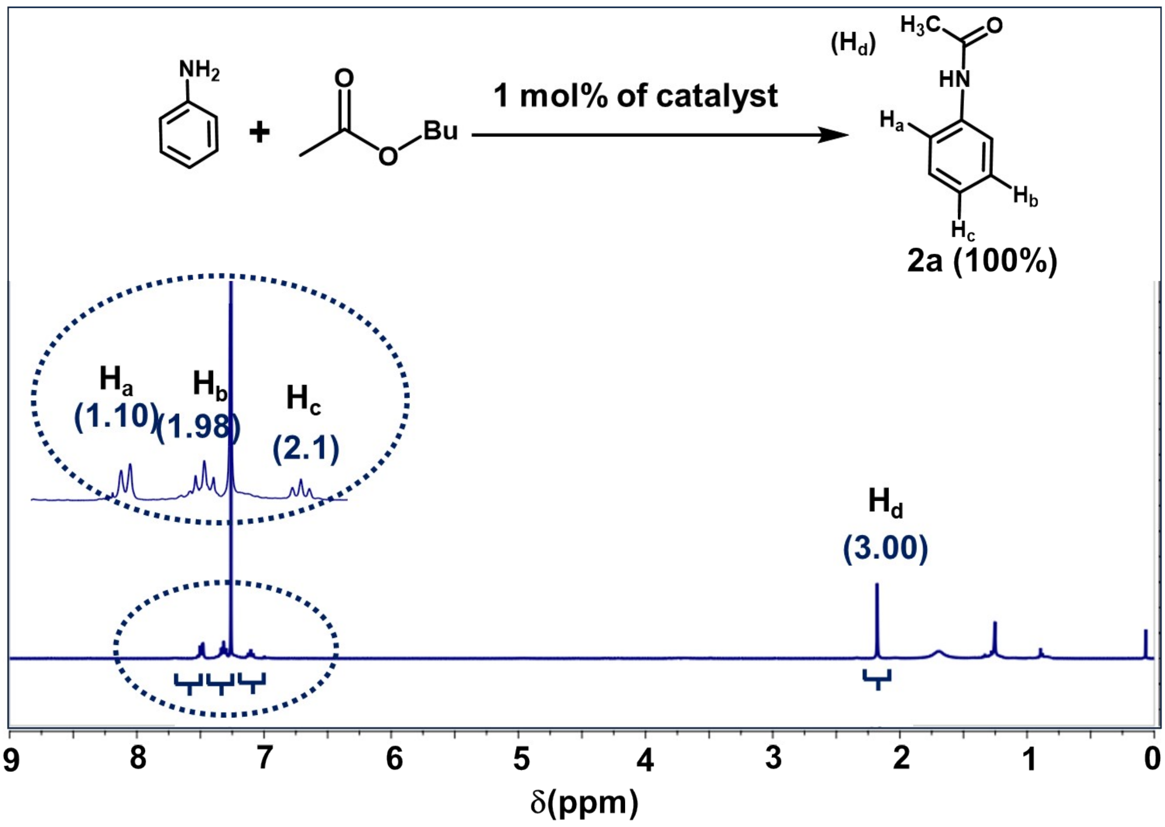

Figure 19: ^{1}H NMR of the reaction between aniline and butyl acetate using (TA) (1 mol%) and [Emim]$^+$ [OAc]$^-$ (1 mol%)

(b) Reaction of p-toluidine and butyl acetate using (TA) (1 mol%) and [Emim]$^+$ [OAc]$^-$ (1 mol%):

In the experimental procedure, a mixture of 4 mL of butyl acetate and 428 µl of p-toluidine was taken in a 50 mL round-bottom flask, followed by the addition of the mixture of 3.3 mg TA and 6 µl [Emim]$^+$ [OAc]$^-$. This mixture was then heated at a temperature of 115ºC for 24 hours. After the reaction mixture was cooled at room temperature, the organic solvent was removed using a rotary evaporator. The NMR data suggested the absence of any reactant peak and integration of all product proton matching with the numbers of proton in the product; this concluded the formation of 100% product.

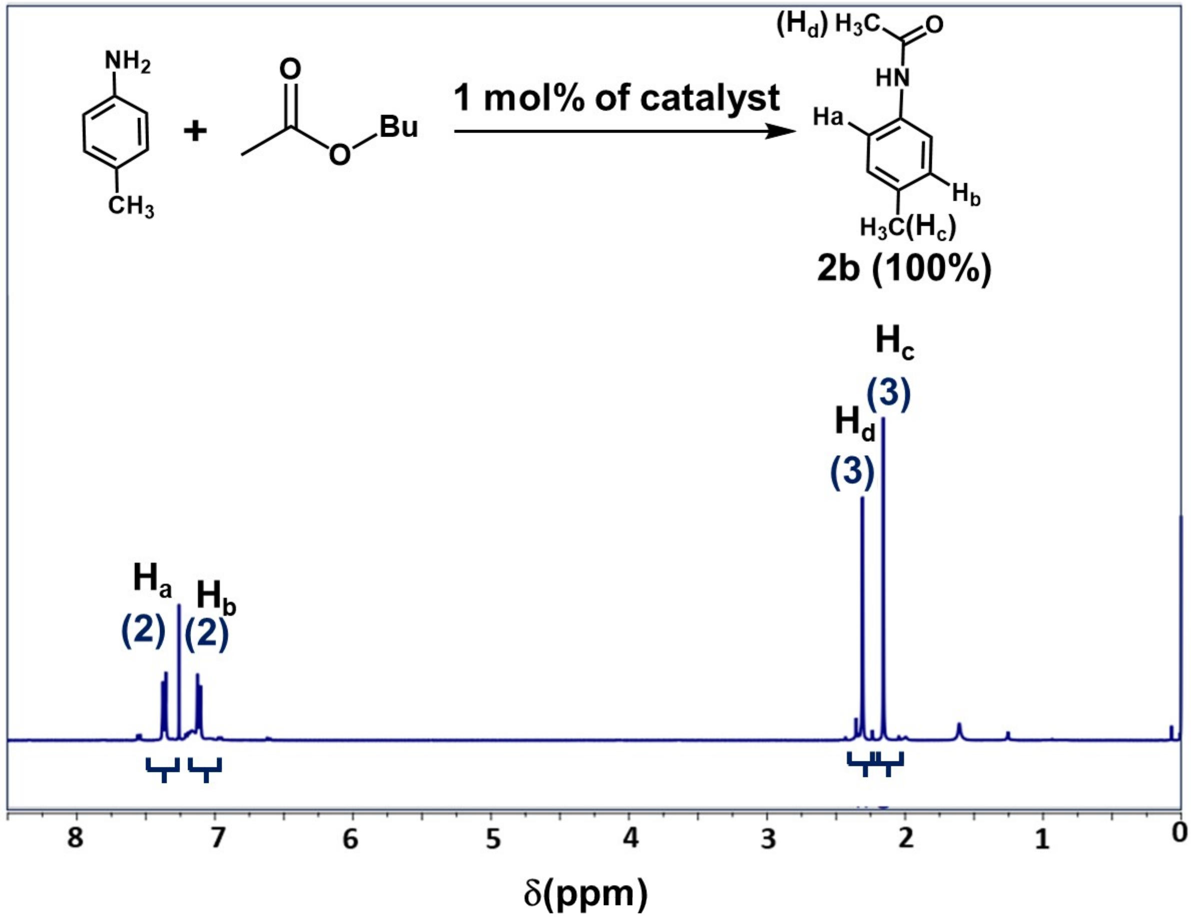

Figure 20: ^{1}*H NMR of the reaction between* p-toluidine and butyl acetate using (TA) (1 mol%) and $[Emim]^+$ $[OAc]^-$ (1 mol%)

(c) Reaction of p-anisidine and butyl acetate using (TA) (1 mol%) and $[Emim]^+$ $[OAc]^-$ (1 mol%):

In the experimental procedure, a mixture of Activated aromatic amine p-anisidine (492 µl) and 4 mL of butyl acetate was weighed and transferred in a 50 mL RB. 1 mol% catalyst (3.3 mg TA and 6 µl $[Emim]^+$ $[OAc]^-$) was added into it. This mixture was then heated at 115ºC for 24 hours. After 24 hours of stirring, the mixture was cooled at room temperature and dried. The appearance of the H_c proton peak at 2.1 confirmed the amide formation. When amide proton (H_c) was integrated as 3, all other proton integration matched the product proton numbers (Ha=2, Hb=2, Hd=3), confirming a 100% conversion. All the product proton peaks are assigned below.

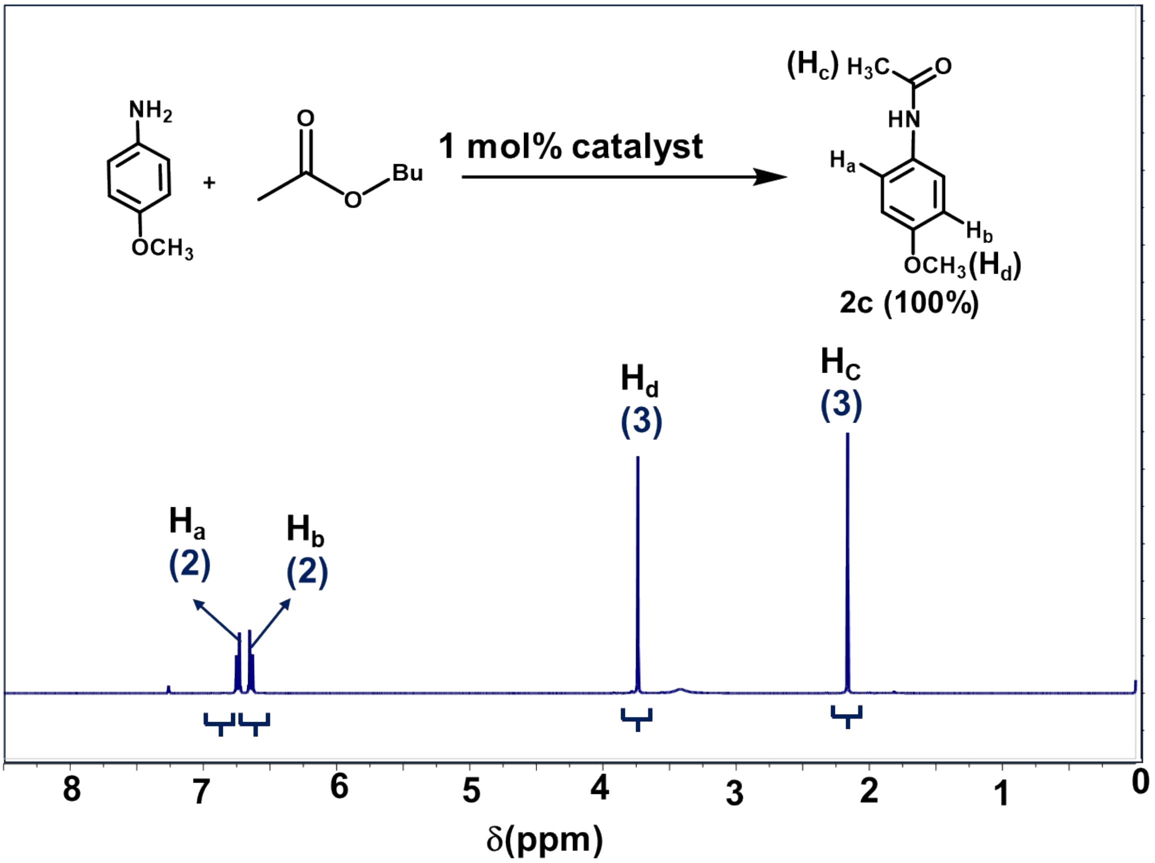

Figure 21: ¹H NMR of the reaction between p-anisidine and butyl acetate using (TA) (1 mol%) and $[Emim]^+$ $[OAc]^-$ (1 mol%):

(d) Reaction of phenylenediamine and butyl acetate using (TA) (1 mol%) and $[Emim]^+[OAc]^-$ (1 mol%):

The experimental procedure involved taking a mixture of 4 mL of butyl acetate and 432 mg of phenylenediamine in a 25 mL round-bottom flask. Next, a mixture of 3.3 mg of TA and 6 µl of $[Emim]^+$ $[OAc]^-$ was added to the flask. The mixture was then heated at 115ºC for 24 hours. After cooling the reaction mixture to room temperature, the organic solvent was removed using a rotary evaporator. The resulting crude mixture was then analyzed using ¹H NMR. The resulting NMR peaked at 2.1 ppm, for H_d proton, which confirmed the product formation. If the acylation happened in both the amines, the integration of H_a and H_b should come as 2, when 6 methyl protons were integrated as 6. When the methyl protons were integrated as 3, the Integration of H_a and H_b protons appeared as ~2. This concludes the formation of a 100% mono-acylated product.

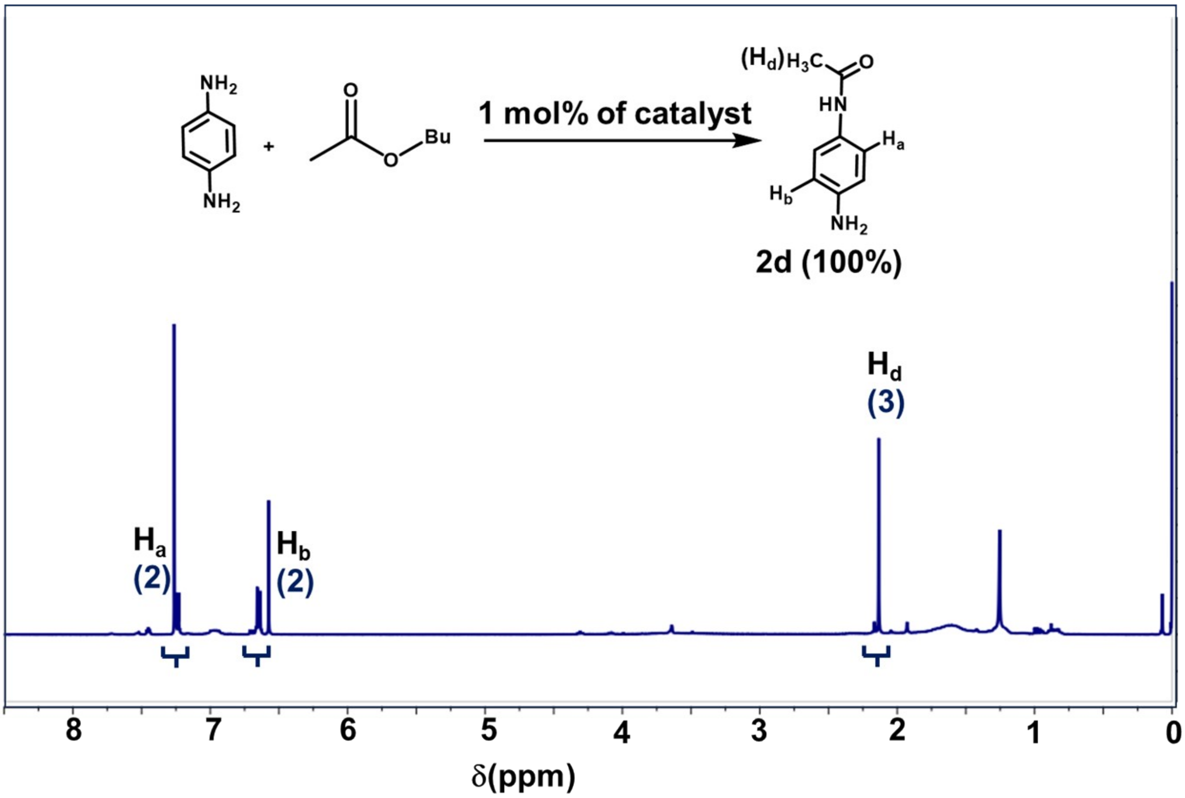

Figure 22: ¹H NMR of the reaction between phenylenediamine and butyl acetate using (TA) (1 mol%) and $[Emim]^+[OAc]^-$ (1 mol%)

(e) Reaction of 1-napthylamine and butyl acetate using (TA)(1 mol%) and $[Emim]^+[OAc]^-$ (1 mol%):

In the experiment, a 25 mL round-bottom flask was used by taking 4 mL of butyl acetate and 572 mg of 1-naphthylamine, followed by adding 3.3 mg TA and 6 µL $[Emim]^+$ $[OAc]^-$. This mixture was heated at 115ºC for 24 hours. After cooling to room temperature, the organic solvent was removed with a rotary evaporator. The resulting crude mixture was then analyzed using ^{1}H NMR. The appearance of a peak at 2.2 ppm (H_e) confirmed the formation of the product. When the H_e proton was integrated as 3, the 7 protons appeared at the aromatic region. This confirmed a 100% product formation.

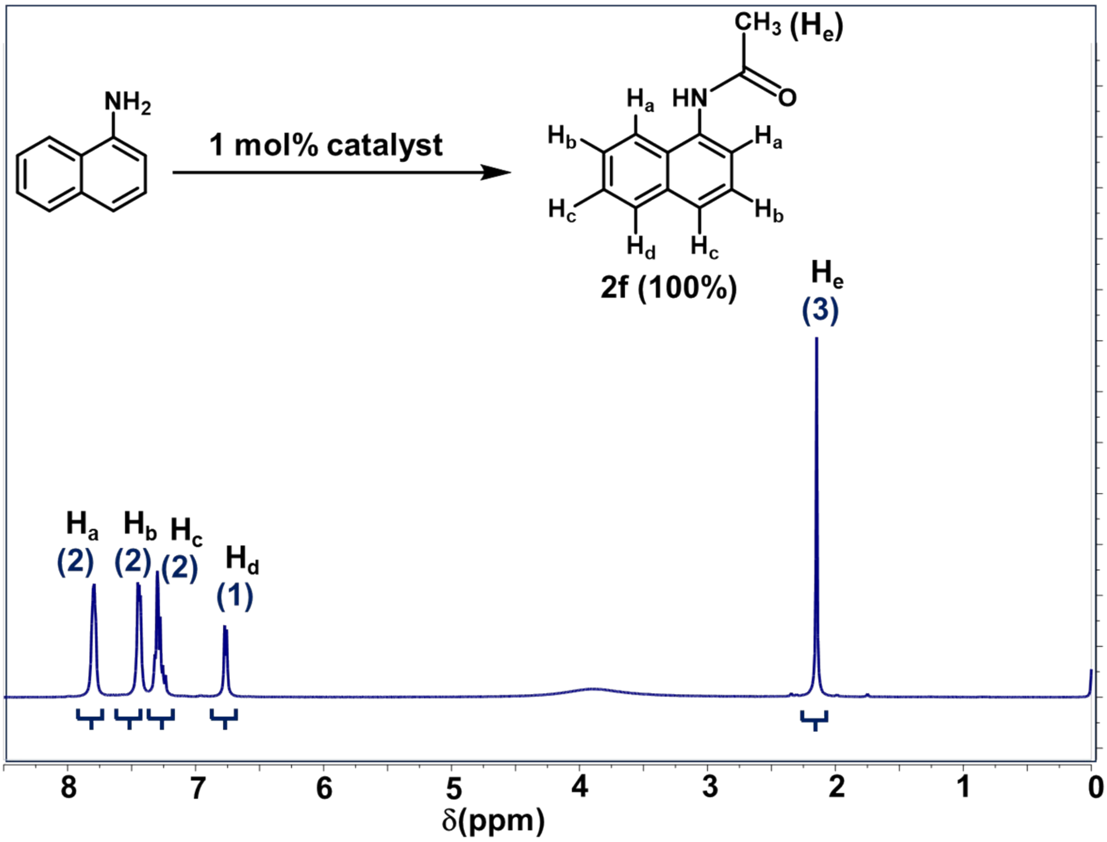

Figure 23: ^{1}H NMR of the reaction between 1-napthylamine and butyl acetate using (TA) (1 mol%) and [Emim]+[OAc]- (1 mol%)

(f) Reaction of 4-ethoxyaniline and butyl acetate using (TA) (1 mol%) and $[Emim]^+[OAc]^-$ (1 mol%):

4 mL of butyl acetate and 548 mg of 4-ethoxy aniline were taken in a round-bottom flask. To this mixture, 3.3 mg of a catalyst (TA) and 6 µl of ionic liquid ($[Emim]^+$ $[OAc]^-$) were added. The flask was then heated to 115ºC and maintained at this temperature for 24 hours. After cooling to room temperature, the organic solvent was removed using a rotary evaporator. The remaining crude product was then analyzed using ^{1}H NMR spectroscopy. The integration of the proton (H_e) was assigned as 3, and all the other proton integration matched with the expected product proton numbers (H_a=2, H_b=2, H_d=2, and H_c=3), confirming a 100% conversion.

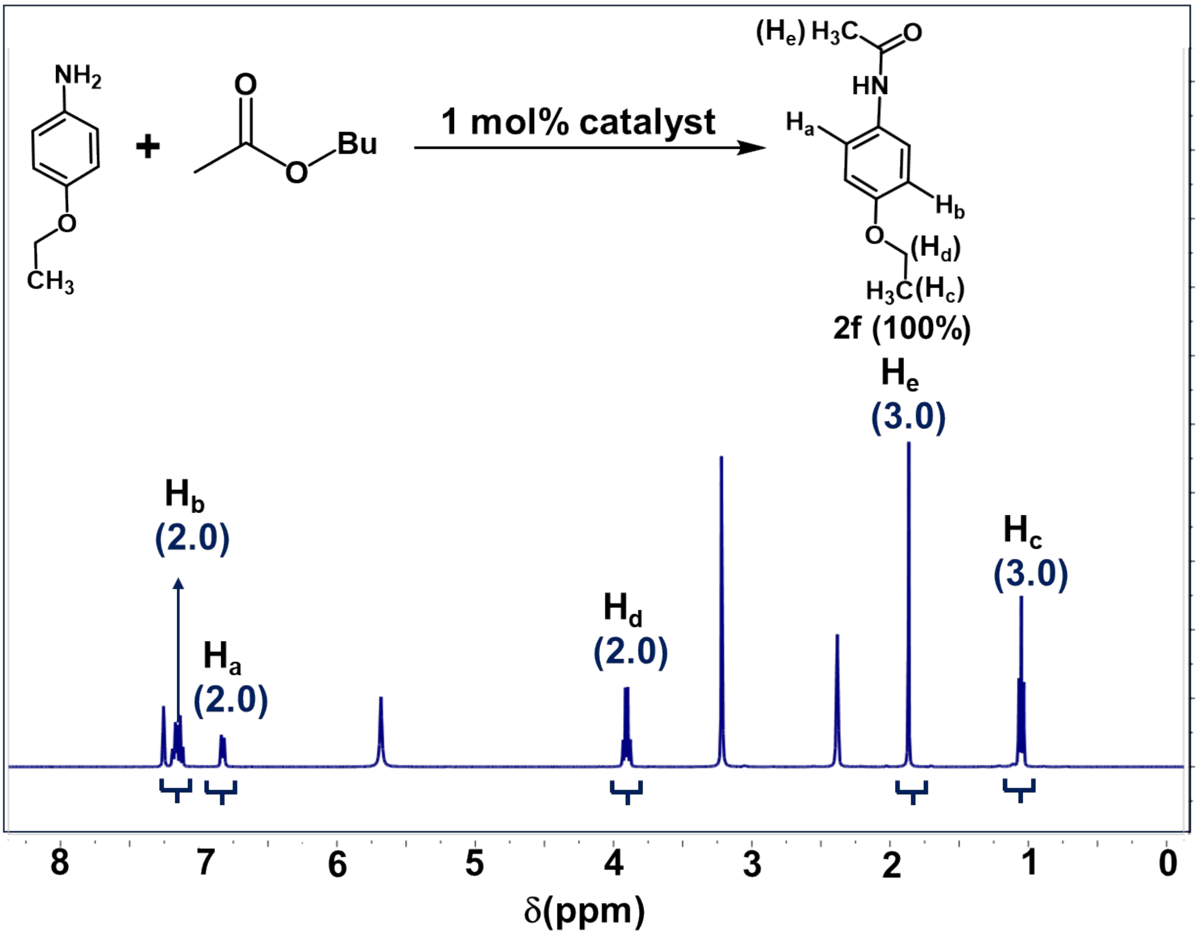

Figure 24: ^{1}H NMR of the reaction between 4-ethoxy aniline and butyl acetate using (TA) (1 mol%) and [Emim]$^+$[OAc]$^-$ (1 mol%)

(g) Reaction of 4-aminopyridine and butyl acetate using (TA) (1 mol%) and [Emim]$^+$ [OAc]$^-$ (1 mol%):

4 mL of butyl acetate and 376 µl of 4-aminopyridine were taken in a 25 mL round-bottom flask. This added 3.3 mg TA and 6 µl [Emim]$^+$ [OAc]$^-$. This mixture was then heated at a temperature of 115ºC for 24 hours. After the reaction mixture was cooled at room temperature, a rotary evaporator was used to remove the solvent. Then the resulting crude mixture was analysed via ^{1}H NMR. The appearance of the proton peak at 2.1 for H_c confirmed the product formation. For 100% conversion, if H_c was integrated as 3, H_a or H_b should come as 2. However, when the aromatic region peaks were integrated (H_a and H_b), the resultant integration was 4.7. The product formed in this reaction was $\frac{ideal\ product\ integration}{total\ integration\ appeared}$ $= \frac{2}{4.7} \times 100 = 42.6 \approx 43\%$.

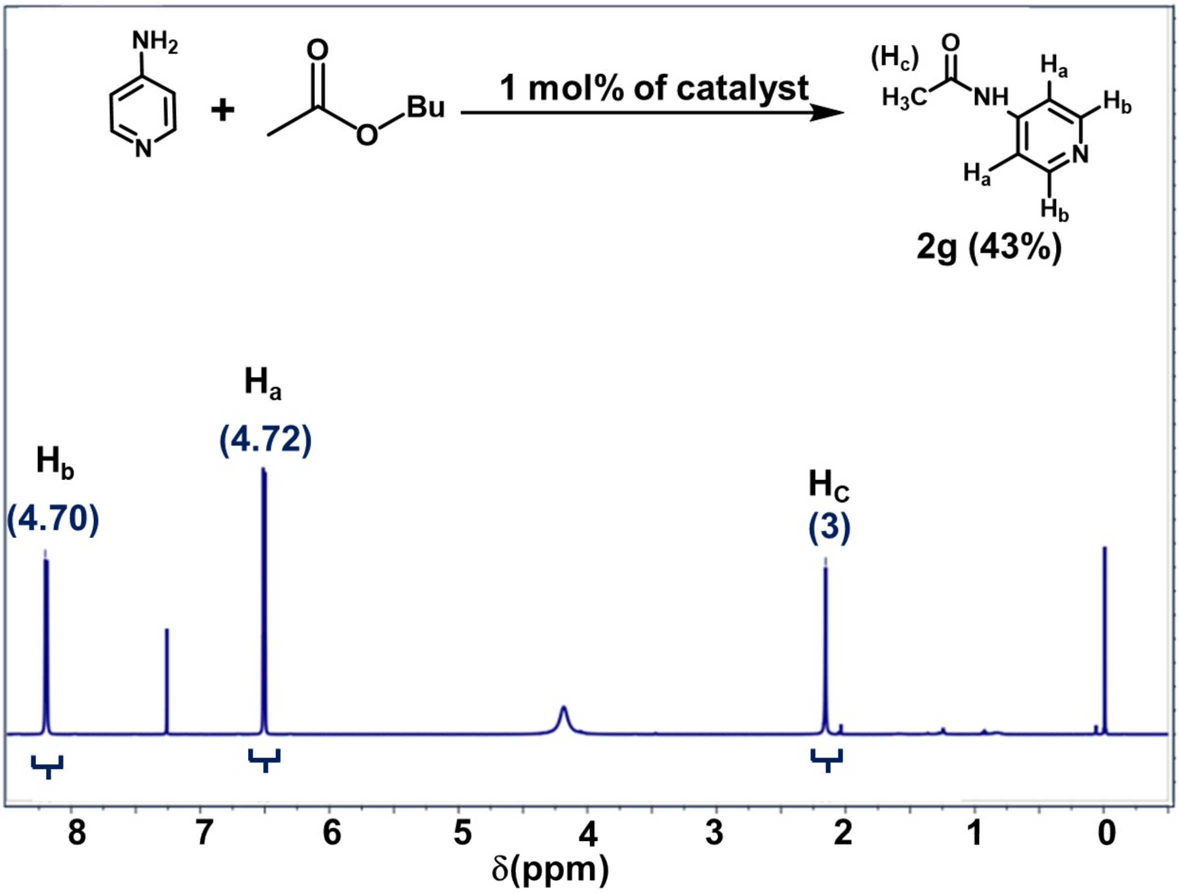

Figure 25: ^{1}H NMR of the reaction between 4-aminopyridine and butyl acetate using (TA) (1 mol%) and $[Emim]^+$ $[OAc]^-$ (1 mol%)

S7. Reaction with Aromatic Esters:

(a) Reaction of benzylamine and methyl-3-nitrobenzoate using TA (1 mol%) and $[Emim]^+$ $[OAc]^-$ (1 mol%):

In the experimental procedure, a mixture of 1gm of methyl-3-nitrobenzoate and 436 µl of benzylamine was taken in 4 ml toluene in a 50 mL round-bottom flask followed by the addition of the mixture of 3.3 mg TA and 6 µl $[Emim]^+$ $[OAc]^-$. The mixture was then heated at a temperature of 80ºC for a duration of 20 hours. After the reaction mixture was cooled at room temperature, the organic solvent was removed using a rotary evaporator. Then the resulting crude mixture was analysed via ^{1}H NMR. The integration of the peak at 3.9 ppm corresponds to the 2Hs of benzylamine (Ha), which was 0.08, and the integration of the peak at 4.3 ppm corresponds to benzyl amide (Hb), which was 1. The yield of the product was $\frac{1}{1.08} \times 100 = 92.5\%$.

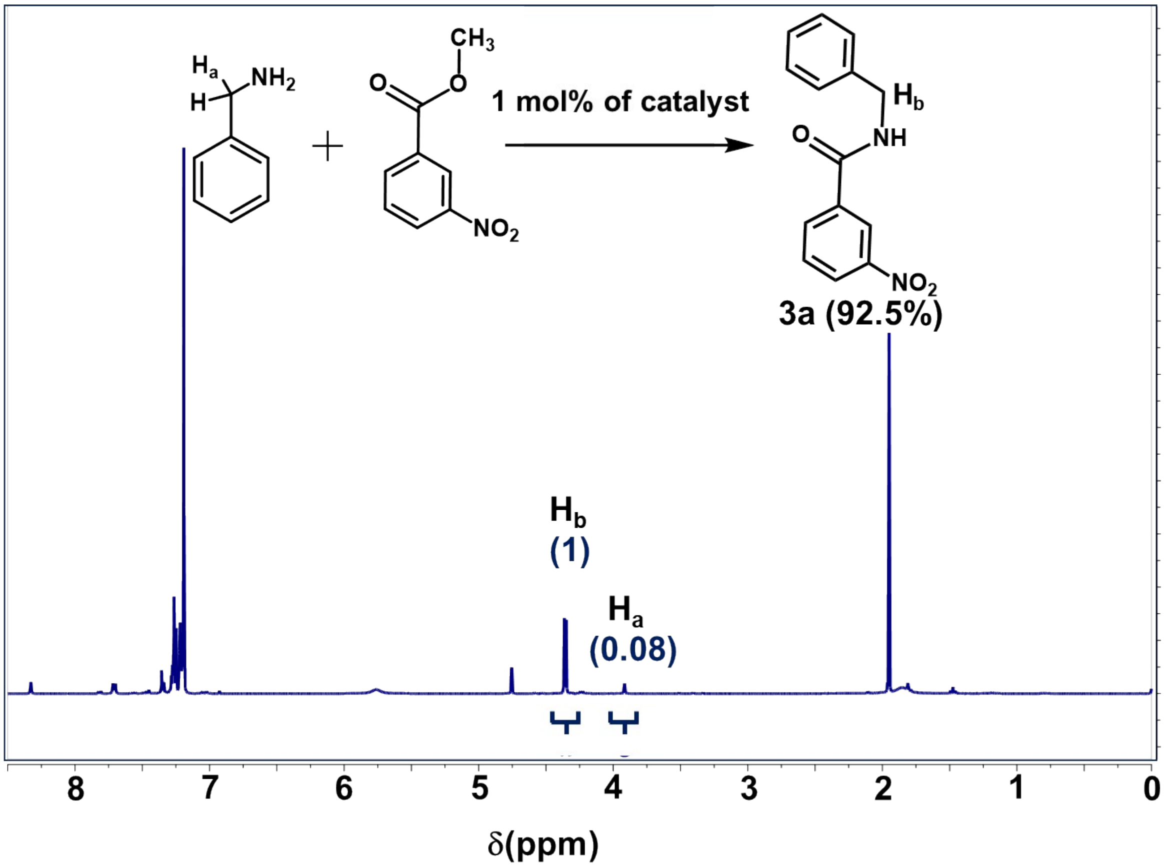

Figure 26: ^{1}H NMR of the reaction between benzylamine and methyl-3-nitrobenzoate using TA (1 mol%) and [Emim]⁺ [OAc]⁻ (1 mol%)

(b) Reaction of benzylamine and methyl benzoate using TA (1 mol%) and $[Emim]^+$ $[OAc]^-$ (1 mol%):

In the experiment, 1g methyl benzoate and 436 μl benzylamine were taken in 4 mL of toluene in a 50 mL round-bottom flask. A mixture of 3.3 mg TA and 6 μl $[Emim]^+$ $[OAc]^-$ was then added. The mixture was heated at 80ºC for 20 hours. After cooling to room temperature, the organic solvent was removed using a rotary evaporator. The crude product was analyzed using ^{1}H NMR. The product peak appeared at 4.37 ppm (2H, S) (Hb), and the unreacted reactant peak appeared at 3.82 ppm (2H, S) (H_a). Calculated product formation in the reaction was $\frac{1}{1.1} \times 100 = 90\%$.

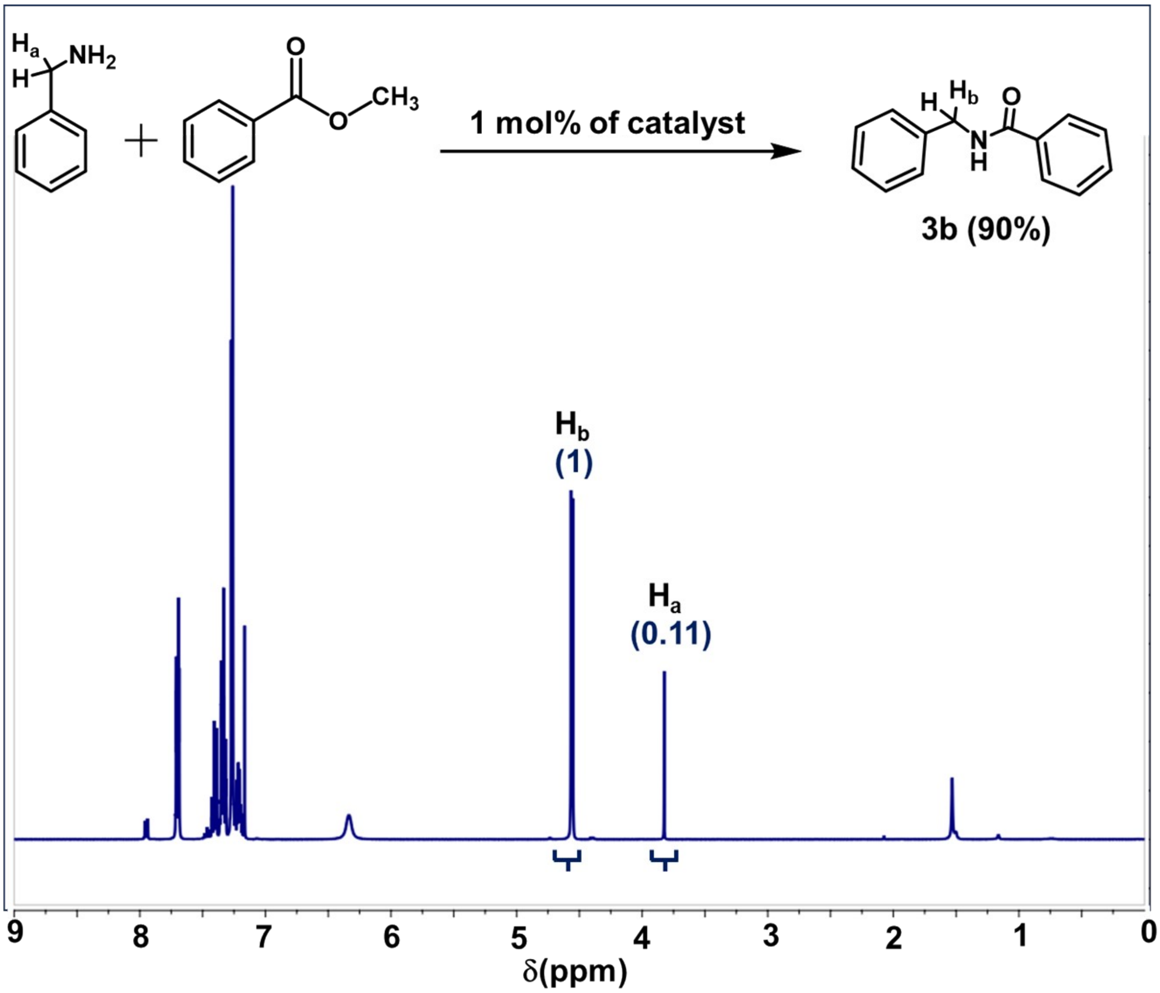

Figure 27: ¹H NMR of the reaction between benzylamine and methyl benzoate using TA (1 mol%) and $[Emim]^+$ $[OAc]^-$ (1 mol%)

S8. The reaction catalyzed by other strong acids and $[Emim]^+[OAc]^-$

(a) Reaction of benzylamine and ethyl acetate using oxalic acid (OA) (1 mol%)

In the reaction, 1 mL of ethyl acetate and 109 µl benzylamine were mixed with 0.9 mg OA and stirred at 80ºC for 20 hours. The product obtained was cooled to room temperature, and the solvent was removed using a rotary evaporator. The product obtained was analyzed through ¹H NMR. Equating the peak integrals at 3.82 ppm (representing 2Hs of benzylamine) and 4.37 ppm (corresponding to 2Hs of benzyl amide), it was observed that 17% product conversion occurred.

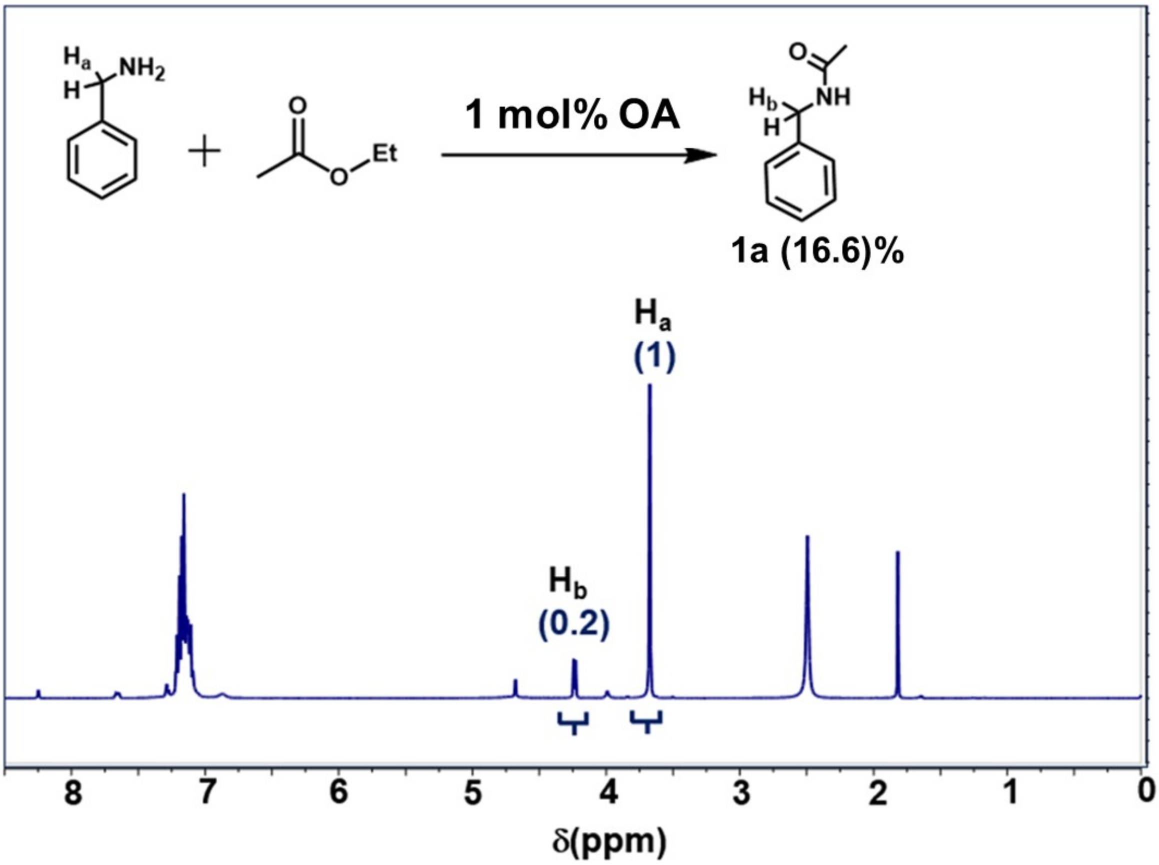

Figure 28: ¹H NMR of the reaction between benzylamine and ethyl acetate using (OA) (1 mol%)

(b) Reaction of benzylamine and ethyl acetate using malonic acid acid (MA) (1 mol%)

After successful product conversion using oxalic acid and $[Emim]^+$ $[OAc]^-$ as a catalyst, we performed the reaction by taking Malonic acid. In this reaction, we took 1 mL of ethyl acetate, and 109 µl benzylamine were mixed with 1.04 mg MA and stirred at 80ºC for 20 hours. The product obtained was cooled to room temperature, and the solvent was removed using a rotary evaporator. The product was analyzed through ^{1}H NMR. Equating the peak integrals at 3.82 ppm (representing 2Hs of benzylamine) and 4.37 ppm (corresponding to 2Hs of benzyl amide), it was observed that 27% product conversion occurred.

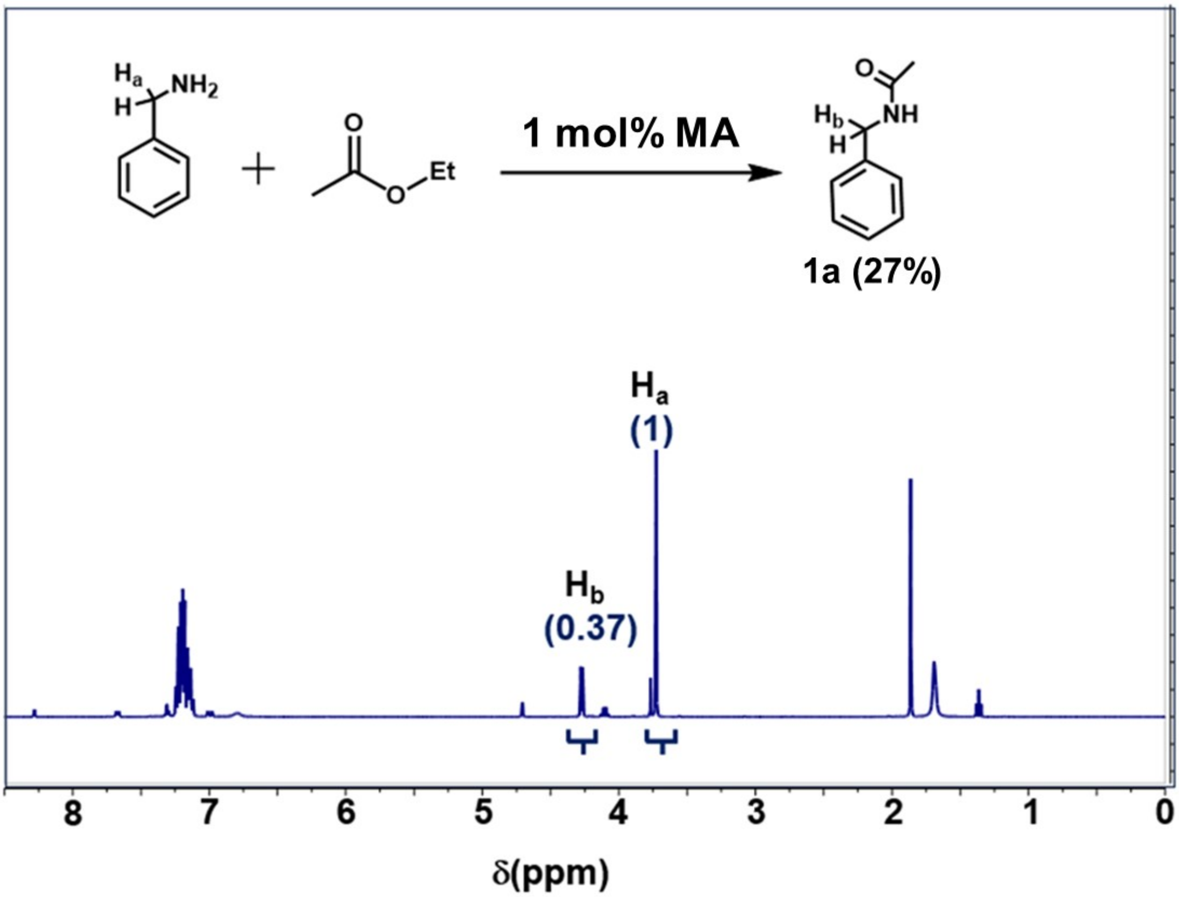

Figure 29: ^{1}H NMR of reaction of benzylamine and ethyl acetate using acid (MA) (1 mol%)

(c) Reaction of benzylamine and ethyl acetate using TA (1 mol%) and [Emim]+ [OAc]- (1 mol%):

Only 17% of the product was obtained using only 1 mol% OA in 20 hours. So, to investigate the role of $[Emim]^+$ $[OAc]^-$, another reaction was performed by taking 4 mL ethyl acetate,436 µl benzylamine, 3.6 mg OA, and 6 µl $[Emim]^+$ $[OAc]^-$ (1 mol% catalyst), by heating it at 80ºC for 20 hours. The obtained crude product was analyzed through ^{1}H NMR. The absence of a peak at 3.85 ppm indicates the absence of the reactant benzylamine. Therefore, 100% conversion occurred.

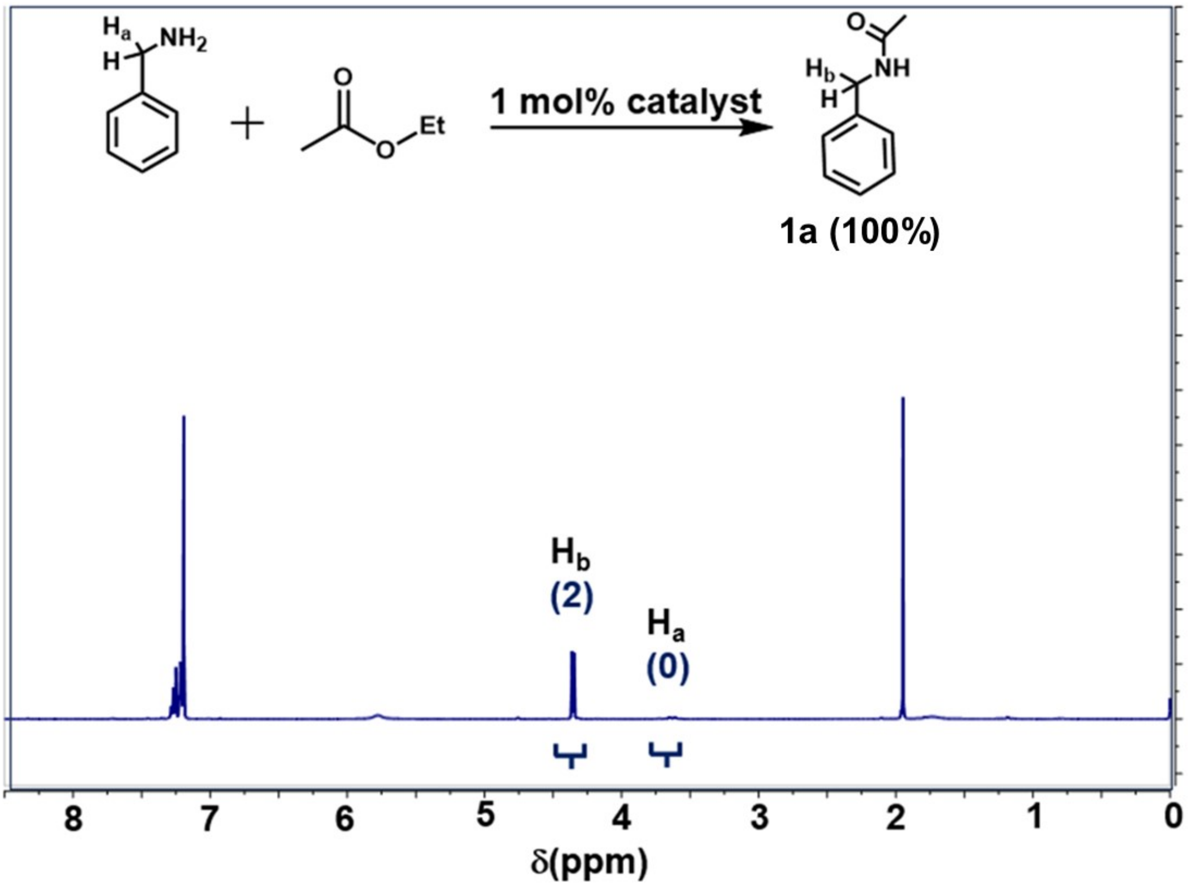

Figure 30: ^{1}H NMR of the reaction between benzylamine and ethyl acetate using TA (1 mol%) and [Emim]$^{+}$ [OAc]$^{-}$ (1 mol%)

(d) Reaction of benzylamine and ethyl acetate using MA (1 mol%) and [Emim]$^{+}$ [OAc]$^{-}$ (1 mol%):

4 mL ethyl acetate,436 µl benzylamine, 4.16 mg MA, and 6 µl [Emim]$^{+}$ [OAc]$^{-}$ (1 mol% catalyst) were taken in an RB. The reaction mixture was heated at 80ºC for 20 hours; excess solvent was removed through a rotary evaporator. The obtained crude product was analyzed through ^{1}H NMR. The absence of a peak at 3.85 ppm indicates the absence of reactant, which concludes that 100% conversion occurred.

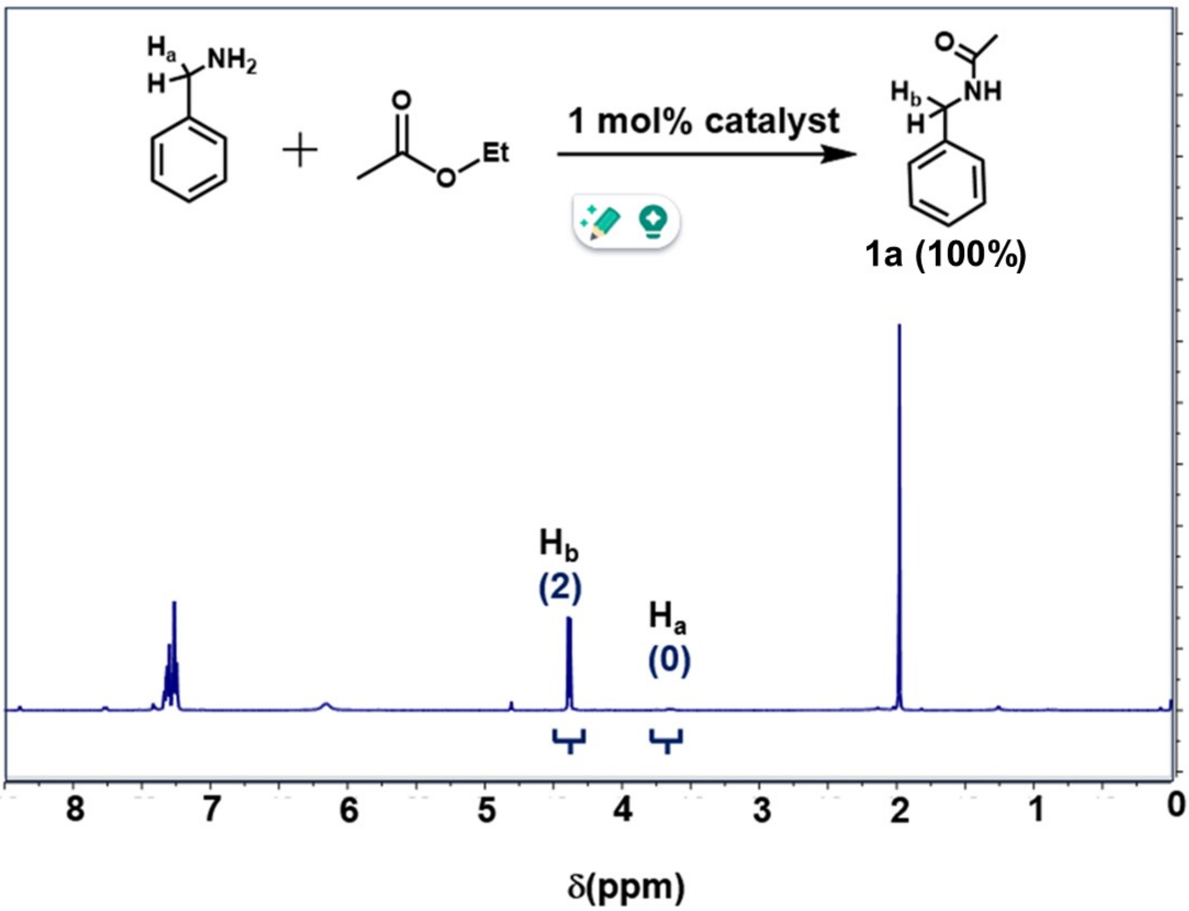

Figure 31: ^{1}H NMR of the reaction between benzylamine and ethyl acetate using MA (1 mol%) and $[Emim]^+$ $[OAc]^-$ (1 mol%)

S9. Mechanochemistry

(a) (i) Reaction of hexamethylenediamine and methyl-3-nitrobenzoate in (1:1) ratio without (TA)] and $[Emim]^+$ $[OAc]^-$ after 60 minutes: 29 mg of hexamethylenediamine and 46.5 mg methyl-3-nitrobenzoate was taken in a 10mL jar, a neat grinding was performed using a 10mm stainless steel jar with a frequency of 30 Hz. The NMR data shows the presence of a reactant peak (H_e) at 4.1 ppm. When the aromatic protons were integrated concerning the methyl proton, 4 protons were observed. This confirmed no reaction occurred in the absence of a catalyst.

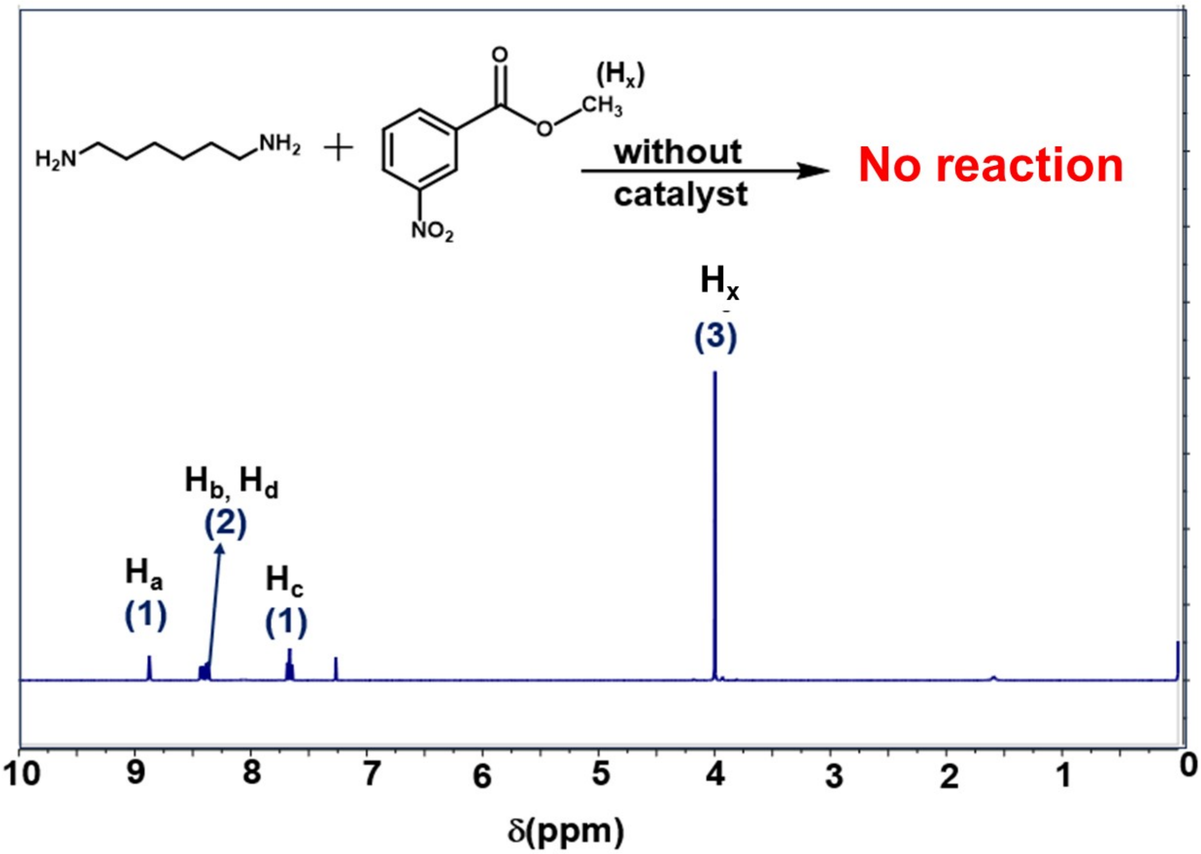

Figure 32: ^{1}H NMR of the reaction between hexamethylenediamine and methyl-3-nitrobenzoate in (1:1) ratio without (TA) and $[Emim]^+$ $[OAc]^-$ after 60 minutes

(b) Reaction of hexamethylenediamine and methyl-3-nitrobenzoate in (1:2) ratio without (TA) and $[Emim]^+$ $[OAc]^-$ after 60 minutes: 29 mg of hexamethylenediamine and 90.4 mg methyl-3-nitrobenzoate was taken in a 10mL jar, a neat grinding was performed using a 10mm stainless steel jar with a frequency of 30 Hz. The NMR data shows the presence of a reactant peak (H_e) at 4.1 ppm. When the aromatic protons were integrated with respect to the methyl proton, 4 (H_a (1), $H_{b,\,d}$ (2), H_c (1)) protons were observed. This confirmed no reaction occurred in the absence of a catalyst.

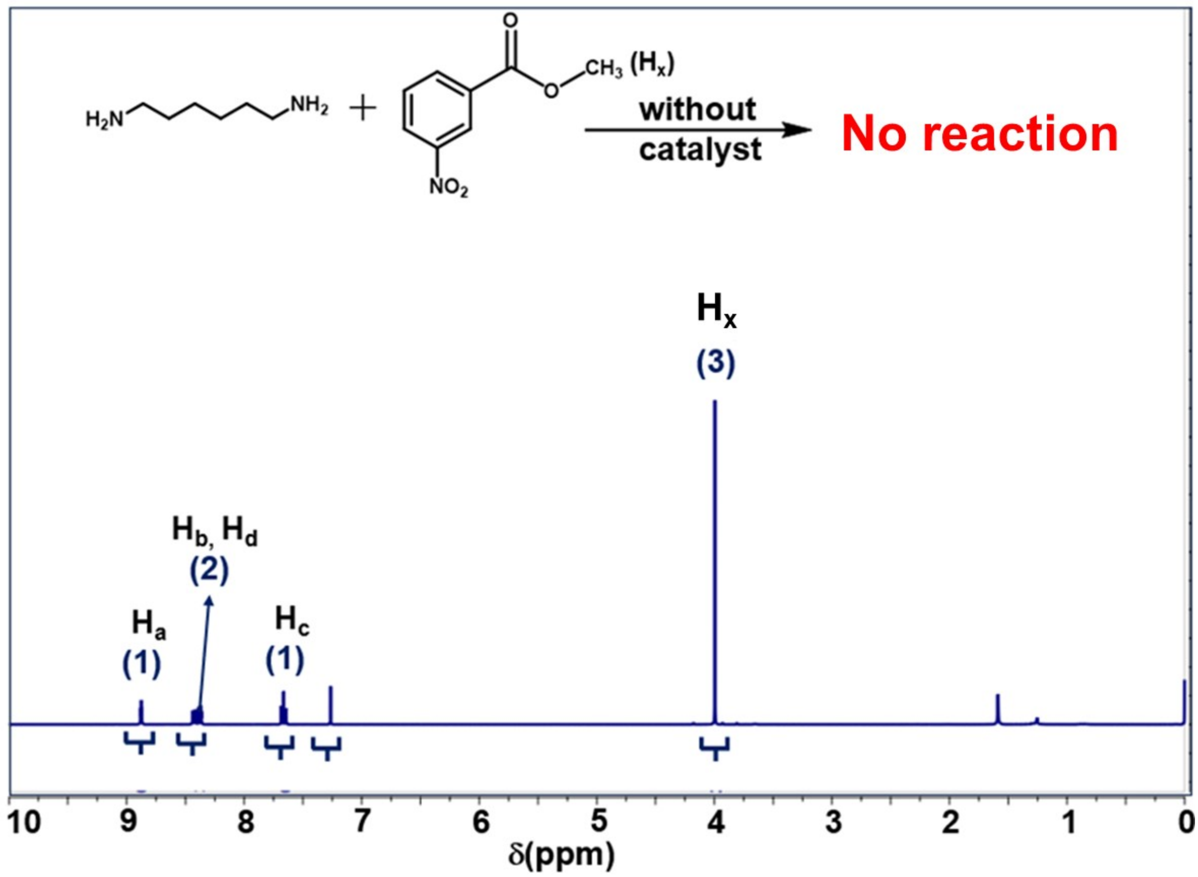

Figure 33: ^{1}H NMR of the reaction between hexamethylenediamine and methyl-3-nitrobenzoate in (2:1) ratio without (TA) and [Emim]$^+$ [OAc]$^-$ after 60 minutes

(c) Reaction of hexamethylenediamine and methyl-3-nitrobenzoate using (TA) (30 mol%) and [Emim]$^+$ [OAc]$^-$ (30 mol%) after 30 minutes:

A mixture was prepared by combining 45.2 mg of methyl-3-nitrobenzoate and 29 mg of hexamethylenediamine in a 10 ml jar with a 10 mm ball. To this mixture, 8.9 mg of a TA and 15 µl of ionic liquid ([Emim]$^+$ [OAc]$^-$) were added. The mixture was then ground at 30 Hz for 30 minutes. After grinding, the resulting crude mixture was analyzed using ^{1}H NMR spectroscopy. The disappearance of the H_x proton suggested a 100% product formation. However, the 4 protons peaks were observed in the aromatic region, with respect to 12 protons in the aliphatic region. This result suggested a 1:1 product formed when the reaction was done for 30 minutes.

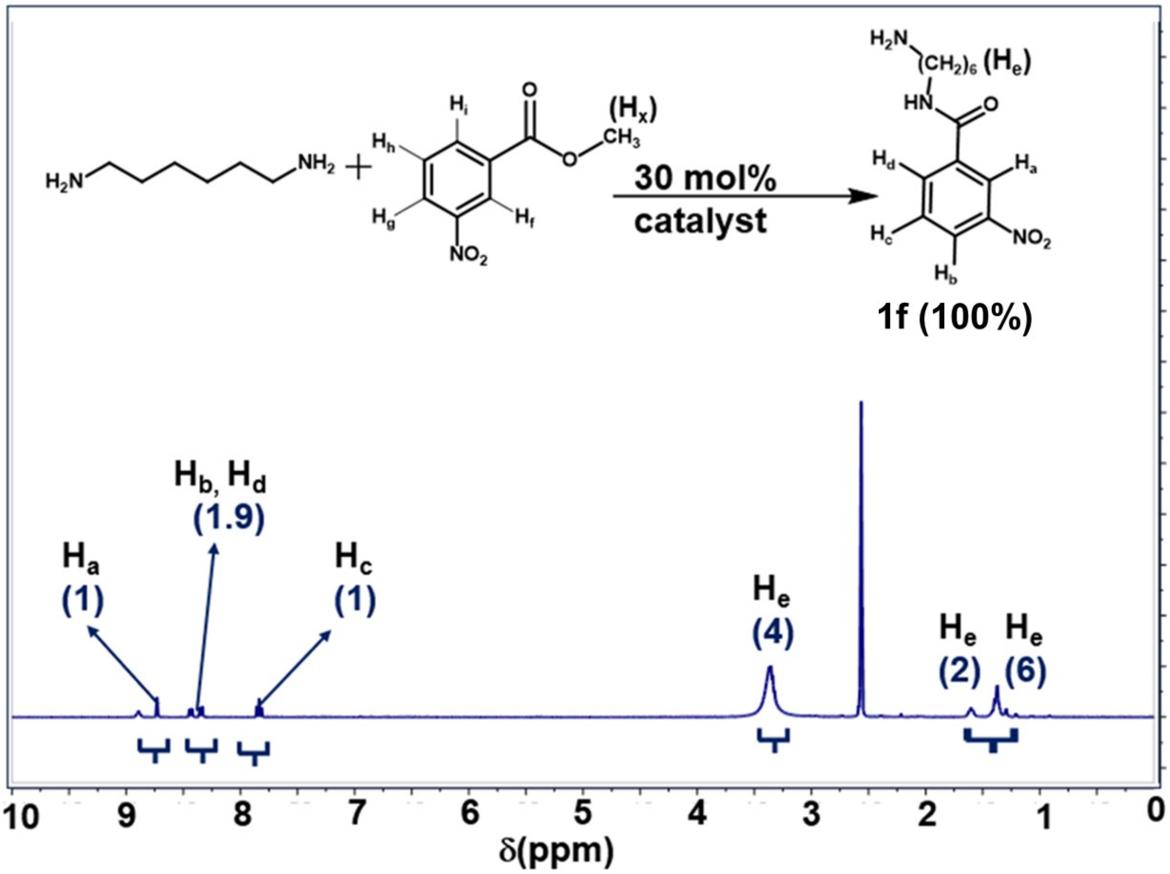

Figure 34: ¹H NMR of the reaction between hexamethylenediamine and methyl-3-nitrobenzoate using (TA) (30 mol%) and [Emim]$^+$ [OAc]$^-$ (30 mol%) after 30 minutes

(ii) Mass spectra (LC-MS): Mass spectra of the product obtained from the above reaction were collected by dissolving the product (~1mg) in 2 mL of methanol; the mass data was collected using the Shimadzu LCMS 8040 instrument. The mass at m/z= 266 confirmed the formation of the product 1f.

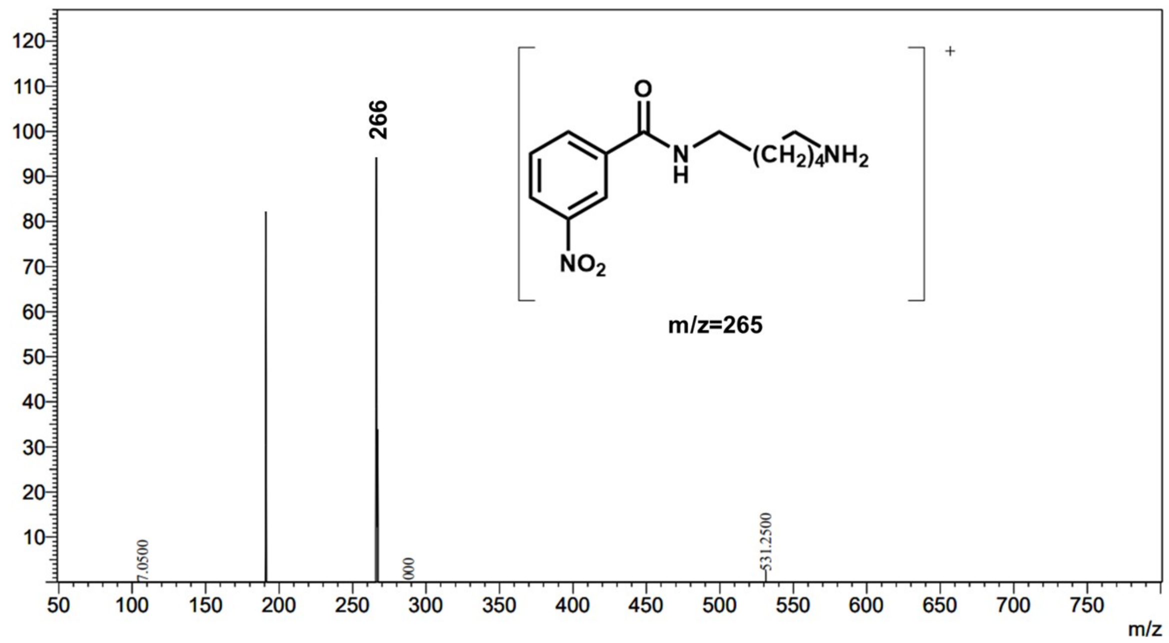

Figure 35: Mass spectra of mechanochemical reaction of hexamethylenediamine, methyl-3-nitrobenzoate, using (TA) (30 mol%) and $[Emim]^+$ $[OAc]^-$ (30 mol%) after 30 minutes.

(d) Reaction of hexamethylenediamine and methyl-3-nitrobenzoate using (TA) (15 mol%) and $[Emim]^+$ $[OAc]^-$ (15 mol%) after 30 minutes:

A mixture was prepared by combining 45.2 mg of methyl-3-nitrobenzoate and 29 mg of hexamethylenediamine in a 10 ml jar with a 10 mm ball. To this mixture, 4 mg of a TA and 9 µl of ionic liquid ($[Emim]^+$ $[OAc]^-$) were added. The mixture was then ground at 30 Hz for 30 minutes. After grinding, the resulting crude mixture was analyzed using 1H NMR spectroscopy. The disappearance of the H_x proton suggested a 100% product formation. Similar to the last result, only the 4 protons peaks were observed in the aromatic region, with respect to 12 protons in the aliphatic region, resulting in a 1:1 product formation with 15 mol% catalysts.

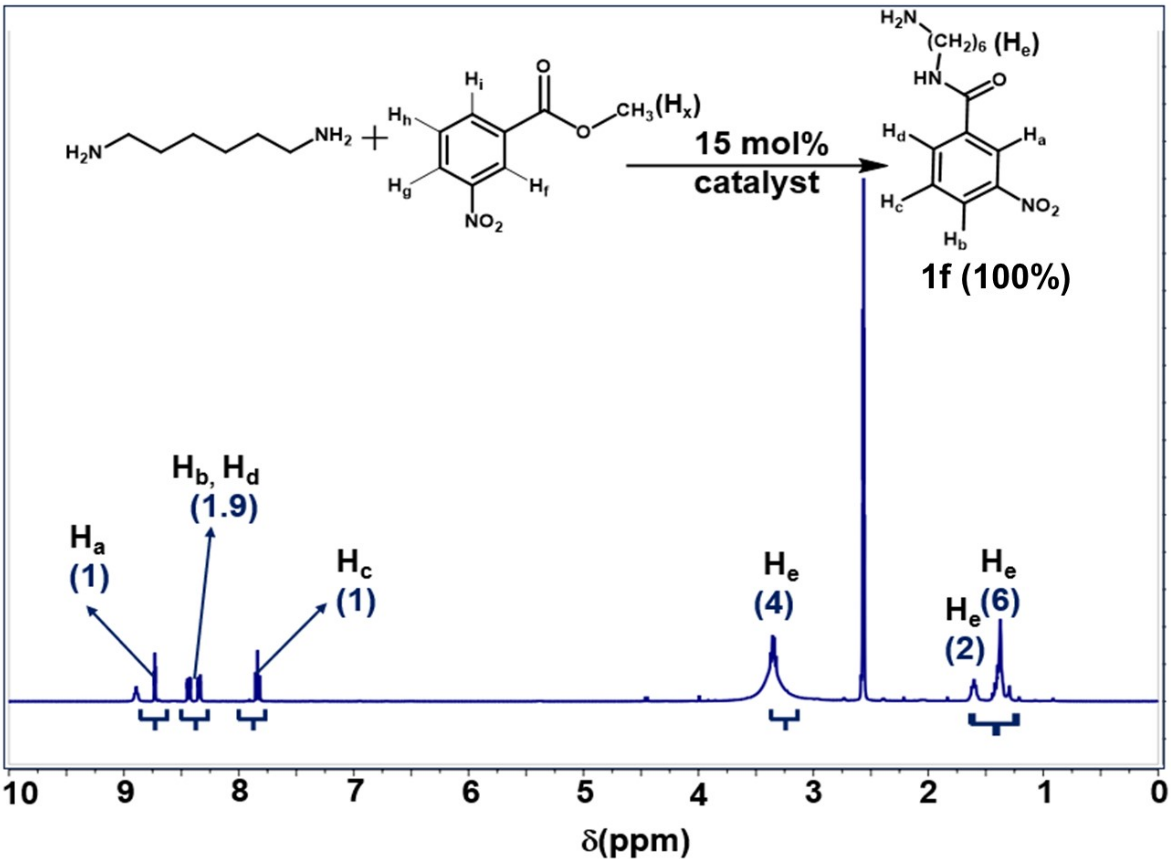

Figure 36: ^{1}H NMR of the reaction between hexamethylenediamine and methyl-3-nitrobenzoate using (TA) (15 mol%) and $[Emim]^+$ $[OAc]^-$ (15 mol%) after 30 minutes

(e) Reaction of hexamethylenediamine and methyl-3-nitrobenzoate using (TA) (5 mol%) and $[Emim]^+$ $[OAc]^-$ (5 mol%) after 30 minutes:

In the experimental procedure, a mixture of 45.2 mg of methyl-3-nitrobenzoate and 29 mg of hexamethylenediamine was taken in a 10 ml jar containing 1 ball of 10mm size. Then 1.2 mg TA and 1.8 µl $[Emim]^+$ $[OAc]^-$ was added. This mixture was then ground at a 30 Hz frequency for 30 minutes. Then, the resulting crude mixture was analyzed via ^{1}H NMR. The disappearance of the H_x proton suggested a 100% product formation. 1:1 product formed with 5 mol% catalysts while the mixture was ground for 30 minutes.

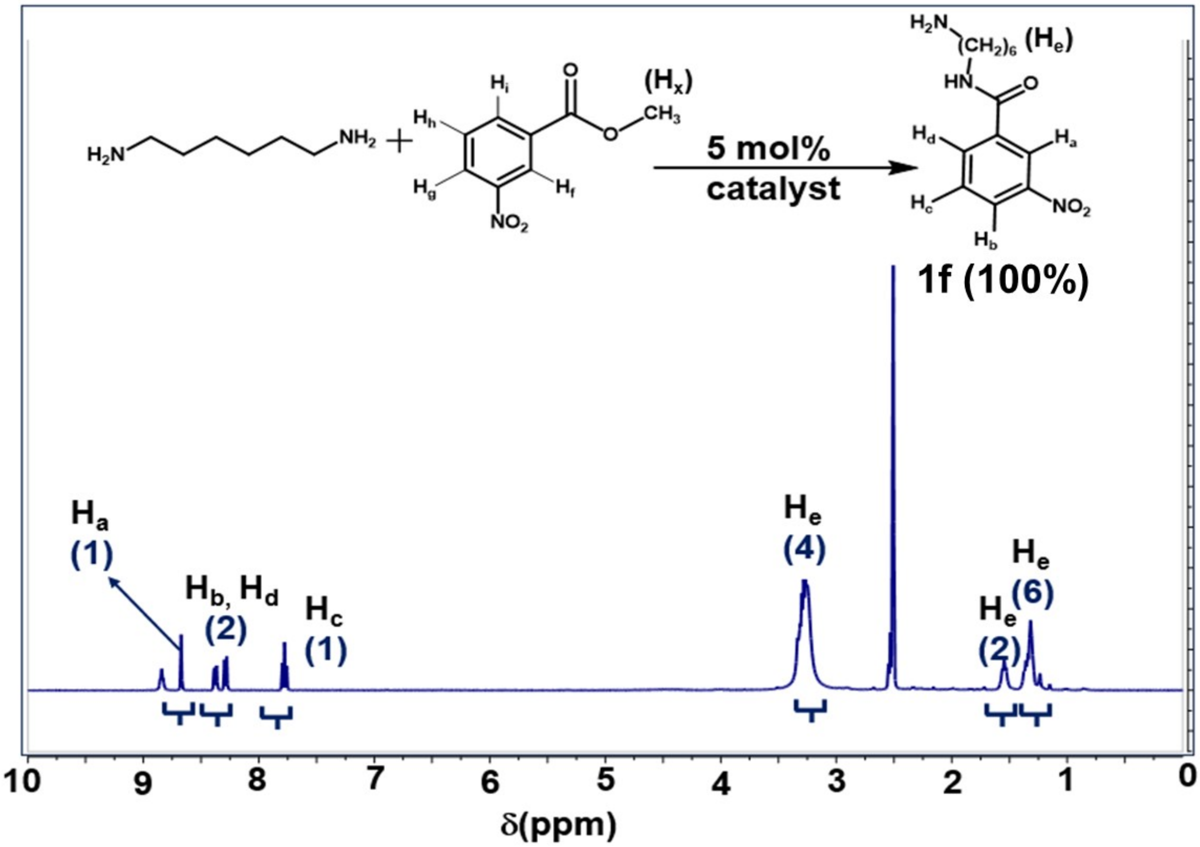

Figure 37: ^{1}H NMR of the reaction between hexamethylenediamine and methyl-3-nitrobenzoate using (TA) (5 mol%) and [Emim]$^+$ [OAc]$^-$ (5 mol%) after 30 minutes

(f) Reaction of hexamethylenediamine and methyl-3-nitrobenzoate in (1:2) ratio using (TA) (30 mol%) and [Emim]$^+$ [OAc]$^-$ (30 mol%) after 30 minutes:

90.4 mg of methyl-3-nitrobenzoate and 29 mg of hexamethylenediamine were taken in a 10 ml stainless steel jar with two 10 mm balls. To this mixture, 8.9 mg of a TA and 15 µl of ionic liquid ([Emim]$^+$ [OAc]$^-$) were added. The mixture was then ground at 30 Hz for 30 minutes. After grinding, the resulting crude mixture was analyzed using ^{1}H NMR spectroscopy. The disappearance of the H_x proton suggested a 100% product formation. However, the 4 protons peaks were observed in the aromatic region, with respect to 12 protons in the aliphatic region. This result suggested a 1:1 product formed when the reaction was done for 30 minutes.

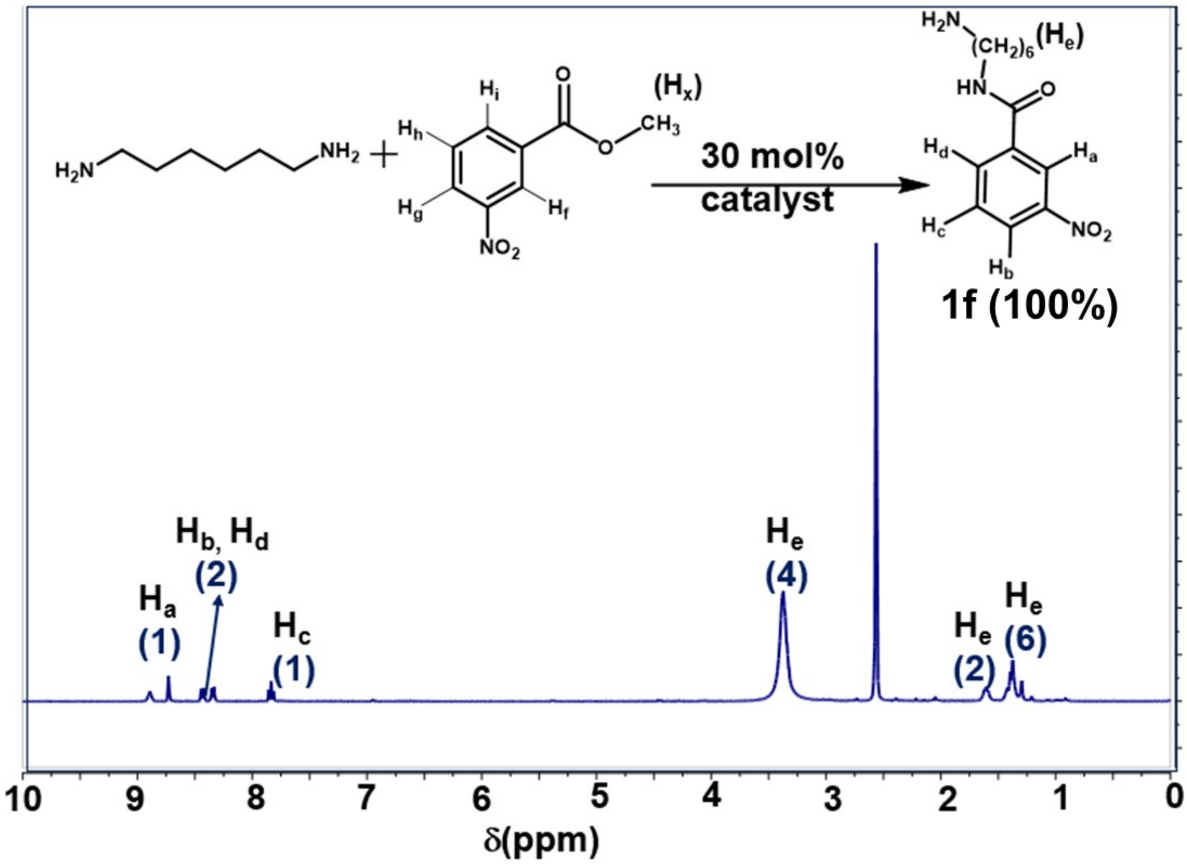

Figure 38: ^{1}H NMR of the reaction between hexamethylenediamine and methyl-3-nitrobenzoate in (1:2) ratio using (TA) (30 mol%) and $[Emim]^+$ $[OAc]^-$ (30 mol%) after 30 minutes

(g) Reaction of hexamethylenediamine, methyl 3-nitrobenzoate, and 3,5-dichloro benzaldehyde using (TA) (5 mol%) and $[Emim]^+$ $[OAc]^-$ (5 mol%) after 30 minutes

(i) NMR spectra

45.2 mg of methyl-3-nitrobenzoate, 29 mg of hexamethylenediamine, and 43.5 mg of 3,5dichlorobenzaldehyde were taken in a 10 ml stainless steel jar with two 10 mm balls. To this mixture, 1.2 mg of a TA and 1.8 µl of ionic liquid ($[Emim]^+$ $[OAc]^-$) were added. The mixture was then ground at 30 Hz for 30 minutes. After grinding, the resulting crude mixture was analyzed using ^{1}H NMR spectroscopy. The disappearance of aldehyde proton H_d (10-10.5 ppm) confirmed the complete consumption of aldehyde. However, the reaction was not completed with the conditions applied, confirmed by the presence of an ester peak at 3.9 ppm (H_c). Some of the aliphatic protons appeared at 2.58 ppm near the d^6-DMSO peak, and some aliphatic peaks appeared at 3.3 ppm near the water's proton peak. So, it was difficult to get the actual integration values for the aliphatic region. Mass spectra were collected to confirm the

product further. To confirm the product conversion, the ester proton (Hc for 3H) was integrated as 3, and when the imine proton of the product H_a was integrated, it was found as 1.3 (for 1 H). The calculated yield of the reaction was 56%.

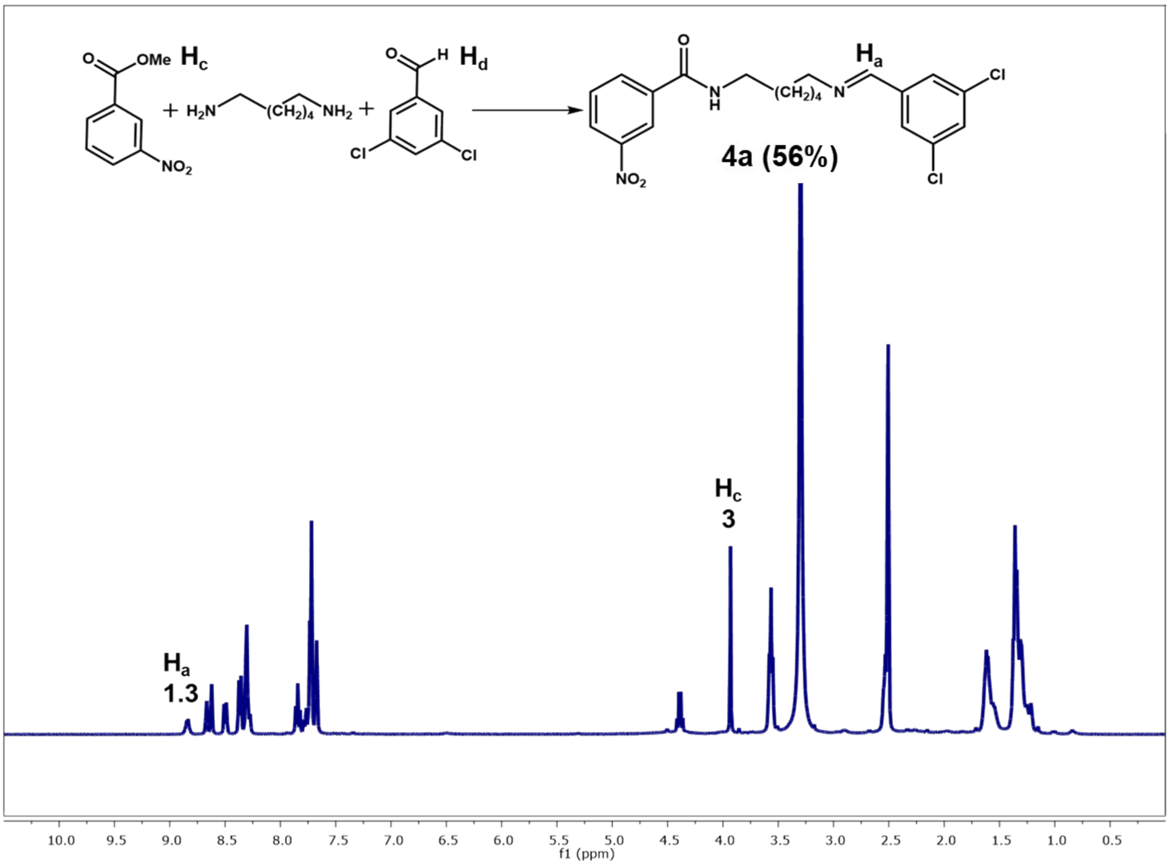

Figure 39:^{1}H NMR of mechanochemical reaction between hexamethylenediamine, methyl-3-nitrobenzoate, and 3,5-dichlorobenzaldehyde in (1:1:1) ratio using (TA) (5 mol%) and [Emim]$^+$ [OAc]$^-$ (5 mol%) after 30 minutes.

(ii) Mass spectra: A significant peak in the mass spectra was observed at m/z=422 with 100 % abundance corresponding to the product. Another two peaks were observed at m/z=277 and 266 due to the fragments shown in Figure 34.

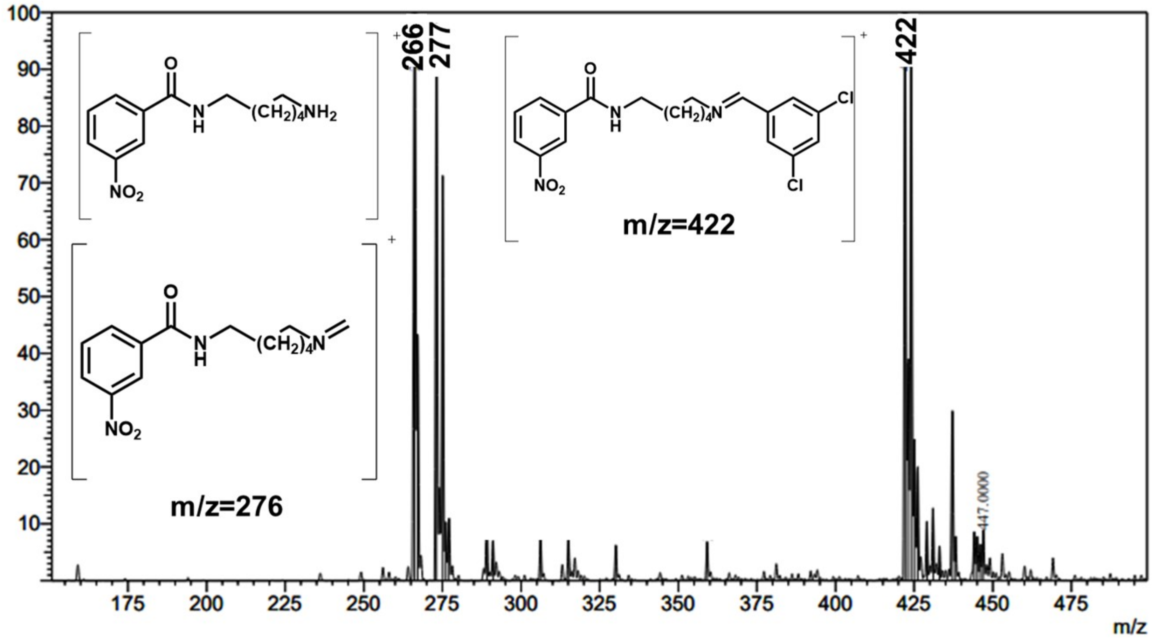

Figure 40: Mass spectra of mechanochemical reaction of hexamethylenediamine, methyl-3-nitrobenzoate, and 3,5dichlorobenzaldehyde in (1:1:1) ratio using (TA) (5 mol%) and $[Emim]^+$ $[OAc]^-$ (5 mol%) after 30 minutes.

(h) Reaction of hexamethylenediamine, methyl-3-nitrobenzoate, and terephthalaldehyde using (TA) (5 mol%) and $[Emim]^+$ $[OAc]^-$ (5 mol%) after 30 minutes

45.2 mg of methyl-3-nitrobenzoate, 29 mg of hexamethylenediamine, and 16.7 mg of terephthalaldehyde were taken in a 10 ml stainless steel jar with two 10 mm balls. To this mixture, 1.2 mg of a TA and 1.8 µl of ionic liquid ($[Emim]^+$ $[OAc]^-$) were added. The mixture was then ground at 30 Hz for 30 minutes. After grinding, the resulting crude mixture was analyzed using ^{1}H NMR spectroscopy. The disappearance of aldehyde proton H_x (10-11 ppm) confirmed the complete consumption of aldehyde. Some aliphatic protons came at 2.58 ppm near d^6-DMSO and 3.3 ppm near the water peak. So, getting the actual integration values for the aliphatic region was challenging. Careful observation of the NMR spectra suggested the reactant didn't completely convert to the product. When the product proton peak H_a (for 2Hs) was integrated as 2, the reactant's proton (H_z) (for 2Hs) integration appeared as 0.15. the calculated yield of the product was 87%. Mass spectra were collected to confirm the product further.

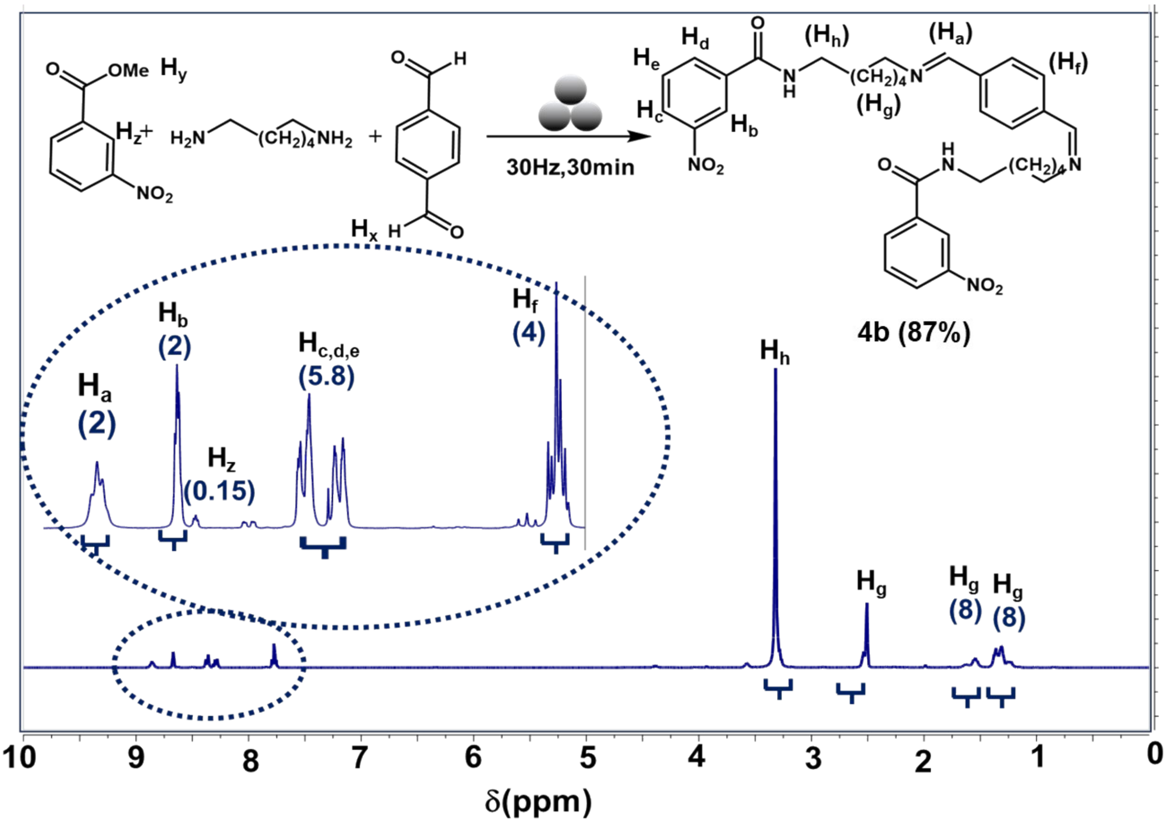

Figure 41: ^1H NMR of mechanochemical reaction of hexamethylenediamine, methyl-3-nitrobenzoate, and terephthalaldehyde *in (2:2:1) ratio using (TA) (5 mol%) and $[Emim]^+$ $[OAc]^-$ (5 mol%) after 30 minutes.*

(ii) Mass spectra (LC-MS): Mass spectra of the product were collected dissolving in methanol. A significant peak in the mass spectra was observed at m/z=629, which corresponded to the desired product. Other significant peaks m/z=480, 380 were observed due to the fragments below.

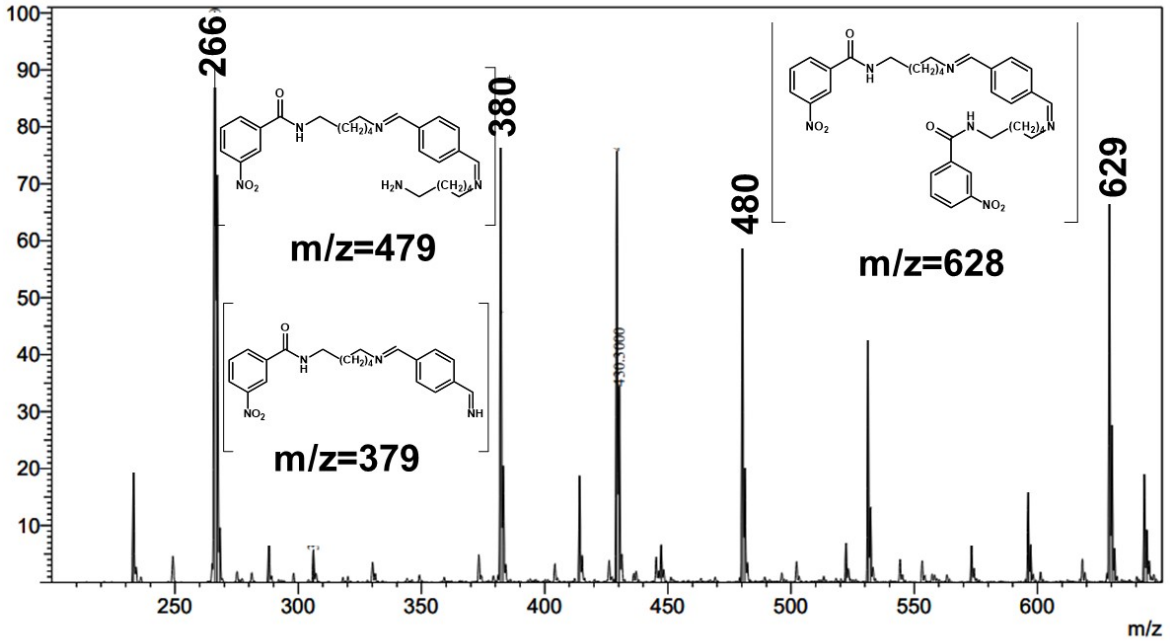

Figure 42: Mass spectra of the mechanochemical reaction of hexamethylenediamine, methyl-3-nitrobenzoate, and terephthalaldehyde in (2:2:1) ratio using (TA) (5 mol%) and $[Emim]^{+}$ $[OAc]^{-}$ (5 mol%) after 30 minutes.

S10. Table 1: Average Reaction yield and standard deviation:

**Compound**	**Time hour**	**TA (mol %)**	**$[Emim]^{+}$ $[OAc]^{-}$ (mol %)**	**Reaction 1 yield (%)**	**Reaction 2 yield (%)**	**Reaction 3 yield (%)**	**Average yield %**	**Standard deviation**
1a (1)	20	0	0	4.7%	3.8%	2.9%	3.8%	0.7
1a (2)	20 hours	0	10	18%	17.3%	--------	17.6%	0.3
1a (3)	20 hours	0	1	6%	5.6%	8.2%	6.6%	1.0
1a (4)	20 hours	10	0	27%	24.2%	--------	25.6%	1.1

1a (5)	20 hours	1	0	20%	19.3%	--------	19.6%	0.3
1a (6)	4 hours	10	10	18%	15.9%	--------	16.9%	0.9
1a (7)	8 hours	10	10	46%	42%	46.8%	44.9%	1.8
1a (8)	12 hours	10	10	61%	67%	70%	66%	3.2
1a (9)	16 hours	10	10	86%	92%	90%	89.3%	2.2
1a (10)	20 hours	10	10	100%	100%	100%	100%	0
1a (11)	20 hours	1	1	100%	95%	97.5%	97.5%	1.8
1b	20 hours	1	1	90%	92%	--------	91%	0.8
1c.	20 hours	1	1	41.5%	46%	--------	43.7%	1.8
1d.	20 hours	1	1	100%	97.8%	--------	98.9%	0.9
1e.	20 hours	1	1	100%	98.7%	--------	99.3%	0.5
1f.	30 minutes	5	5	100%	98%	--------	99%	0.8
2a.	24 hours	1	1	100%	99%	--------	99.5%	0.4
2b.	24 hours	1	1	100%	97.5%	--------	98.7%	1.0
2c.	24 hours	1	1	100%	98.5%	--------	99.2%	0.6

2d.	24 hours	1	1	100%	99.3%	--------	99.6%	0.3
2e.	24 hours	1	1	100%	98.8%	--------	99.4%	0.5
2f.	24 hours	1	1	100%	97.4%	--------	98.7%	1.0
2g.	24 hours	1	1	43%	39%	--------	41%	1.6
3a.	20 hours	1	1	92.5%	89%	--------	90.7%	1.4
3b.	20 hours	1	1	90%	88%	--------	89%	0.8

S11: Comparison of this in-situ acetic acid catalyzed N-acylation of amines with other methods: N-acetylation of amines remained an immensely important reaction due to enormous application in several industries. The traditional methods incorporated mostly metal catalysts (BEMP, K_3PO_4, $La(OTf)_3$, NaOMe, and CaI_2) or hygroscopic chemicals that are often expensive or toxic in nature**.** However, William and their co-workers utilized acetic acid successfully to form amide bonds using less reactive esters with an amine. Since a mixture of TA with $[Emim]^+[OAc]^-$ can lead to the formation of in situ acetic acid, we have compared the synthetic outcome of some of the direct acetic acid additions with those of in-situ acetic acid in the table below.

Table: A comparison of the current method with the previous acetic acid-based method

**Starting material (amine)**	**Mol% of direct acetic acid**	**Yield (%)**	**Mol% of is situ catalyst**	**Yield (%)**
Benzylamine	5	92	1	97.5 ± 1.8
N-methyl-1-phenylmethanamine	50	91	1	99.. ± 0.5
Aniline	250	88	1	99.5 ± 0.4
4-methoxyaniline	250	96	1	99.2 ± 0.6

S12. Separation of the catalyst from the reaction mixture and catalytic recyclability: For the isolation of the product, we performed a workup using ethyl acetate and water through liquid-liquid extraction. The [TA]·[Emim]$^{+}$ + [TA]$^{-}$ salt dissolved in the aqueous layer, while the product was found to remain in the organic layer, confirmed through TLC.

To get an idea about the recyclability of the catalyst, a reaction was performed with benzylamine and ethyl acetate in the presence of 1 mol% catalyst. After 20 hours, an aliquot of the reaction was taken out, and the conversion of the reaction was checked through NMR. After confirming a quantitative conversion, another set of reactants (benzylamine and ethyl acetate mixture) was added to the previous reaction mixture without more catalysts. This cycle was repeated three times. In the first cycle, the absence of a reactant peak (Ha) at 3.82 ppm confirmed a 100% conversion occurred. In the second cycle, 73.5% product conversion was observed by equating the peak integrals at 3.80 ppm (representing 2Hs of benzylamine) and 4.35 ppm (corresponding to 2Hs of benzyl amide). Integrating the product (H_b)(int=1) and reactant peak (H_a) (int=0.36), The product conversion calculated was $\frac{1}{1.36} \times 100 =$ 73.5%. After taking out the aliquot of the second cycle, another set of reactants was added to the reaction mixture. After 20 hours, the NMR data of the mixture was collected after workup and drying. Comparing the reactant 3.8 ppm (corresponding to the 2Hs of benzylamine) and product peak 4.35 ppm (corresponding to 2Hs of benzyl amide), it was observed that 60.9% of the product was formed. The product conversion was calculated by integrating the product peak (H_b) at 1 and the reactant peak (H_a) at 0.64, resulting in a conversion of $\frac{1}{1.64} \times 100 =$ 60.9%. and showed the recyclability catalyst in the N-acetylation reaction. The 1H NMR of the given sample is provided below.

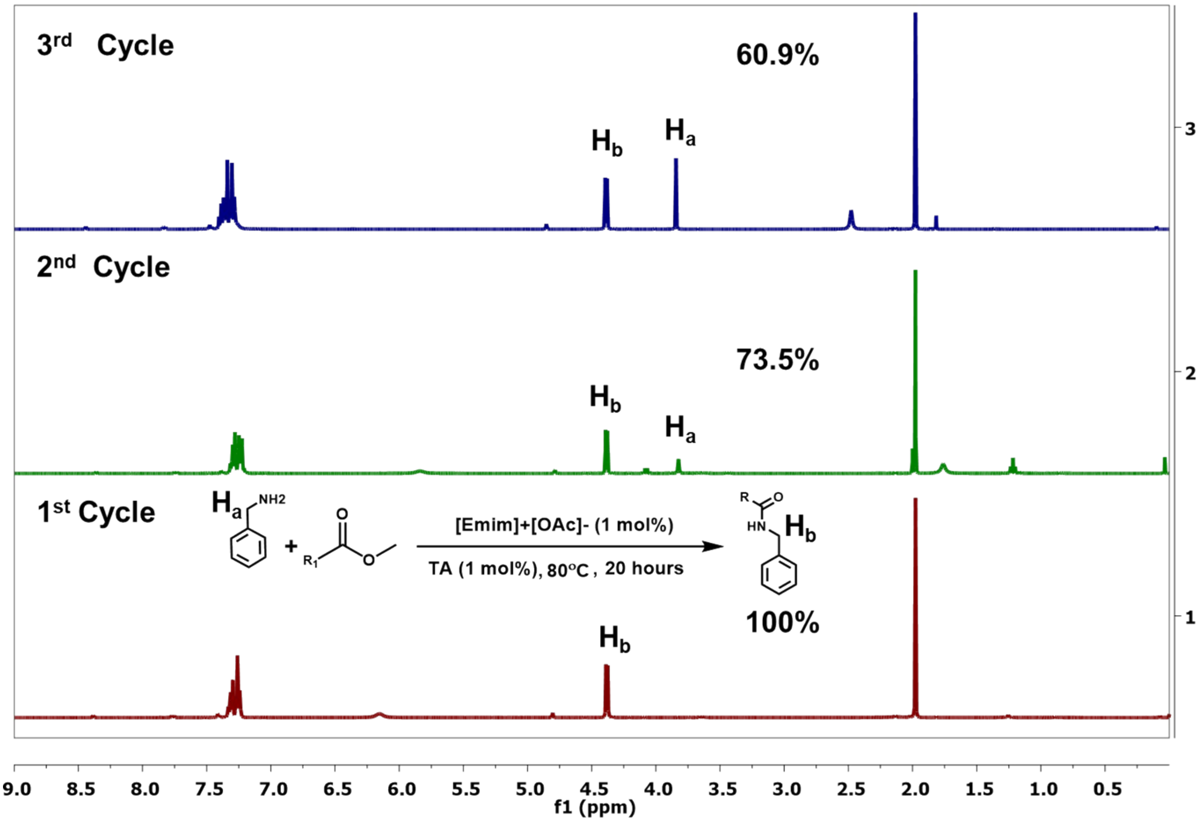

Figure 43: Recyclability of the catalyst (1st cycle red, 2nd cycle green, 3rd cycle blue).
